# Refining and optimising a behavioural intervention to support endocrine therapy adherence (ROSETA) in UK women with breast cancer: protocol for a pilot fractional factorial trial

**DOI:** 10.1136/bmjopen-2022-069971

**Published:** 2023-02-03

**Authors:** Samuel G Smith, Sophie M C Green, Rachel Ellison, Robbie Foy, Christopher D Graham, Ellen Mason, David P French, Louise H Hall, Hollie Wilkes, Emma McNaught, Erin Raine, Rebecca Walwyn, Daniel Howdon, Jane Clark, Nikki Rousseau, Jacqueline Buxton, Sally J L Moore, Catherine Parbutt, Galina Velikova, Amanda Farrin, Michelle Collinson

**Affiliations:** 1 Academic Unit of Primary Care, Leeds Institute of Health Sciences, University of Leeds, Leeds, UK; 2 Complex Interventions Division, Clinical Trials Research Unit, Leeds Institute of Clinical Trials Research, University of Leeds, Leeds, UK; 3 School of Psychology, Queens University Belfast, Belfast, UK; 4 School of Psychological Sciences, Division of Psychology and Mental Health, University of Manchester, Manchester, UK; 5 Academic Unit of Health Economics, Leeds Institute of Health Sciences, University of Leeds, Leeds, UK; 6 Department of Clinical and Health Psychology, Leeds Teaching Hospitals NHS Trust, Leeds, UK; 7 Surgical, Diagnostic and Devices Division, Clinical Trials Research Unit, Leeds Institute of Clinical Trials Research, University of Leeds, Leeds, UK; 8 Medicines Management and Pharmacy Services, Leeds Teaching Hospitals NHS Trust, Leeds, UK; 9 Leeds Institute of Medical Research, University of Leeds, Leeds, UK

**Keywords:** Breast tumours, PREVENTIVE MEDICINE, PUBLIC HEALTH

## Abstract

**Introduction:**

Women with breast cancer who do not adhere to adjuvant endocrine therapy (AET) have increased risks of mortality and recurrence. There are multiple barriers to AET adherence, including medication side-effects, beliefs about medication, memory and psychological distress. We developed four intervention components, each targeting a different barrier. This pilot trial is part of the preparation phase of the Multiphase Optimisation Strategy, and aims to establish key trial parameters, establish intervention component adherence, establish availability and feasibility of outcome and process data, estimate variability in planned outcome measures and estimate cost of developing and delivering each intervention component.

**Methods and analysis:**

The four intervention components are as follows: short message service text reminders (target: memory); a written information leaflet (target: medication beliefs); a guided self-help Acceptance and Commitment Therapy programme (target: psychological flexibility to reduce distress) and a self-management website (target: side-effect management). To evaluate the feasibility of recruitment, acceptability of the intervention components and the availability of outcome data, we will conduct a multisite, exploratory pilot trial using a 2^4-1^ fractional factorial design, with a nested process evaluation. We will randomise 80 women with early-stage breast cancer who have been prescribed AET to one of eight experimental conditions. This will determine the combination of intervention components they receive, ranging from zero to four, with all conditions receiving usual care. Key outcomes of interest include medication adherence and quality of life. Progression to the optimisation phase will be based on predefined criteria for consent rates, patient adherence to intervention components and availability of medication adherence data.

**Ethics and dissemination:**

The study was reviewed by the Wales Research Authority Research Ethics Committee 3 (21/WA/0322). Written informed consent will be obtained from all patients before randomisation. The results of this trial will be disseminated in a peer-reviewed journal.

**Trial registration number:**

ISRTCN10487576.

**Strengths and limitations of this study**

- This design will estimate the individual and combined effects of multiple intervention components.
- This multisite pilot will recruit women through three routes.
- Progression to an optimisation trial will be decided using a priori criteria.
- A mixed-methods process evaluation will assess intervention acceptability and fidelity.
- The pilot will not examine effectiveness of the intervention components

## Introduction

Approximately 55 000 UK women are diagnosed with breast cancer each year, with 11 000 cancer-related deaths.^1^ Most breast cancers are oestrogen receptor positive (ER+) tumours. Adjuvant endocrine therapies (AETs) such as selective oestrogen receptor modulators (eg, tamoxifen) and aromatase inhibitors (eg, letrozole) improve outcomes for women with ER+ tumours. Low adherence to AET is linked with higher rates of recurrence and all-cause mortality.^2–9^ A meta-analysis reported 31%–73% of breast cancer survivors are adherent to AET,^10^ and stopping AET is most common within 12 months of initiation.^11–13^ Unintentional non-adherence, such as forgetting, may be more common than intentionally missing doses.^14–16^

An overview of behavioural support programmes for women using AET reported at least 15 ongoing trials targeting AET adherence.^17^ Among adequately powered trials of AET interventions, there are few positive findings.^18–20^ This may be explained by the reliance on interventions involving the provision of written information; which may address some adherence barriers, but is unlikely to address the complex issues underpinning most medication taking behaviours.^17 21^ As with many long-term conditions where treatment non-adherence is a problem, intervention programmes may require multiple active components to target a range of key determinants.

Existing AET adherence trials have used single-arm or parallel group randomised designs. While parallel group trials are suitable for definitively evaluating a complex intervention against a suitable comparator, they provide little information on the relative contributions of individual components.^22^ Undertaking separate or multiarm randomised controlled trials (RCTs) to evaluate individual intervention components would be prohibitively expensive and time-consuming, and would not allow estimates of interactions between intervention components. Alternative trial designs are needed to improve our understanding of complex interventions, and increase the efficiency of definitive evaluations.

The Multiphase Optimisation Strategy (MOST) is a framework that includes an optimisation phase between developing and evaluating a complex intervention.^22^ Within this optimisation phase, MOST advocates the use of highly efficient experimental designs to provide empirical data on the main effects and interactions of intervention components. This information is used to identify the optimal combination of intervention components to produce the desired outcome, without exceeding key constraints (eg, cost).^23^ The optimised intervention package can be evaluated in a definitive RCT. The process of optimisation aims to yield more effective, affordable, scalable and efficient intervention packages compared with alternative approaches.^24 25^ Given the complexity of adherence behaviours, the lack of information available about individual intervention component efficacy, and the need to address an important clinical problem efficiently, we used the MOST framework to guide our approach to intervention optimisation.

As part of the preparatory phase of MOST, we used Intervention Mapping to develop a theoretically informed intervention package.^17 26^ We identified several modifiable factors associated with AET adherence,^27–30^ selecting four of the strongest determinants as intervention targets.^17^ Four intervention components were developed or adapted to address these targets. The current trial represents the next step in the preparation phase of MOST; examining the feasibility of an optimisation trial of the proposed intervention package in women with early-stage breast cancer. Our objectives are to: (i) establish eligibility, recruitment, retention and follow-up rates; (ii) establish intervention component adherence; (iii) establish availability and feasibility of outcome and process data; (iv) estimate variability of planned outcome measure(s) and (v) estimate cost of developing and delivering each intervention component.

We will also include a nested process evaluation to further inform the decision of whether to undertake a future optimisation trial, and how the intervention components could be adapted based on intervention fidelity, acceptability and trial experience. A full protocol of this process evaluation is published elsewhere.^31^

## Methods and analysis

### Design

This is a multisite, exploratory pilot trial using a 2^4-1^ fractional factorial design with a nested mixed-methods process evaluation. We will randomise 80 women with early-stage breast cancer to one of eight experimental conditions, which will determine the intervention components they receive (table 1). There will be an equal chance of allocation to each group. Each component has two levels (present or absent). All women will receive usual care. Women will be followed-up at 2 and 4 months, and key outcomes include medication adherence (self-report and National Health Service (NHS) data) and quality of life. The trial adheres to the Standard Protocol Items: Recommendations for Interventional Trials recommendations^32^ (online supplemental appendix 1), and the Consolidated Standards of Reporting Trials extension for pilot and feasibility trials^33^ (online supplemental appendix 2) and the intervention components are described using the TIDieR checklist^34^ (online supplemental appendix 3).

10.1136/bmjopen-2022-069971.supp1 Supplementary data

10.1136/bmjopen-2022-069971.supp2 Supplementary data

10.1136/bmjopen-2022-069971.supp3 Supplementary data

**Table 1:** 2^4-1^ fractional factorial design for pilot trial

Condition	Usual care	Text reminders	Information leaflet	Acceptance and commitment therapy	Side-effect website	Randomisation sample size
**1**	Yes	Yes	Yes	Yes	Yes	10
**2**	Yes	Yes	Yes	No	No	10
**3**	Yes	Yes	No	Yes	No	10
**4**	Yes	Yes	No	No	Yes	10
**5**	Yes	No	Yes	Yes	No	10
**6**	Yes	No	Yes	No	Yes	10
**7**	Yes	No	No	Yes	Yes	10
**8**	Yes	No	No	No	No	10

### Setting

Participant identification and recruitment will occur at five UK NHS hospitals. Sites with existing or planned implementation of interventions designed to improve adherence to AET will be excluded. The site must have access to a Health and Care Professional Council (HCPC) registered practitioner psychologist (Clinical, Health or Counselling Psychologist) or a UK Council for Psychotherapy (UKCP) registered psychotherapist. The site must have access to video conferencing software or a telephone to deliver the Acceptance and Commitment Therapy (ACT) sessions.

### Participants

Adult women using tamoxifen, raloxifene, anastrozole, letrozole or exemestane as AET for early-stage (1–3a) breast cancer are eligible (table 2).

**Table 2:** Eligibility criteria for participation in the ROSETA pilot trial

Inclusion criteria	Exclusion criteria
An informed consent form (signed and dated) Capacity to provide informed consent Women with early stage (1–3a) breast cancer according to the TNM/American Joint Committee on Cancer (AJCC) staging system. *Note: Women being treated for a second primary breast cancer or a breast cancer local recurrence are eligible for the study, providing the most recent cancer is being treated with adjuvant endocrine therapy and they meet all eligibility criteria. Women with bilateral breast cancer are permitted, providing at least one breast is affected by hormone receptor-positive disease.* Aged≥18 years at time of screening for ROSETA’s pilot study Have sufficient proficiency in English to be able to adhere to all intervention components and data collection required Treated with curative intent Completed their hospital-based treatment (eg, surgery, radiotherapy and/or chemotherapy) for the current breast cancer within the last 12 months. *Note: Women are still eligible for the study if they are being treated with monoclonal antibody-based therapy such as trastuzumab, kadcyla, pertuzumab and phesgo* Currently prescribed oral adjuvant Hormone Therapy (tamoxifen, raloxifene, anastrozole, letrozole and exemestane) The participant is willing to complete the study questionnaires.* The participant is willing to be audio recorded during the therapy sessions* The participant is willing and able to attend all ACT sessions either via video conference or telephone* The participant is willing and able to complete home practice tasks* Access to a mobile phone to receive SMS messages* Willing to receive frequent SMS messages* Access to a computer or smart device that can access the internet*	Stopped taking adjuvant hormone therapy if it is clinically contraindicated according to clinical recommendation Women with metastatic breast cancer Currently or recently (last 6 months) involved in a similar research study where medication adherence is a primary outcome* Currently attending psychotherapy/psycho-oncology/psychology/counselling services, for any clinical reason* Need for treatment for a severe mental health disorder or crisis, which is likely to interfere with participation (eg, active psychosis, bipolar disorder, significant issues with addiction or self-harm or expressing active suicidal ideation with active plans and intent*) *Note: If concerned about the possible presence of risk of suicidal ideation with active plans and intent, then this can be assessed with the following questions, with patients ineligible if they answer ‘yes’ to 5c*. *Recently (in the last month):* *Have you had any thoughts about ending your life?* *(if yes) Have you thought about how you might go about it?* *(if yes) Do you intend to carry out this plan?* Patients with a scheduled date for breast reconstruction surgery that is within their intervention delivery and follow-up period. *Note: Women planning to have a breast reconstruction but who have not scheduled a date for surgery are permitted* Auditory problems that would prevent the patient from participating in a telephone or video call, or hearing audio clips*

*Source data for these items will be either partially or completely patient self-report.

ACT, Acceptance and Commitment Therapy; ROSETA, refining and optimising a behavioural intervention to support endocrine therapy adherence; SMS, short message service.

### Trial processes

Participants will be identified via three routes. Route 1 involves the research nurse (RN) prospectively screening patient records prior to clinic visits. Route 2 will identify patients who have self-referred to their care team to discuss problems with their medications. Route 3 involves the RN retrospectively searching patient records to identify patients who have completed hospital treatment. All patients should have completed their last hospital treatment within 12 months of randomisation.

Sites will complete a screening form for all identified patients. Anonymised data on age, ethnicity, staging, tumour type and whether a patient is randomised will be captured. Patients who are not randomised will have the reason they are ineligible or declined participation recorded.

For recruitment route 1, a member of the care team will introduce a RN to the patient, and offer the option of receiving trial documents by email. Eligible patients may consent immediately or after further consideration. In route 2, the patient’s oncologist will conduct an initial review of eligibility and, where participants are willing, provide their contact details to a RN who will telephone them and email trial documents. Interested patients contact the RN by telephone and consent is taken. For route 3, the RN will post or email the patient an invitation letter and a copy of the patient information sheet. For all routes, a RN will confirm eligibility and record consent (online supplemental appendix 4). Eligibility and consent discussions, and completion of baseline measures may take place in person or remotely.

10.1136/bmjopen-2022-069971.supp4 Supplementary data

Authorised individuals at NHS sites will be given access to the Clinical Trials Research Unit (CTRU) online database and will enter site and participant details, and confirm eligibility and informed consent. After recording consent, the RN will register the participant and a link to the baseline questionnaire will be sent automatically from the CTRU. Once completed, the RN will notify the CTRU that a participant can be randomised, and an authorised member of the CTRU will perform the randomisation. Participants will be randomised to one of eight experimental conditions (table 1). Stratified permuted block randomisation will ensure experimental conditions are well balanced for the recruitment route. Participants, therapists, RNs, participants’ GPs and CTRU staff (including the Chief Investigator) will not be blinded to the randomised allocation. The RN will notify participants of their allocation.

We will ask participants to complete electronic follow-up questionnaires at 2 and 4 months postrandomisation. Non-responders will receive telephone, email and/or SMS message reminders.

Patients may withdraw from the SMS and ACT intervention components, completing the questionnaires, or from the collection of data from NHS Digital and data processing. The local principal investigator may decide a participant should be withdrawn if they have become unsuitable for the trial. The analysis will use data collected up to the date of withdrawal of consent.

### Usual care

All participants will receive treatment as usual, which will be the standard care offered to women at this stage of their breast cancer treatment. It is likely to differ by recruiting site. Most women will be invited to attend an end of treatment summary meeting with a breast care nurse. The content of treatment as usual programmes and information on hospital-based services accessed will be reported through patient self-report and site-level report. Providers of usual care will not be blinded.

### Intervention components

#### SMS intervention component

Memory problems are common in women with breast cancer,^35 36^ and forgetting is a cause of non-adherence to AET.^14 15 37 38^ Forming medication taking habits can reduce reliance on memory and help sustain behaviour change.^39–42^ Mobile phone messaging interventions are a potential cost-effective approach to promote habit formation.^43–45^ This has not been widely tested in cancer patients.^46^

We codeveloped, with behaviour change experts and women affected by breast cancer, an intervention component involving SMS reminders to support habit formation of daily medication taking and associated behaviours (eg, ordering prescriptions).^47^ The SMS messages are based on six behaviour change techniques theorised to support the development of habitual behaviours.^48^ The SMS messages will be sent by CTRU and delivered over 4 months, commencing up to 1 week following randomisation. Forty-three SMS messages will be delivered to participants in the same order; three opening messages, daily messages for 2 weeks, two messages per week for 8 weeks, weekly messages for 6 weeks and one closing message. A message will be sent monthly informing participants that they can stop the SMS messages by emailing the trial team.

#### Medication beliefs intervention component

Women using AET report low perceived need for therapy, while also citing unfounded concerns about the medication, which could impact adherence.^28 29 49–52^ Qualitative studies have highlighted a demand for accurate information about AET to overcome unfounded concerns.^27–29^

We developed a six-page patient information leaflet to target AET medication beliefs. The leaflet explains how AET works supplemented by diagrams, information about AET benefits and side effects, answers to common concerns and quotes and photos of breast cancer survivors. Leaflet content is informed by existing qualitative research, views from our patient and public involvement group, the Necessity Concerns Framework^52^ and the Common-Sense Model of Self-regulation.^53^ Participants receiving this component will be emailed the leaflet by the NHS site, after randomisation.

#### Acceptance and commitment therapy (ACT) intervention component

Approximately half of women with breast cancer experience higher distress than the general population.^30 54 55^ Distress is associated with adherence to AET,^30 56^ and interventions targeting this barrier could support medication adherence behaviours. Any prospective intervention would need to target the factors contributing to distress, including fear of recurrence, difficulties ‘returning to normal’ and distress caused by side-effects.^27 57 58^

The ACT component is a guided self-help programme targeting psychological distress, by promoting psychological flexibility.^59 60^ We used an adapted version of a guided self-help ACT intervention shown to be effective for reducing distress in people with muscle diseases.^61^ It consists of five telephone or video call sessions with a therapist. The modules suggest four ACT-based skills; mindfulness, unhooking, following values and living beyond labels, that aim to enhance well-being and reduce distress. Each module consists of a participant manual containing information about an ACT skill, alongside home practice exercises and audio files.

The participant will see the same therapist for each session. The first session will take place within 4 weeks of randomisation and consists of a 15 min introduction. The 3 weekly sessions with the therapist will last 25 min each. Within these sessions, the therapist and the patient will discuss the previous week’s module, and reflect on the participant’s experience of using the skills. Following each session, the therapist will email the patient the next module’s materials. One week after the fourth module, there will be a closing 15 min support session. Participants allocated to receive ACT can cease participation in that component by informing the therapist or contacting the trial team.

#### Self-management website intervention component

AET side effects (eg, hot flushes and arthralgia^62–67^) reduce quality of life.^62 68–72^ Side-effects also likely impact medication adherence,^68 73–75^ although evidence is mixed.^76^ Oncologists perceive side-effects to be a major deterrent to AET adherence,^77^ which is corroborated by patients.^27 78^ Women feel unsupported in managing side-effects,^27 57 78^ and would like clearer information on self-management strategies.^58 79^ We undertook an umbrella review of systematic reviews and clinical guidelines on self-management strategies for common AET side-effects.^80^ This review informed the creation of a patient-facing website to support side-effect management in women using AET.

Website creation was also based on suggestions made by patients and healthcare professionals attending a codevelopment workshop.^81^ The website includes videos of patient stories, sign-posting for further information and information on common AET side-effects. Participants randomised to the website component will be sent login details by the NHS site following randomisation.

### Measures

The primary outcomes for the study are the progression criteria (consent rate, component adherence and medication adherence measures). Secondary outcomes include quality of life, costs, psychological flexibility, beliefs about medications, habit formation, psychological distress, safety, acceptability, trial experience and fidelity. Tables 3 and 4 summarise timing of data collection for each assessment.

**Table 3:** Summary of assessments

Assessment	Source	Method of completion	Timeline				
			Screening	Baseline	2 months	4 months	End of trial
**Participant**							
Screening	Screening CRF	Research nurse/delegate	X				
Contact details	CRF	Research nurse/delegate		X	CTRU to be notified of changes		
Demographics including comorbidities (Charlson Comorbidity Index)	CRF	Self-completion/research nurse/delegate		X			
Eligibility (including inclusion and exclusion criteria)	CRF	PI/research nurse/PIs delegate		X			
Consent	Consent form	Self-completion/RN/delegate over the telephone		X			
Randomisation	CRF/CTRU online system	Research nurse/delegate		X			
Prescribing/dispensing data	NHS digital	PIs delegate		X	X	X	
MMAS-8	Questionnaire	Self-completion		X	X	X	
Voils DOSE-non-adherence measure—extent scale	Questionnaire	Self-completion		X	X	X	
EORTC QLQ-C30	Questionnaire	Self-completion		X	X	X	
EORTC QLQ-BR45	Questionnaire	Self-completion		X	X	X	
EORTC-IL133	Questionnaire	Self-completion		X	X	X	
EQ-5D-5L	Questionnaire	Self-completion		X	X	X	
Intervention costs	NHS reference costs/PSSRU	PIs delegate				X	
MPFI (short form)	Questionnaire	Self-completion		X	X	X	
BMQ-AET	Questionnaire	Self-completion		X	X	X	
SRBAI	Questionnaire	Self-completion		X	X	X	
DASS-21	Questionnaire	Self-completion		X	X	X	
McGill QoL revised	Questionnaire	Self-completion		X	X	X	
Safety reporting	CRF	Research nurse/PI/delegate			X		
End of trial data	CRF	Research nurse					X
End of trial site data	CRF	Research nurse					X
SMS delivery and receipt data	Online system	Delegate			X	X	
Opt-out of SMS messages	Online system	Delegate		X (collected throughout)			
Information leaflet delivery	Site recorded	Research nurse		X			
ACT session attendance, number of cancelled/missed sessions	CRF	Clinician		X (collected throughout)			
Engagement with ACT module materials (participant manual, associated audio files and home practice tasks)	CRF	Clinician		X (collected after each therapy session)			
Engagement with ACT module audio files	Questionnaire	Self-completion				X	
Completion of ACT home practice tasks	Questionnaire	Self-completion				X	
Dates of ACT sessions	CRF	Clinician		X (collected throughout)			
Printing of ACT module booklets	Questionnaire	Self-completion				X	
Delivery of website login details	Site recorded	Research nurse		X			
Website usage	Website online system	Delegate			X	X	
Self-reported receipt of SMS, information leaflet, ACT modules and website	Questionnaire	Self-completion				X	
Self-reported reading of SMS, information leaflet, ACT module participant manuals and website	Questionnaire	Self-completion				X	
Acceptability questionnaire	Questionnaire	Self-completion				X	
Questionnaire return	Online system	Delegate		X	X	X	
Trial withdrawals	CRF	Delegate	X (collected throughout)				
Study Participant Feedback Questionnaire (SPFQ)	Questionnaire	Self-completion		X	X	X	
Fidelity of receipt and enactment of intervention components	Semi-structured interview	Interview				X	X
Barriers and facilitators to trial participation and response rates	Semi-structured interview	Interview					X
Barriers and facilitators to recruitment	Questionnaire	Research nurse self-completion					X
Research nurse demographics	CRF	Research nurse		X			
UK Cancer Costs Questionnaire	Questionnaire	Self-completion			X	X	
Questionnaire reminder preference	Questionnaire	Self-completion				X	

ACT, Acceptance and Commitment Therapy; BMQ-AET, Beliefs about Medication Questionnaire - Adjuvant Endocrine Therapy; CRF, Case Report Form; CTRU, Clinical Trials Research Unit; DASS-21, Depression Anxiety Stress Scales; DOSE-nonadherence, Domains of Subjective Extent of non-adherence; EORTC QLQ, European Organisation for Research and Treatment of Cancer Quality of Life Questionnaire; EQ-5D-5L, EuroQol 5 domains 5 levels; MMAS, Morisky Medication Adherence Scale; MPFI, Multi-Dimensional Psychological Flexibility Inventory; NHS, National Health Service; PI, Principal Investigator; PSSRU, Personal Social Sciences Research Unit; QoL, Quality of Life; RN, Research nurse; SMS, short message service; SRBAI, Self-Report Behavioural Automaticity Index.

**Table 4:** Summary of ACT therapist assessments

Assessment	Source	Method of completion	Post-therapist training	Pretrial delivery	First session	Second session	Third session	Fourth session	Fifth session	End of trial
Consent	Consent form	Therapist	X							
Demographics	CRF	Therapist	X							
ACT-FM Competency Assessment (Therapist stance subscale)	Questionnaire	ACT trainer			X					
Number of booster training sessions required	Questionnaire	ACT trainer		X (collected throughout)						
ACT-FM therapist stance subscale	Questionnaire	Independent expert reviewer								X
Procedural Fidelity Checklist	CRF	Therapist			X	X	X	X	X	
Fidelity of training and delivery	Semi-structured interview	Interview								X

ACT, Acceptance and Commitment Therapy.

#### Participant measures

At baseline, we will collect data on name, postcode, NHS number, email address, telephone number, date of birth, gender, marital status, employment, education, menopausal status, year of diagnosis, stage of cancer at diagnosis, tumour type, breast cancer treatment received, comorbidities, AET regimen, supportive therapies used following AET prescription, and previous exposure to ACT, Cognitive Behavioural Therapy and mindfulness.

We will obtain participant consent to apply to NHS Digital for participant-level AET prescribing or dispensing data.

The following patient-reported measures will be used.

##### Morisky Medication Adherence Scale (MMAS-8)^82^

An eight-item, patient report measure for assessing medication adherence. It provides an overall adherence score, as well as a score for intentional and non-intentional non-adherence.

##### Voils DOSE-non-adherence measure—extent scale^83^

A three-item patient report scale to assess the extent of medication adherence.

##### European Organisation for Research and Treatment of Cancer (EORTC) QLQ-C30^84^

A 30-item, patient report tool to assess health-related quality of life in cancer patients.

##### EORTC QLQ-BR45^85^

A 45-item self-report measure for assessing quality of life in patients with breast cancer.

##### EORTC-IL133

Two items assessing vaginal discharge and abnormal vaginal bleeding, taken from the EORTC item library.

##### EQ-5D-5L^86^

A five-item, self-assessed, health-related quality of life questionnaire.

##### Multidimensional psychological flexibility inventory-short form^87^

A 24-item self-report questionnaire assessing psychological flexibility and inflexibility.

##### Beliefs about Medication Questionnaire (BMQ-AET)^88^

The BMQ-AET is a modified version of the BMQ to increase its relevance to women taking AET. It is a 10-item patient report measure which assesses specific medication beliefs.

##### Self-Report Behavioural Automaticity Index (SRBAI)^89^

A four-item self-report tool that captures habitual behaviour patterns, specifically with regard to automaticity of the behaviour.

##### Depression Anxiety Stress Scales (DASS)−21^90^

A 21-item self-report measure of negative emotional states.

##### McGill quality of life-revised^91^

A 14-item, patient-report tool designed to measure physical well-being, physical and psychological symptoms, existential well-being and support, as well as overall quality of life of people with life-threatening illness.

##### UK cancer cost questionnaire^92^

An adapted version of the UK cancer cost questionnaire. The questionnaire assesses services and medications participants have used while participating in the trial.

#### Site measures

##### Usual care assessment

Each site will record what services are offered as part of usual care.

#### Health economic assessments

##### Intervention costs

We will estimate the cost of developing each intervention component. We will use NHS Reference Costs and Personal Social Sciences Research Unit costs to estimate delivery costs for the ACT component.

#### Process evaluation measures

##### Adherence to intervention components

To assess fidelity of receipt of the intervention components, participants will be asked whether they received each of the intervention components they were randomised to, and how much of it they read. Participants who received the ACT component will additionally be asked about how much of the home practice tasks they completed, and how many of the audio files they listened to.

##### Acceptability Questionnaire (AQ)^93^

A five-item self-report measure assessing acceptability. Participants randomised to the SMS component will be asked an additional item about acceptability of the frequency of the SMS messages. Participants randomised to the ACT component will be asked an additional 15 items about the acceptability of elements of ACT sessions. Participants will be provided with an open text question about acceptability. A single item will additionally ask about overall trial acceptability.

##### Study Participant Feedback Questionnaire (SPFQ)^94^

A modified version of the SPFQ assessing participants’ experience during the trial will include two, three and two items at baseline, 2-month and 4-month follow-ups, respectively.

##### ACT Fidelity Measure (ACT-FM) (therapist subscale)^95^

A seven-item subscale from a measure of fidelity to the principles of ACT will be scored by an expert in ACT while reviewing therapy recordings. This subscale includes items measuring prescribed (four items) and proscribed (three items) therapist behaviours.

##### Procedural fidelity checklist

A checklist to assess fidelity of delivery of the ACT component recorded by the therapist at the end of each session to self-rate whether they undertook crucial intervention procedures. It includes four to eight items, depending on the session. Additional items will be used for therapists to record participant engagement with the module materials.

##### Research nurse questionnaire

A nine-item bespoke questionnaire to report RN experiences of recruiting to a fractional factorial trial. Demographic and role information will also be collected for the RNs.

### Process evaluation

#### Fidelity of design

Two independent coders will identify and record which BCTs from the BCT Taxonomy v1 are present in each intervention component.^48^

#### Fidelity of training

Each therapist’s first ACT session will be assessed for competency using the ACT-FM by a clinically trained member of the research team (CDG).

#### Fidelity of delivery

We will collect SMS receipt data, record the number of information leaflets and website details sent to participants, use independently rated competency scores on the ACT-FM therapist stance subscale and report scores on the procedural fidelity checklist.

#### Fidelity of receipt

We will use self-reported data on receipt and reading of the materials within an intervention component, SMS opt outs, website tracking data, therapist-reported attendance and engagement with ACT session and independently assessed engagement with ACT session.

#### Participant and therapist interviews

Semistructured interviews with participants will investigate the acceptability, and fidelity of receipt and enactment of the intervention components. ACT therapists will be interviewed to gain an insight into their experiences of intervention delivery, and the acceptability of the ACT component. Interviews lasting approximately 1 hour will be conducted via video conferencing software or telephone.

### Sample size

The primary objective is to estimate rates of eligibility, recruitment, retention and component adherence to inform the future trial. Allowing for 80% retention (the average across eight studies^20^), 80 participants (n=10 per condition) will be randomised. This is sufficient to inform the sample size of the optimisation trial assuming adherence data are pooled across conditions.^96^

### Patient and public involvement

A user group of five breast cancer survivors have supported the development of the intervention components and trial protocol. Two representatives are members of the trial management group.

### Independent monitoring

The Trial Steering Committee (TSC), with an independent Chair, will be responsible for trial oversight. The TSC will be provided with reports prepared by the CTRU based on an agreed TSC Charter and containing the information agreed in the trial monitoring plan. It includes an Independent Chair, at least two other independent members and two patient representatives. The Chief Investigator and other members of the TMG may attend the TSC meetings and report progress. A separate data monitoring committee was considered by the TSC to be unnecessary. The TSC will adopt a safety function.

### Data management

Data collection forms transferred to or from the CTRU will be coded with a study number (made up of the recruitment site code and the participant’s unique sequential trial number), the participant’s initials and date of birth. Study data will be held securely on paper and electronically at the University of Leeds CTRU, and appropriate processes put in place for the transfer, storage, restricted access and disposal of personal information. Relevant standard operating procedures, guidelines and work instructions in relation to data management, processing and analysis of data will be followed.

### Analysis

A detailed statistical analysis plan will be written and signed off before analysis is undertaken. The analysis will focus on descriptive statistics and CI estimation rather than formal hypothesis testing. All analyses will be undertaken on the intention-to-treat population, with all participants included in the analysis according to their randomised allocation, and regardless of non-adherence to the intervention components or withdrawal from the trial. Final analysis will be conducted once all available outcome data are received. Outcome measures will be scored according to relevant scoring manuals. Predefined progression criteria will be used to judge the feasibility of progressing to the optimisation trial (table 5). Proof of principle will be explored via investigation of between-group change in outcomes. Point estimates and 95% CIs will be presented for the main effects and interaction effects for adherence and quality of life outcomes. Point estimates and 95% CIs for process variables will be presented for the intervention components in which a change is hypothesised. Analysis will adjust for the stratification factor.

**Table 5:** Progression criteria for deciding whether or not to proceed to the optimisation trial

	Green	Amber	Red
Eligible patients consent rate	≥30%	≥10%	<10%
Component adherence			
75% of SMS messages received with no opt out	≥50%	≥20%	<20%
Read ‘at least some’ of the information leaflet	≥50%	≥20%	<20%
Completed 2/4 ACT modules	≥50%	≥20%	<20%
Registered and logged onto website at least once	≥50%	≥20%	<20%
Availability of adherence measures with ≥75% complete data	≥2	≥1	0

Green (go): optimisation phase is feasible with no changes to design or procedures; amber (modify): optimisation phase is feasible following minor enhancement of procedures; red (stop): optimisation phase is not feasible.

ACT, Acceptance and Commitment Therapy; SMS, short message service.

A rapid evaluation approach will be used for the qualitative interviews.^97^ Rapid Research Evaluation and Appraisal Lab (RREAL) sheets, which are summary tables collected after an interview, will be created for each individual participant and collated into higher level summary sheets.^98^ Regular team meetings will discuss emerging findings, changes to the interview schedule and data saturation. The interviews will be recorded. The RREAL sheets will be used to guide analysis, and illustrative quotes used to support themes.

### Recruitment status

The trial opened for recruitment on 20 May 2022, and closed to recruitment on 16 December 2022.

### Ethics and dissemination

#### Ethical approval and data

The trial has been approved by Wales Research Authority Research Ethics Committee 3 (21/WA/0322). It is sponsored by the University of Leeds (governance-ethics@leeds.ac.uk). Amendments to the protocol will be submitted to the ethics committee, and if approved, communicated to research sites. Trial findings will be disseminated through peer-reviewed publications. At the end of the trial, all data held by the CTRU and all trial data will then be securely archived at the University of Leeds for a minimum of 5 years. This paper is a summary of protocol V.3.0 (18 August 2022), available on request from the corresponding author.

#### Dissemination

Results will be presented at scientific meetings and published in international peer-reviewed journals. Authorship decisions will be guided by the International Committee of Medical Journal Editors criteria. Summaries will be provided to participants and the trial funder.

#### Safety

We expect episodes of acute illness, infection, new medical problems and deterioration of existing medical problems will occur and could result in prolonged hospitalisation, hospital readmission, significant or permanent disability or incapacity or death. In recognition of this, events fulfilling the definition of a serious adverse event (SAE) will not be reported unless the event resulted from administration of any research procedure, and fulfils the definition of a related and unexpected serious adverse event (RUSAE). Reports of physical self-harm will also be considered SAE and assessed for relatedness and unexpectedness. We might anticipate the following adverse events related to the intervention; low mood (including suicidal thoughts and plans), fatigue, anxiety and/or psychological distress. Adverse events relating to these factors will not be reported as a RUSAE.

Safety will be monitored through observed increases in ‘extremely severe’ DASS-21 scores for anxiety, stress and depression, a planned RN phone call at 4 months, ad-hoc RN phone calls if a participant wishes to disclose a mental health crisis or referral, disclosures within the semistructured interviews and ACT sessions. Routinely collected hospital data will be used to record deaths, hospitalisations and mental health crisis referrals.

#### Access to data

Data will only be shared for participants who have given consent to use of their data for secondary research, and will only be made available in such a way that recipients cannot identify individuals by any reasonable likely means. We will only share data for projects that have ethical approval granted, are clearly in the public interest and compatible with the original purpose of the data processing. Requests to access trial data should be made to CTRU-DataAccess@leeds.ac.uk in the first instance.

## Supplementary Material

Reviewer comments

Author's manuscript
