# Supplementary material for: Refining and optimising a behavioural intervention to support endocrine therapy adherence (ROSETA) in UK women with breast cancer: protocol for a pilot fractional factorial trial
Source: BMJ Open. 2023 Feb 3;13(2):e069971. doi: 10.1136/bmjopen-2022-069971 (PMC9900066; doi:10.1136/bmjopen-2022-069971)
Supplement: Supplementary data [file bmjopen-2022-069971supp003.pdf]

The TIDieR (Template for Intervention Description and Replication) Checklist*:

Information to include when describing an intervention and the location of the information

Nº	What	Details
1	**Name**	Refining and Optimising a behavioural intervention to support endocrine therapy adherence: The ROSETA Pilot Trial.
2	**Why: Rationale, theory, goal**	Adjuvant hormone therapies are prescribed at the end of hospital-based breast cancer treatment in order to prevent recurrences and all-cause mortality. However adherence to these medications is often poor, due to multiple factors, including forgetting, beliefs about medications, intolerable side effects and psychological distress. This time during the cancer journey is also particularly challenging, as women are transitioning from 'patient' to 'survivor'. They also report a lack of support during this time, post hospital discharge.<br><br>Previous adherence interventions have tended to consist of solely educational based interventions, that are not grounded in theory, and did not target the factors commonly associated with medication adherence. Given the wide range of barriers to adherence in this population, it is perhaps unsurprising that previous interventions have shown limited effectiveness. An alternative strategy is to design a complex intervention, with multiple components that can target a range of factors that have been highlighted as barriers to adherence.<br><br>*Memory and forgetting*<br><br>Mobile phone-based interventions are well suited to tackle forgetfulness as a barrier to adherence, through reminders and promotion of habit formation. SMS messages have been shown to be effective in improving medication adherence in other chronic illnesses but have not been widely tested in cancer patients.<br><br>*Medication schemas*<br><br>Accurate information about the necessity and risks of AET has the potential to increase women's perceptions of their need for AET, and to reduce unfounded concerns about the medication. In addition, women with breast cancer have stated that they would

		like more accurate information about AET to overcome unfounded concerns.<br><br>*Psychological Flexibility*<br><br>Acceptance and Commitment Therapy (ACT) has been shown to improve outcomes in those living with chronic illness, chronic pain, and cancer. ACT aims to increase a participant's awareness of their personal values, and to undertake more of the behaviours that support these values – a process that often involves developing a willingness to have painful thoughts and feelings (such as medication side-effects). ACT targets psychological flexibility, which can improve functioning during objectively difficult circumstances, and can often reduce psychological distress as a by-product.<br><br>*Living with Side effects*<br><br>One of the most commonly cited barriers to AET adherence is the impact of side effects, and the lack of support for management of these is commonly cited. There are a number of strategies for these side effects that have the potential to be effective in alleviating symptoms. However, these are typically not presented in a patient-friendly manner.<br><br>Given the above, we have co-designed four intervention components for women with breast cancer who have been prescribed adjuvant endocrine therapies; SMS reminder messages to target forgetfulness, an information leaflet to promote formation of accurate beliefs, ACT therapy sessions to increase psychological flexibility, and a side-effect management website to support living with side effects. The aim of the intervention components are to support medication adherence to hormone therapy. Participants will be randomised to receive none, or a combination of one or more of these four components.
**3**	**What Materials**	*Participants randomised to receive SMS component*<br><br>Participants received 43 SMS messages over four months. This included three opening messages, one closing message, 36 messages related to behaviour change techniques aiming to

		promote habit formation, and 3 messages (sent after 1, 2 and 3 months) as a reminder that participants can stop any further SMS messages being sent by emailing the ROSETA team. The content of the SMS messages was co-developed with experts in behaviour change and/or medication adherence, and women who have experienced breast cancer.<br><br>*Participants randomised to receive information leaflet*<br><br>Participants received an information leaflet containing detailed information about AET. This included information about how the medication works (with diagrams to supplement), information about the benefits and side effects of AET, answers to common concerns that women have, and quotes from women with experience of taking AET.<br><br>*ACT*<br><br>*Participants randomised to ACT sessions*<br><br>Participants were emailed a participant manual consisting of information about the ACT skill and home practice tasks, in addition to corresponding audio files to assist with the home practice tasks. Each of the four modules focused on a different ACT-based skill:<br>• Module 1: Mindfulness and unhooking<br>• Module 2: Following your values<br>• Module 3: Taking an observer perspective<br>• Module 4: Recap, reflection, and staying committed<br><br>*Therapists delivering ACT sessions*<br><br>Therapists delivering the intervention received two half days of bespoke training delivered by clinical psychologists with ACT experience. Alongside this, they received a training manual, with information about ACT generally, and specific session plans for the intervention sessions.<br><br>*Participants randomised to receive access to side-effect website*

		Participants allocated to receive the website will receive access to a bespoke website containing information and strategies for self-management of side effects, and signposting to further sources of support.
**4**	**What Procedures**	***Intervention Delivery***<br><br>*Participants randomised to receive SMS component*<br><br>Participants received 43 SMS messages over four months. This included three opening messages, one closing message, 36 messages related to behaviour change techniques aiming to promote habit formation, and 3 messages (sent after 1, 2 and 3 months) as a reminder that participants can stop any further SMS messages being sent by emailing the ROSETA team. The content of the SMS messages was co-developed with experts in behaviour change and/or medication adherence, and women who have experienced breast cancer.<br><br>The 36 messages relating to behaviour change techniques were sent on the following schedule:<br><br>– Daily messages for 2 weeks<br>– Two messages per week for 8 weeks<br>– Weekly messages for 6 weeks<br><br>*Participants randomised to receive information leaflet*<br><br>Participants were sent the information leaflet by email immediately after randomisation.<br><br>*Participants randomised to receive ACT*<br><br>4x guided self-help modules consisting of information about ACT, home practice exercises and corresponding audio files<br><br>1x 15-minute individual introductory session with a psychologist<br><br>3 x 25 minute individual support sessions with a psychologist to discuss the module completed over the past week, their experiences of the home practice exercises, and to allow discussion and problem solving of any difficulties that arose.<br><br>1x 15-minute closing session with a psychologist

		Participants randomised to receive access to side-effect website<br><br>Participants were given login details of the website immediately after randomisation.<br><br>*Clinician Training*<br><br>(See section 5, below, for information on this)<br><br>*Evaluation of the Clinician Training*<br><br>Recordings of each therapists first session were reviewed by Dr Graham, using the ACT-FM therapist stance subscale.<br><br>Clinician fidelity was evaluated using the ACT-FM therapist stance subscale completed by an external rater. 10% of recorded sessions were evaluated.<br><br>Clinician fidelity to intervention procedures involved clinician self-rating using a procedural fidelity checklist.<br><br>*Evaluation of the Intervention Components*<br><br>Adherence, quality of life/ symptom burden, psychological distress, psychological flexibility, habitual behaviour of medication taking and medication beliefs were all measured pre and post intervention. Acceptability of each intervention component was assessed. Self reported engagement of intervention components was monitored. SMS delivery, and website use was tracked.<br><br>*Support activities*<br><br>Recruitment and consent of participants
**5**	**Who provided**	*SMS messages*<br><br>The CTRU sent all SMS messages to participants.<br><br>*Information Leaflet*<br><br>The information leaflet was sent to participants by the site.

		ACT<br><br>The therapists who delivered the intervention underwent training in delivering Acceptance and Commitment Therapy. The training was delivered by Dr Chris Graham (CG), who has expertise in ACT applied to chronic disease. Training included teaching about ACT and practice of intervention-specific therapy methods. This course consisted of two half days of training.<br><br>Each site's therapists had a varied background that may or may not have included previous ACT training prior to our delivered training programme. However, all session leads were Health and Care Professional Council (HCPC) or UKCP registered practitioner psychologists or Psychotherapists (Clinical, Health or Counselling Psychologist or Psychotherapists) who worked with breast cancer patients in a hospital setting.<br><br>*Website*<br><br>Access to the bespoke website was provided by the site.
**6**	**How: mechanisms of delivery**	*Participants randomised to receive SMS component*<br><br>SMS messages were sent in an automated fashion by the CTRU to the participants mobile phone based on the following schedule:<br><br>– Daily messages for 2 weeks<br>– Two messages per week for 8 weeks<br>– Weekly messages for 6 weeks<br><br>In addition one message was sent after months 1, 2 and 3 as a reminder that participants can stop any further SMS messages being sent by emailing the ROSETA team.<br><br>*Participants randomised to receive information leaflet*<br><br>Participants were sent the information leaflet electronically immediately after randomisation and were able to read this as they wished.

		Participants randomised to receive ACT<br><br>The individual sessions (5 in total) were delivered via phone or video conferencing.<br><br>The participant manual containing information about each module, home practice tasks, and audio files were emailed to each participant by the therapist following each session.<br><br>*Participants randomised to receive access to side-effect website*<br><br>Participants were given a login to the website immediately after randomisation and were able to use this as they wished.
**7**	**Where: location of delivery**	*SMS Messages*<br><br>Not applicable.<br><br>*Information Leaflet*<br><br>Not applicable.<br><br>*ACT*<br><br>All sessions were delivered remotely via phone or videoconferencing.<br><br>*Website*<br><br>Not applicable
**8**	**When and how much**	*Participants randomised to receive SMS component*<br><br>SMS messages were sent by the CTRU based on the following schedule:<br><br>– Daily messages for 2 weeks<br>– Two messages per week for 8 weeks<br>– Weekly messages for 6 weeks<br><br>In addition three opening messages, one closing message and, one message after months 1,2 and 3 were sent reminding participants

		that they could stop any further SMS messages being sent via emailing the ROSETA team.<br><br>*Participants randomised to receive information leaflet*<br><br>Participants were sent the information leaflet electronically immediately after randomisation and were able to read this as they wished.<br><br>*Participants randomised to receive ACT*<br><br>The introductory session lasted 15 minutes, three subsequent sessions lasted 25 minutes, and the final closing session lasted 15 minutes. Participants had their first session within four weeks of randomisation. The therapy sessions were held weekly.<br><br>*Participants randomised to receive access to side-effect website*<br><br>Participants were given a login to the website immediately after randomisation and were able to use this as they wished.
**9**	**Tailoring**	*SMS*<br><br>The same SMS messages were sent in the same order to each participant.<br><br>*Information Leaflet*<br><br>The same information leaflet was sent to each participant.<br><br>*ACT*<br><br>Although there is a set session plan to follow, detailing specific exercises and tasks for each session, the therapy itself is quite flexible. As such, the deliverer may adapt the content to ensure it's relevant to each participant (e.g. through discussing specific individuals' values, goals, and behaviours).

		Website<br><br>The website was the same for each participant.
**10***	**Modifications**	*<To be completed post study completion>*
**11**	**How well (planned)**	*SMS*<br><br>Successful delivery and receipt of the SMS messages will be recorded by the CTRU, alongside the number of SMS messages that were unable to be delivered. Participants will answer a single item asking whether they received the SMS messages, and another item asking how many of the SMS messages they read. Semi-structured interviews were conducted to understand fidelity of receipt and enactment of the messages.<br><br>*Information Leaflet*<br><br>The number of information leaflets sent out to participants was recorded. This will be recorded by the site when each information leaflet is sent out.<br><br>Participants were asked to self-report whether they received the information leaflet, and how much of the information leaflet they read. Semi-structured interviews were conducted with participants to understand the fidelity of receipt and enactment of the information leaflet.<br><br>*ACT*<br><br>Clinician fidelity to competently deliver the intervention in line with ACT was assessed by an external rater with a background in ACT. They completed the ACT-FM therapist stance subscale checklist whilst listening to the audio recording of 10% of sessions. A score of >4 on ACT consistent behaviours and <5 on ACT inconsistent behaviours is considered competent.<br><br>Additionally, an intervention specific metric of “Procedural Fidelity” was included, which measures other aspects of the intervention that are important for treatment fidelity but are not ACT-specific (e.g. reflecting on home practice tasks, sending module content etc). Therapists will complete the procedural fidelity checklist following each session. A percentage score is created for each session by dividing the score achieved by the

		maximum possible score achievable within that session and multiplying by 100.<br><br>Fidelity of ACT training was monitored through Dr Graham assessing the recording of each therapists first ACT session, and rating competency based on the ACT-FM therapist stance subscale. A score of >4 on ACT consistent behaviours and <5 on ACT inconsistent behaviours is considered competent. Semi-structured interviews with psychologists assessed the fidelity of training and delivery of the ACT component.<br><br>Participant fidelity to the ACT component was monitored by recording the number of sessions attended, missed and cancelled. The therapist additionally reported how much of the module materials the participant had read and engaged with (participant manual, audio files and home practice tasks). Participants self-reported receipt of the module content, self-reported engagement with the participant manual, audio files and home practice tasks. Semi-structured interviews additionally assessed fidelity of receipt and enactment.<br><br>Additionally 10% of recorded sessions were evaluated by an independent reviewer in order to review and assess the recording of a participants engagement in home work tasks by the therapist.<br><br>*Website*<br><br>Website data was tracked for each participant, including number of logins, time spent on pages, videos watched and clicked links. Participants were asked a single item about their engagement with the website. Fidelity of receipt and enactment were additionally assessed through semi-structured interviews.
**12***	**How well (actual)**	*<To be completed post study>*
