# Supplementary material for: Refining and optimising a behavioural intervention to support endocrine therapy adherence (ROSETA) in UK women with breast cancer: protocol for a pilot fractional factorial trial
Source: BMJ Open. 2023 Feb 3;13(2):e069971. doi: 10.1136/bmjopen-2022-069971 (PMC9900066; doi:10.1136/bmjopen-2022-069971)
Supplement: Supplementary data [file bmjopen-2022-069971supp004.pdf]

Delete this line, then print first page on Trust- headed paper

ROSETA

Refining and Optimising a behavioural intervention to Support Endocrine Therapy Adherence

Participant ID: [CTRU Insert]	Initials:
Date of Birth:	NHS Number:
ISRCTN: <<INSERT>>	Principal Investigator:

PARTICIPANT CONSENT FORM

	*Please **initial** each box below*
1. I have read and understand the information sheet dated <<INSERT DATE>> (version X.0) for the above study and have had the opportunity to ask questions.	
2. I understand taking part in this study is voluntary and I am free to withdraw at any time without it affecting my medical care or legal rights.	
3. I understand that if I withdraw from the study, the data collected from me up until that point will still be retained and analysed.	
4. I understand that relevant sections of my medical records and/or study data may be looked at by responsible individuals from the research team, the sponsor (University of Leeds), Leeds Clinical Trials Research Unit, relevant third parties or from regulatory authorities where it is relevant to my participation. I give permission for these individuals to access my records.	
5. I understand that if during this study my clinical care team determine that I have lost my ability to make my own decisions, I will be withdrawn from the study and no further study information will be collected. I agree that data collected up until this point will remain on file and will be included in the analysis.	
6. I consent to the secure transfer, storage and use of paper and electronic personal information, for the purposes of this study to the Leeds Clinical Trials Research Unit, or relevant third parties. I understand that any information that could identify me will be kept strictly confidential and that no personal information will be included in the study report or other publication.	
7. I give consent for my personal details (which will include my full name, date of birth, gender, and NHS number) to be shared with NHS Digital for the purpose of the research team obtaining	

Smith SG, *et al. BMJ Open* 2023; 13:e069971. doi: 10.1136/bmjopen-2022-069971

Delete this line, then print first page on Trust- headed paper

prescribing and dispensing data for my adjuvant endocrine therapy medication.		
8. If randomised to receive Acceptance and Commitment Therapy, I give consent for the sessions to be audio recorded.		
9. If randomised to have access to the website, I consent for my use of the website to be tracked, which will include capturing data on pages visited, links downloaded and number of visits to the website.		
10. I agree to a copy of this consent form being sent to Leeds CTRU.		
11. I agree to my General Practitioner being notified of my participation in this study.		
12. I understand that my confidentiality will be kept unless, during the course of the study, the researcher/clinical team, has reason to believe that I am at risk of harming myself or others and this may involve a member of the research/clinical team contacting me.		
13. I agree that record-level information (data related to a single individual) collected about me may be used to support other research in the future but that I will not be directly identified. Data may be shared anonymously with other researchers.		
14. I will provide the research team with an email address so I can receive log in details for the online completion of questionnaires. I agree that my email address can also be used to send me information relevant to the interventions I may be randomised to in the study.		
15. I will provide the research team with a mobile telephone number, and understand that I may receive SMS messages to prompt me when follow up questionnaires are being sent and need to be completed. I agree that if I am randomised to receive the SMS intervention, I will also receive messages reminding to me to take my AET medication. I understand that to do so my phone number will be shared with a SMS service provider.		
16. I agree to take part in the study.		

Optional results consent

	*Please **initial** relevant box below*	
17. I would like to be contacted about the results of this study using the contact details I have provided.	Yes	
	No	

**Optional interview consent**	*Please **initial** relevant box below*	
18. I am happy to be contacted by a member of the research team about taking part in the follow up semi-structured interview and I	Yes	

Participant_Consent Form_v2.0_03.02.2022
ISRCTN: <<INSERT>>/REC: <<INSERT>>/IRAS: 302050

Delete this line, then print first page on Trust- headed paper

understand taking part in this is separate to the intervention and is not mandatory.	No	

Face-To-Face

Participant- Please sign and date:

Signature……………………………………………………………………………………

Name (block capitals)………………………………………………….…………………

Date…………………………………………………….…………………………………

Please now return this form to the Research Nurse

To be completed on receipt by the Research Nurse;

Research Nurse:

I have explained the study to the above participant and she has indicated her willingness to participate. I have placed a copy of this consent form in her medical notes.

Signature……………………………………..……………………………………

Name (block capitals)………………………………………………………………

Date…………………………………………………….…………………………………

Smith SG, *et al. BMJ Open* 2023; 13:e069971. doi: 10.1136/bmjopen-2022-069971

Delete this line, then print first page on Trust- headed paper

Telephone Consent

Research Nurse:

I have explained the study and read the statements to the above named participant. She has indictated her willingness to participate and agreed to each compulsory statement, so I have initialled and signed on her behalf.

Signature…………………………………...……………………………………

Name (block capitals)…………………………………………….…………………

Date……………………………………………………….……………………………

Note for Research Nurse (Original copy to be sent to the CTRU; 1 copy returned to the participant; 1 copy to be stored in Investigator Site File, 1 copy for treating clinician (where appropriate))

Smith SG, *et al. BMJ Open* 2023; 13:e069971. doi: 10.1136/bmjopen-2022-069971
