# Supplementary material for: Refining and optimising a behavioural intervention to support endocrine therapy adherence (ROSETA) in UK women with breast cancer: protocol for a pilot fractional factorial trial
Source: BMJ Open. 2023 Feb 3;13(2):e069971. doi: 10.1136/bmjopen-2022-069971 (PMC9900066; doi:10.1136/bmjopen-2022-069971)
Supplement: Author's manuscript [file bmjopen-2022-069971.draft_revisions.pdf]

BMJ Open is committed to open peer review. As part of this commitment we make the peer review history of every article we publish publicly available.

When an article is published we post the peer reviewers' comments and the authors' responses online. We also post the versions of the paper that were used during peer review. These are the versions that the peer review comments apply to.

The versions of the paper that follow are the versions that were submitted during the peer review process. They are not the versions of record or the final published versions. They should not be cited or distributed as the published version of this manuscript.

BMJ Open is an open access journal and the full, final, typeset and author-corrected version of record of the manuscript is available on our site with no access controls, subscription charges or pay-per-view fees (http://bmjopen.bmj.com).

If you have any questions on BMJ Open's open peer review process please email info.bmjopen@bmj.com

BMJ Open

Refining and Optimising a behavioural intervention to Support Endocrine Therapy Adherence (ROSETA) in women with breast cancer: protocol for a pilot fractional factorial trial

Journal:	*BMJ Open*
Manuscript ID	bmjopen-2022-069971
Article Type:	Protocol
Date Submitted by the Author:	13-Dec-2022

Complete List of Authors:	Smith, Samuel; University of Leeds Leeds Institute of Health Sciences<br>Green, Sophie M. C.; Leeds Institute of Health Science University of Leeds<br>Ellison, Rachel; University of Leeds, Clinical Trials Research Unit<br>Foy, Robbie; University of Leeds, Leeds Institute of Health Sciences<br>Graham, Christopher D.; Queens University Belfast<br>Mason, Ellen; University of Leeds Leeds Institute of Clinical Trials Research<br>French, David; University of Manchester, School of Psychological Sciences<br>Hall, Louise; University of Leeds, Institute of Health Sciences<br>Wilkes, Hollie; University of Leeds, Clinical Trials Research Unit<br>McNaught, Emma; University of Leeds, Clinical Trials Research Unit<br>Raine, Erin; Leeds Institute of Health Science University of Leeds<br>Walwyn, Rebecca; University of Leeds, Clinical Trials Research Unit<br>Howdon, Daniel; University of Leeds Leeds Institute of Health Sciences, Academic Unit of Health Economics<br>Clark, Jane; Leeds Teaching Hospitals NHS Trust, Department of Clinical and Health Psychology<br>Rousseau, Nikki; University of Leeds, Surgical, Diagnostic and Devices Division<br>Buxton, Jacqueline; University of Leeds<br>Moore, Sally J. L.; University of Leeds<br>Parbutt, Catherine; Leeds Teaching Hospitals NHS Trust<br>Velikova, Galina; University of Leeds, Leeds Institute of Medical Research<br>Farrin, Amanda; University of Leeds, Clinical Trials Research Unit<br>Collinson, Michelle; University of Leeds Clinical Trials Research Unit

Keywords:	Breast tumours < ONCOLOGY, PREVENTIVE MEDICINE, PUBLIC HEALTH

SCHOLARONE™
Manuscripts

Refining and Optimising a behavioural intervention to Support Endocrine Therapy Adherence (ROSETA) in women with breast cancer: protocol for a pilot fractional factorial trial

Samuel G. Smith[1], Sophie M. C. Green[1], Rachel Ellison[2], Robbie Foy[1], Christopher D. Graham[3], Ellen Mason[2], David P. French[4], Louise H. Hall[1], Hollie Wilkes[2], Emma McNaught[2], Erin Raine[1], Rebecca Walwyn[2], Daniel Howdon[1], Jane Clark[5], Nikki Rousseau[2], Jacqueline Buxton[6], Sally J. L. Moore[6], Catherine Parbutt[7], Galina Velikova[8], Amanda Farrin[2], Michelle Collinson[2]

1. Leeds Institute of Health Science, University of Leeds
2. Clinical Trials Research Unit, Leeds Institute of Clinical Trials Research, University of Leeds
3. School of Psychology, Queens University Belfast
4. Division of Psychology and Mental Health, University of Manchester
5. Department of Clinical and Health Psychology, Leeds Teaching Hospitals NHS Trust
6. Independent
7. Medicines Management and Pharmacy Services, Leeds Teaching Hospitals NHS Trust
8. Leeds Institute for Medical Research, University of Leeds

Corresponding author:

Dr Samuel Smith

E: s.smith1@leeds.ac.uk

T: 0113 343 0892

Words (abstract): 297/350

Words (article): 4005/4000

For peer review only - http://bmjopen.bmj.com/site/about/guidelines.xhtml

Abstract

Introduction: Women with breast cancer who do not adhere to adjuvant endocrine therapy (AET) have increased risks of mortality and recurrence. There are multiple barriers to AET adherence, including medication side-effects, beliefs about medication, memory, and psychological distress. We developed four intervention components, each targeting a different barrier. This pilot trial is part of the preparation phase of the Multiphase Optimisation Strategy (MOST), and aims to establish key trial parameters, establish intervention component adherence, establish availability and feasibility of outcome and process data, estimate variability in planned outcome measures, and estimate cost of developing and delivering each intervention component.

Methods and analysis: The four intervention components are: SMS text reminders (target: memory); a written information leaflet (target: medication beliefs); a guided self-help Acceptance and Commitment Therapy programme (target: psychological flexibility to reduce distress); a self-management website (target: side-effect management). To evaluate the feasibility of recruitment, acceptability of the intervention components and the availability of outcome data, we will conduct a multi-site, exploratory pilot trial using a 2^{4-1} fractional factorial design, with a nested process evaluation. We will randomise 80 women with early-stage breast cancer who have been prescribed AET to one of eight experimental conditions. This will determine the combination of intervention components they receive, ranging from zero to four, with all conditions receiving usual care. Key outcomes of interest include medication adherence and quality of life. Progression to the optimisation phase will be based on pre-defined criteria for consent rates, patient adherence to intervention components and availability of medication adherence data.

Ethics and dissemination: The study was reviewed by the Wales Research Authority Research Ethics Committee 3 (21/WA/0322). Written informed consent will be obtained from all patients before randomisation. The results of this trial will be disseminated in a peer-reviewed journal.

Trial registration: ISRCTN: 10487576 (16/12/2021)

Keywords: Medication adherence; breast cancer; Multiphase Optimisation Strategy (MOST); Acceptance and Commitment Therapy; SMS reminders; information leaflet, self-management website, pilot; feasibility; quality of life; factorial

For peer review only - http://bmjopen.bmj.com/site/about/guidelines.xhtml

Strengths and limitations of this study

- This design will estimate the individual and combined effects of multiple intervention components
- This multi-site pilot will recruit women through 3 routes
- Progression to an optimisation trial will be decided using a priori criteria
- A mixed-methods process evaluation will assess intervention acceptability and fidelity
- The pilot will not examine effectiveness of the intervention components

For peer review only

For peer review only - http://bmjopen.bmj.com/site/about/guidelines.xhtml

Introduction

Approximately 55,000 UK women are diagnosed with breast cancer each year, with 11,000 cancer-related deaths [1]. Most breast cancers are oestrogen receptor positive (ER+) tumours. Adjuvant endocrine therapies (AETs) such as selective oestrogen receptor modulators (SERMs e.g. tamoxifen) and aromatase inhibitors (AIs e.g. Letrozole) improve outcomes for women with ER+ tumours. Low adherence to AET is linked with higher rates of recurrence and all-cause mortality [2–9]. A meta-analysis reported 31% to 73% of breast cancer survivors are adherent to AET [10], and stopping AET is most common within 12 months of initiation [11–13]. Unintentional non-adherence, such as forgetting, may be more common than intentionally missing doses [14–16].

An overview of behavioural support programmes for women using AET reported at least 15 ongoing trials targeting AET adherence [17]. Among adequately powered trials of AET interventions there are few positive findings [18–20]. This may be explained by the reliance on interventions involving the provision of written information; which may address some adherence barriers, but is unlikely to address the complex issues underpinning most medication taking behaviours [17,21]. As with many long-term conditions where treatment non-adherence is a problem, intervention programmes may require multiple active components to target a range of key determinants.

Existing AET adherence trials have used single-arm or parallel group randomised designs. While parallel group trials are suitable for definitively evaluating a complex intervention against a suitable comparator, they provide little information on the relative contributions of individual components [22]. Undertaking separate or multi-arm randomised controlled trials (RCTs) to evaluate individual intervention components would be prohibitively expensive and time-consuming, and would not allow estimates of interactions between intervention components. Alternative trial designs are needed to improve our understanding of complex interventions, and increase the efficiency of definitive evaluations.

The Multiphase Optimisation Strategy (MOST) is a framework that includes an optimisation phase between developing and evaluating a complex intervention [22]. Within this optimisation phase, MOST advocates the use of highly efficient experimental designs to provide empirical data on the main effects and interactions of intervention components. This information is used to identify the optimal combination of intervention components to produce the desired outcome, without exceeding key constraints (e.g. cost) [23]. The optimised intervention package can be evaluated in a definitive RCT. The process of optimisation aims to yield more effective, affordable, scalable and efficient

For peer review only - http://bmjopen.bmj.com/site/about/guidelines.xhtml

intervention packages compared with alternative approaches [24,25]. Given the complexity of adherence behaviours, the lack of information available about individual intervention component efficacy, and the need to address an important clinical need efficiently, we used the MOST framework to guide our approach to intervention optimisation.

As part of the preparatory phase of MOST, we used Intervention Mapping to develop a theoretically informed intervention package [17,26]. We identified several modifiable factors associated with AET adherence [27–30], selecting four of the strongest determinants as intervention targets [17]. Four intervention components were developed or adapted to address these targets. The current trial represents the next step in the preparation phase of MOST; examining the feasibility of an optimisation trial of the proposed intervention package in women with early-stage breast cancer. Our objectives are to: i) establish eligibility, recruitment, retention and follow-up rates; ii) establish intervention component adherence; iii) establish availability and feasibility of outcome and process data; iv) estimate variability of planned outcome measure(s); and v) estimate cost of developing and delivering each intervention component.

We will also include a nested process evaluation to further inform the decision of whether to undertake a future optimisation trial, and how the intervention components could be adapted based on intervention fidelity and acceptability, and trial experience. A full protocol of this process evaluation is published elsewhere [31].

Methods and analysis

Design

This is a multi-site, exploratory pilot trial using a 2^{4-1} fractional factorial design with a nested mixed methods process evaluation. We will randomise 80 women with early-stage breast cancer to one of eight experimental conditions, which will determine the intervention components they receive (Table 1). There will be an equal chance of allocation to each group. Each component has two levels (present or absent). All women will receive usual care. Women will be followed-up at 2 and 4-months, and key outcomes include medication adherence (self-report and NHS data) and quality of life. The trial adheres to the SPIRIT recommendations [32], and the CONSORT extension for pilot and feasibility trials [33] (Appendix) and the intervention components are described using the TIDieR checklist [34] (Appendix).

For peer review only - http://bmjopen.bmj.com/site/about/guidelines.xhtml

Table 1. 2^{4-1} fractional factorial design for pilot trial

Condition	Usual Care	Text reminders	Information leaflet	Acceptance & Commitment Therapy	Side-effect website	Randomisation sample size
**1**	Yes	Yes	Yes	Yes	Yes	10
**2**	Yes	Yes	Yes	No	No	10
**3**	Yes	Yes	No	Yes	No	10
**4**	Yes	Yes	No	No	Yes	10
**5**	Yes	No	Yes	Yes	No	10
**6**	Yes	No	Yes	No	Yes	10
**7**	Yes	No	No	Yes	Yes	10
**8**	Yes	No	No	No	No	10

For peer review only

Setting

Participant identification and recruitment will occur at five NHS hospitals, detailed in the ISRCTN registry (ISRCTN10487576). Sites with existing or planned implementation of interventions designed to improve adherence to AET will be excluded. The site must have access to a Health and Care Professional Council (HCPC) registered practitioner psychologist (Clinical, Health or Counselling Psychologist) or a UK Council for Psychotherapy (UKCP) registered psychotherapist. The site must have access to video conferencing software or a telephone to deliver the ACT sessions.

Participants

Adult women using tamoxifen, raloxifene, anastrozole, letrozole, or exemestane as AET for early-stage (1 to 3a) breast cancer are eligible (Table 2).

Table 2. Eligibility criteria for participation in the ROSETA pilot trial

Inclusion Criteria	Exclusion Criteria
1. An informed consent form (signed and dated)<br>2. Capacity to provide informed consent<br>3. Women with early stage (1 to 3a) breast cancer according to the TNM / American Joint Committee on Cancer (AJCC) staging system.<br>*Note. Women being treated for a second primary breast cancer or a breast cancer local recurrence are eligible for the study, providing the most recent cancer is being treated with adjuvant endocrine therapy, and they meet all eligibility criteria. Women with bilateral breast cancer are permitted, providing at least one breast is affected by hormone receptor-positive disease'*<br>4. Aged ≥18 years at time of screening for ROSETA's pilot study	1. Stopped taking adjuvant hormone therapy if it is clinically contraindicated according to clinical recommendation<br>2. Women with Metastatic breast cancer<br>3. Currently or recently (last 6 months) involved in a similar research study where medication adherence is a primary outcome*<br>4. Currently attending psychotherapy/psycho-oncology/psychology/counselling services, for any clinical reason*<br>5. Need for treatment for a severe mental health disorder or crisis, which is likely to interfere with participation (e.g., active psychosis, bipolar disorder, significant issues with addiction or self-harm or expressing

For peer review only - http://bmjopen.bmj.com/site/about/guidelines.xhtml

5. Have sufficient proficiency in English to be able to adhere to all intervention components and data collection required<br>6. Treated with curative intent<br>7. Completed their hospital-based treatment (e.g., surgery, radiotherapy and/or chemotherapy) for the current breast cancer within the last 12 months.<br>*Note. Women are still eligible for the study if they are being treated with monoclonal antibody-based therapy such as trastuzumab, kadcyla, pertuzumab, and phesgo*<br>8. Currently prescribed oral adjuvant Hormone Therapy (tamoxifen, raloxifene, anastrozole, letrozole, exemestane)<br>9. The participant is willing to complete the study questionnaires*<br>10. The participant is willing to be audio recorded during the therapy sessions*<br>11. The participant is willing and able to attend all ACT sessions either via video conference or telephone*<br>12. The participant is willing and able to complete home practice tasks*<br>13. Access to a mobile phone to receive SMS messages*<br>14. Willing to receive frequent SMS messages*<br>15. Access to a computer or smart device that can access the internet*	active suicidal ideation with active plans and intent*)<br>*Note, if concerned about the possible presence of risk of suicidal ideation with active plans and intent, then this can be assessed with the following questions, with patients ineligible if they answer 'yes' to 5c.*<br><br>*Recently (in the last month):*<br>*a. Have you had any thoughts about ending your life?*<br>*b. (if yes) Have you thought about how you might go about it?*<br>*c. (if yes) Do you intend to carry out this plan?*<br><br>6. Patients with a scheduled date for breast reconstruction surgery that is within their intervention delivery and follow-up period. *Note: Women planning to have a breast reconstruction but who have not scheduled a date for surgery are permitted*<br><br>7. Auditory problems that would prevent the patient from participating in a telephone or video call, or hearing audio clips*

*Source data for these items will be either partially or completely patient self-report.

Trial Processes

Participants will be identified via three routes. Route 1 involves the research nurse (RN) prospectively screening patient records prior to clinic visits. Route 2 will identify patients who have self-referred to their care team to discuss problems with their medications. Route 3 involves the RN retrospectively searching patient records to identify patients who have completed hospital treatment. All patients should have completed their last hospital treatment within 12 months of randomisation.

Sites will complete a screening form for all identified patients. Anonymised data on age, ethnicity, staging, tumour type and whether a patient is randomised will be captured. Patients who are not randomised will have the reason they are ineligible or declined participation recorded.

For peer review only

For peer review only - http://bmjopen.bmj.com/site/about/guidelines.xhtml

For recruitment route 1 a member of the care team will introduce a RN to the patient, and offer the option of receiving trial documents by email. Eligible patients may consent immediately or after further consideration. In route 2, the patient's oncologist will conduct an initial review of eligibility and, where participants are willing, provide their contact details to a RN who will telephone them and email trial documents. Interested patients contact the RN by telephone and consent is taken. For route 3 the RN will post or email the patient an invitation letter and a copy of the patient information sheet. For all routes, a RN will confirm eligibility and record consent (Appendix). Eligibility and consent discussions, and completion of baseline measures may take place in person or remotely.

Authorised individuals at NHS sites will be given access to the Clinical Trials Research Unit (CTRU) online database and will enter site and participant details, and confirm eligibility, and informed consent. After recording consent, the RN will register the participant and a link to the baseline questionnaire will be sent automatically from the CTRU. Once completed, the RN will notify the CTRU that a participant can be randomised, and an authorised member of the CTRU will perform the randomisation. Participants will be randomised to one of eight experimental conditions (Table 1). Stratified permuted block randomisation will ensure experimental conditions are well-balanced for the recruitment route. Participants, therapists, RNs, participants' GPs and CTRU staff (including the Chief Investigator) will not be blinded to the randomised allocation. The RN will notify participants of their allocation.

We will ask participants to complete electronic follow-up questionnaires at 2 and 4-months post-randomisation. Non-responders will receive telephone, email and/or SMS message reminders.

Patients may withdraw from the SMS and ACT intervention components, completing the questionnaires, or from the collection of data from NHS Digital and data processing. The local principal investigator may decide a participant should be withdrawn if they have become unsuitable for the trial. The analysis will use data collected up to the date of withdrawal of consent.

Usual Care

All participants will receive treatment as usual, which will be the standard care offered to women at this stage of their breast cancer treatment. It is likely to differ by recruiting site. Most women will be invited to attend an end of treatment summary meeting with a breast care nurse. The content of treatment as usual programmes and information on hospital-based services accessed will be reported through patient self-report and site-level report. Providers of usual care will not be blinded.

For peer review only - http://bmjopen.bmj.com/site/about/guidelines.xhtml

For peer review only

Intervention components

SMS Intervention Component

Memory problems are common in women with breast cancer [35,36], and forgetting is a cause of non-adherence to AET [14,15,37,38]. Forming medication taking habits can reduce reliance on memory and help sustain behaviour change [39–42]. Mobile phone messaging interventions are a potential cost-effective approach to promote habit formation [43–45]. This has not been widely tested in cancer patients [46].

We co-developed, with behaviour change experts and women affected by breast cancer, an intervention component involving SMS reminders to support habit formation of daily medication taking and associated behaviours (e.g., ordering prescriptions) [47]. The SMS messages are based on six behaviour change techniques theorised to support the development of habitual behaviours [48]. The SMS messages will be sent by CTRU and delivered over four months, commencing up to one week following randomisation. Forty-three SMS messages will be delivered to participants in the same order; three opening messages, daily messages for 2 weeks, two messages per week for 8 weeks, weekly messages for 6 weeks, and one closing message. A message will be sent monthly informing participants they can stop the SMS messages by emailing the trial team.

Medication Beliefs Intervention Component

Women using AET report low perceived need for therapy, while also citing unfounded concerns about the medication, which could impact adherence [28,29,49–52]. Qualitative studies have highlighted a demand for accurate information about AET to overcome unfounded concerns [27–29].

We developed a 6-page patient information leaflet to target AET medication beliefs. The leaflet explains how AET works supplemented by diagrams, information about AET benefits and side effects, answers to common concerns, and quotes and photos of breast cancer survivors. Leaflet content is informed by existing qualitative research, views from our patient and public involvement group, the Necessity Concerns Framework [52] and the Common-Sense Model of Self-regulation [53]. Participants receiving this component will be emailed the leaflet by the NHS site, after randomisation.

Acceptance and Commitment Therapy (ACT) Intervention Component

Approximately half of women with breast cancer experience higher distress than the general population [30,54,55]. Distress is associated with adherence to AET [30,56], and interventions

For peer review only - http://bmjopen.bmj.com/site/about/guidelines.xhtml

targeting this barrier could support medication adherence behaviours. Any prospective intervention would need to target the factors contributing to distress, including fear of recurrence, difficulties 'returning to normal', and distress caused by side-effects [27,57,58].

The ACT component is a guided self-help programme targeting psychological distress, by promoting psychological flexibility [59,60]. We used an adapted version of a guided self-help ACT intervention shown to be effective for reducing distress in people with muscle diseases [61]. It consists of five telephone or video call sessions with a therapist. The modules suggest four ACT-based skills; mindfulness, unhooking, following values and living beyond labels, that aim to enhance well-being and reduce distress. Each module consists of a participant manual containing information about an ACT skill, alongside home practice exercises and audio files.

The participant will see the same therapist for each session. The first session will take place within 4 weeks of randomisation and consists of a 15-minute introduction. The three weekly sessions with the therapist will last 25 minutes each. Within these sessions, the therapist and the patient will discuss the previous week's module, and reflect on the participant's experience of using the skills. Following each session, the therapist will email the patient the next module's materials. One week after the fourth module there will be a closing 15-minute support session. Participants allocated to receive ACT can cease participation in that component by informing the therapist or contacting the trial team.

Self-management Website Intervention Component

AET side effects (e.g. hot flushes and arthralgia [62–67]) reduce quality of life [62,68–72]. Side-effects also likely impact medication adherence [68,73–75], although evidence is mixed [76]. Oncologists perceive side-effects to be a major deterrent to AET adherence [77], which is corroborated by patients [27,78]. Women feel unsupported in managing side-effects [27,57,78], and would like clearer information on self-management strategies [58,79]. We undertook an umbrella review of systematic reviews and clinical guidelines on self-management strategies for common AET side-effects [80]. This review informed the creation of a patient-facing website to support side-effect management in women using AET.

Website creation was also based on suggestions made by patients and healthcare professionals attending a co-development workshop [81]. The website includes videos of patient stories, sign-posting for further information, and information on common AET side-effects. Participants

For peer review only - http://bmjopen.bmj.com/site/about/guidelines.xhtml

randomised to the website component will be sent login details by the NHS site following randomisation.

Measures

Table 3 and Table 4 summarise timing of data collection for each assessment.

Participant Measures

At baseline, we will collect data on name, postcode, NHS number, email address, telephone number, date of birth, gender, marital status, employment, education, menopausal status, year of diagnosis, stage of cancer at diagnosis, tumour type, breast cancer treatment received, co-morbidities, AET regimen, supportive therapies used following AET prescription, and previous exposure to ACT, Cognitive Behavioural Therapy and mindfulness.

We will obtain participant consent to apply to NHS Digital for participant-level AET prescribing or dispensing data.

The following patient-reported measures will be used:

Morisky Medication Adherence Scale (MMAS-8) [82]

An 8-item, patient report measure for assessing medication adherence. It provides an overall adherence score, as well as a score for intentional and non-intentional non-adherence.

Voils DOSE-Nonadherence measure- Extent Scale [83]

A 3-item patient report scale to assess the extent of medication adherence.

European Organisation for Research and Treatment of Cancer (EORTC) QLQ-C30 [84]

A 30-item, patient report tool to assess health-related quality of life in cancer patients.

EORTC QLQ-BR45 [85]

A 45-item self-report measure for assessing quality of life in patients with breast cancer.

EORTC-IL133

Two items assessing vaginal discharge and abnormal vaginal bleeding, taken from the EORTC item library.

For peer review only - http://bmjopen.bmj.com/site/about/guidelines.xhtml

Table 3. Summary of assessments

Assessment	Source	Method of Completion	TIMELINE				
			Screening	Baseline	2 Months	4 Months	End of Trial
**PARTICIPANT**							
Screening	Screening CRF	Research Nurse/Delegate	X				
Contact Details	CRF	Research Nurse/Delegate		X	CTRU to be notified of changes to contact details		
Demographics including comorbidities (Charlson Comorbidity Index)	CRF	Self-completion/Research Nurse/Delegate		X			
Eligibility (including Inclusion and Exclusion Criteria)	CRF	PI/Research Nurse/PIs Delegate		X			
Consent	Consent Form	Self-completion / RN/Delegate over the telephone		X			
Randomisation	CRF/CTRU Online system	Research Nurse/Delegate		X			
Prescribing/Dispensing Data	NHS Digital	PIs Delegate		X	X	X	
MMAS-8	Questionnaire	Self-completion		X	X	X	
Voils DOSE-Nonadherence measure- Extent Scale	Questionnaire	Self-completion		X	X	X	
EORTC QLQ-C30	Questionnaire	Self-completion		X	X	X	
EORTC QLQ-BR45	Questionnaire	Self-completion		X	X	X	
EORTC-IL133	Questionnaire	Self-completion		X	X	X	
EQ-5D-5L	Questionnaire	Self-completion		X	X	X	
Intervention costs	NHS Reference Costs/ PSSRU	PIs Delegate				X	
MPFI (short form)	Questionnaire	Self-completion		X	X	X	
BMQ-AET	Questionnaire	Self-completion		X	X	X	

For peer review only - http://bmjopen.bmj.com/site/about/guidelines.xhtml

SRBAI	Questionnaire	Self-completion		X	X	X	
DASS-21	Questionnaire	Self-completion		X	X	X	
McGill QoL Revised	Questionnaire	Self-completion		X	X	X	
Safety Reporting	CRF	Research Nurse/PI/Delegate			X		
End of Trial Data	CRF	Research Nurse					X
End of Trial Site Data	CRF	Research Nurse					X
SMS Delivery and receipt Data	Online System	Delegate			X	X	
Opt-out of SMS messages	Online system	Delegate			X (collected throughout)		
Information leaflet delivery	Site recorded	Research Nurse	X				
ACT session attendance, number of cancelled/ missed sessions	CRF	Clinician			X (collected throughout)		
Engagement with ACT module materials (participant manual, associated audio files and home practice tasks)	CRF	Clinician			X (collected after each therapy session)		
Engagement with ACT module audio files	Questionnaire	Self-completion				X	
Completion of ACT home practice tasks	Questionnaire	Self-completion				X	
Dates of ACT sessions	CRF	Clinician			X (collected throughout)		
Printing of ACT module booklets	Questionnaire	Self-completion				X	
Delivery of website log in details	Site recorded	Research Nurse		X			
Website usage	Website Online System	Delegate			X	X	
Self-reported receipt of SMS, information leaflet, ACT modules and website	Questionnaire	Self-completion				X	
Self-reported reading of SMS, information leaflet,	Questionnaire	Self-completion				X	

For peer review only

For peer review only - http://bmjopen.bmj.com/site/about/guidelines.xhtml

ACT module participant manuals and website							
Acceptability Questionnaire	Questionnaire	Self-completion				X	
Questionnaire return	Online system	Delegate		X	X	X	
Trial withdrawals	CRF	Delegate	X (collected throughout)				
Study Participant Feedback Questionnaire (SPFQ)	Questionnaire	Self-completion		X	X	X	
Fidelity of receipt and enactment of intervention components	Semi-structured interview	Interview				X	X
Barriers and facilitators to trial participation and response rates	Semi-structured interview	Interview					X
Barriers and facilitators to recruitment	Questionnaire	Research Nurse Self-completion					X
Research Nurse Demographics	CRF	Research Nurse		X			
UK Cancer Costs Questionnaire	Questionnaire	Self-completion			X	X	
Questionnaire reminder preference	Questionnaire	Self-completion				X	

For peer review only

Table 4. Summary of ACT therapist assessments

Assessment	Source	Method of Completion	

			Post-therapist training	Pre-trial delivery	1st session	2nd session	3rd Session	4th Session	5th Session	End of trial
Consent	Consent form	Therapist	X							
Demographics	CRF	Therapist	X							
ACT-FM Competency Assessment (Therapist stance subscale)	Questionnaire	ACT trainer			X					
Number of booster training sessions required	Questionnaire	ACT trainer		X (collected throughout)						
ACT-FM therapist stance subscale	Questionnaire	Independent Expert Reviewer								X
Procedural Fidelity Checklist	CRF	Therapist			X	X	X	X	X	
Fidelity of training and delivery	Semi-structured interview	Interview								X

For peer review only - http://bmjopen.bmj.com/site/about/guidelines.xhtml

EQ-5D-5L [86]

A 5-item, self-assessed, health-related quality of life questionnaire.

Multi-Dimensional Psychological Flexibility Inventory-Short form [87]

A 24-item self-report questionnaire assessing psychological flexibility and inflexibility.

Beliefs about Medication Questionnaire (BMQ-AET) [88]

The BMQ-AET is a modified version of the BMQ to increase its relevance to women taking AET. It is a 10-item patient report measure which assesses specific medication beliefs.

Self-Report Behavioural Automaticity Index (SRBAI) [89]

A 4-item self-report tool that captures habitual behaviour patterns, specifically with regard to automaticity of the behaviour.

Depression Anxiety Stress Scales (DASS)-21 [90]

A 21-item self-report measure of negative emotional states.

McGill Quality of Life-Revised [91]

A 14-item, patient-report tool designed to measure physical well-being, physical and psychological symptoms, existential well-being and support, as well as overall quality of life of people with life-threatening illness.

UK Cancer Cost Questionnaire [92]

An adapted version of the UK cancer cost questionnaire. The questionnaire assesses services and medications participants have used while participating in the trial.

Site measures

Usual Care Assessment

Each site will record what services are offered as part of usual care.

Health economic assessments

Intervention costs

For peer review only - http://bmjopen.bmj.com/site/about/guidelines.xhtml

We will estimate the cost of developing each intervention component. We will use NHS Reference Costs and Personal Social Sciences Research Unit costs to estimate delivery costs for the ACT component.

Process evaluation measures

Adherence to intervention components

To assess fidelity of receipt of the intervention components, participants will be asked whether they received each of the intervention components they were randomised to, and how much of it they read. Participants who received the ACT component will additionally be asked about how much of the home practice tasks they completed, and how many of the audio files they listened to.

Acceptability Questionnaire (AQ) [93]

A 5-item self-report measure assessing acceptability. Participants randomised to the SMS component will be asked an additional item about acceptability of the frequency of the SMS messages. Participants randomised to the ACT component will be asked an additional 15 items about the acceptability of elements of ACT sessions. Participants will be provided with an open text question about acceptability. A single item will additionally ask about overall trial acceptability.

Study Participant Feedback Questionnaire (SPFQ) [94]

A modified version of the SPFQ assessing participants' experience during the trial will include 2, 3, and 2 items at baseline, 2-month and 4-month follow-ups, respectively.

ACT Fidelity Measure (ACT-FM) (therapist subscale) [95]

A 7-item subscale from a measure of fidelity to the principles of ACT will be scored by an expert in ACT while reviewing therapy recordings. This subscale includes items measuring pre- scribed (4 items) and pro-scribed (3 items) therapist behaviours.

Procedural Fidelity Checklist

A checklist to assess fidelity of delivery of the ACT component recorded by the therapist at the end of each session to self-rate whether they undertook crucial intervention procedures. It includes 4 to 8 items, depending on the session. Additional items will be used for therapists to record participant engagement with the module materials.

Research Nurse Questionnaire

For peer review only - http://bmjopen.bmj.com/site/about/guidelines.xhtml

A 9-item bespoke questionnaire to report RN experiences of recruiting to a fractional factorial trial. Demographic and role information will also be collected for the RNs.

Process evaluation

Fidelity of design

Two independent coders will identify and record which BCTs from the BCT Taxonomy v1 are present in each intervention component [48].

Fidelity of training

Each therapist's first ACT session will be assessed for competency using the ACT-FM by a clinically trained member of the research team (CG).

Fidelity of delivery

We will collect SMS receipt data, record the number of information leaflets and website details sent to participants, use independently rated competency scores on the ACT-FM therapist stance subscale, and report scores on the procedural fidelity checklist.

Fidelity of receipt

We will use self-reported data on receipt and reading of the materials within an intervention component, SMS opt outs, website tracking data, therapist-reported attendance and engagement with ACT session, and independently assessed engagement with ACT session.

Participant and therapist interviews

Semi-structured interviews with participants will investigate the acceptability, and fidelity of receipt and enactment of the intervention components. ACT therapists will be interviewed, to gain insight into their experiences of intervention delivery, and the acceptability of the ACT component. Interviews lasting approximately one hour will be conducted via video conferencing software or telephone.

Sample Size

The primary objective is to estimate rates of eligibility, recruitment, retention, and component adherence to inform the future trial. Allowing for 80% retention (the average across 8 studies [20]), 80 participants (n=10 per condition) will be randomised. This is sufficient to inform the sample size of the optimisation trial assuming adherence data are pooled across conditions [96].

For peer review only - http://bmjopen.bmj.com/site/about/guidelines.xhtml

Patient and Public Involvement

A user group of 5 breast cancer survivors have supported the development of the intervention components and trial protocol. Two representatives are members of the trial management group.

Independent Monitoring

The Trial Steering Committee, with an independent Chair, will be responsible for trial oversight. The TSC will be provided with reports prepared by the CTRU based on an agreed TSC Charter and containing the information agreed in the trial monitoring plan. It includes an Independent Chair, at least two other independent members, and two patient representatives. The Chief Investigator and other members of the TMG may attend the TSC meetings and report progress. The TSC will adopt a safety function.

For peer review only

Data Management

Data collection forms transferred to or from the CTRU will be coded with a study number (made up of the recruitment site code and the participant's unique sequential trial number), the participant's initials, and date of birth. Study data will be held securely on paper and electronically at the University of Leeds CTRU, and appropriate processes put in place for the transfer, storage, restricted access, and disposal of personal information. Relevant standard operating procedures, guidelines and work instructions in relation to data management, processing, and analysis of data will be followed.

Analysis

A detailed statistical analysis plan will be written and signed off before analysis is undertaken. The analysis will focus on descriptive statistics and confidence interval estimation rather than formal hypothesis testing. All analyses will be undertaken on the intention-to-treat population, with all participants included in the analysis according to their randomised allocation, and regardless of non-adherence to the intervention components or withdrawal from the trial. Final analysis will be conducted once all available outcome data is received. Outcome measures will be scored according to relevant scoring manuals. Pre-defined progression criteria will be used to judge the feasibility of progressing to the optimisation trial (Table 5). Proof of principle will be explored via investigation of between-group change in outcomes. Point estimates and 95% confidence intervals will be presented for the main effects and interaction effects for adherence and quality of life outcomes. Point estimates and 95% confidence intervals for process variables will be presented for the intervention components in which a change is hypothesised. Analysis will adjust for the stratification factor.

For peer review only - http://bmjopen.bmj.com/site/about/guidelines.xhtml

Table 5 Progression criteria for deciding whether or not to proceed to the optimisation trial

	Green	Amber	Red
Eligible patients consent rate	≥30%	≥10%	<10%
Component adherence			
75% of SMS messages received with no opt out	≥50%	≥20%	<20%
Read 'at least some' of the information leaflet	≥50%	≥20%	<20%
Completed 2/4 ACT modules	≥50%	≥20%	<20%
Registered and logged onto website at least once	≥50%	≥20%	<20%
Availability of adherence measures with ≥75% complete data	≥2	≥1	0

Green (go): optimisation phase is feasible with no changes to design or procedures; Amber (modify): optimisation phase is feasible following minor enhancement of procedures; Red (stop): optimisation phase is not feasible.

A rapid evaluation approach will be used for the qualitative interviews [97]. Rapid Research Evaluation and Appraisal Lab (RREAL) sheets, which are summary tables collected after an interview, will be created for each individual participant, and collated into higher level summary sheets [98]. Regular team meetings will discuss emerging findings, changes to the interview schedule, and data saturation. The interviews will be recorded. The RREAL sheets will be used to guide analysis, and illustrative quotes used to support themes.

Ethics and dissemination

Ethical approval and data

The trial has been approved by Wales Research Authority Research Ethics Committee 3 (21/WA/0322) and is a registered clinical trial (ISRCTN10487576). It is sponsored by the University of Leeds (governance-ethics@leeds.ac.uk). Amendments to the protocol will be submitted to the ethics committee, and if approved, communicated to research sites. Trial findings will be disseminated through peer-reviewed publications. At the end of the trial, all data held by the CTRU and all trial data will then be securely archived at the University of Leeds for a minimum of 5 years. This paper is a summary of protocol version 3.0 (18/08/2022), available upon request from the corresponding author.

Dissemination

For peer review only - http://bmjopen.bmj.com/site/about/guidelines.xhtml

Results will be presented at scientific meetings and published in international peer-reviewed journals. Authorship decisions will be guided by the International Committee of Medical Journal Editors criteria. Summaries will be provided to participants and the trial funder.

Safety

We expect episodes of acute illness, infection, new medical problems and deterioration of existing medical problems will occur and could result in prolonged hospitalisation, hospital re-admission, significant or permanent disability or incapacity, or death. In recognition of this, events fulfilling the definition of a serious adverse event (SAE) will not be reported unless the event resulted from administration of any research procedure, and fulfils the definition of a related and unexpected serious adverse event (RUSAE). Reports of physical self-harm will also be considered Serious Adverse Events and assessed for relatedness and unexpectedness. We might anticipate the following adverse events related to the intervention; low mood (including suicidal thoughts and plans), fatigue, anxiety and/or psychological distress. Adverse events relating to these factors will not be reported as a RUSAE.

Safety will be monitored through observed increases in 'extremely severe' DASS-21 scores for anxiety, stress and depression, a planned RN phone call at 4 months, ad-hoc RN phone calls if a participant wishes to disclose a mental health crisis or referral, disclosures within the semi-structured interviews and ACT sessions. Routinely collected hospital data will be used to record deaths, hospitalisations and mental health crisis referrals.

Access to data

Data will only be shared for participants who have given consent to use of their data for secondary research, and will only be made available in such a way that recipients cannot identify individuals by any reasonable likely means. We will only share data for projects that have ethical approval granted, are clearly in the public interest and compatible with the original purpose of the data processing. Requests to access trial data should be made to CTRU-DataAccess@leeds.ac.uk in the first instance.

Recruitment status

The trial opened for recruitment on the 20th May 2022, and was open at the time of submission.

Author contributions

For peer review only - http://bmjopen.bmj.com/site/about/guidelines.xhtml

Trial concept and trial design: All authors; Acquisition of the data: not applicable; Analysis of the data: not applicable; Interpretation of the data: not applicable; Reviewing of the manuscript drafts and approval of the final draft to be submitted for publication: All authors. SS is the guarantor.

Funding

This trial is funded by the National Institute for Health and Social Care Research (NIHR300588) (PI: Samuel Smith). Smith is also supported by a Yorkshire Cancer Research Fellowship (L417). The funder(s) had no role in the design of the trial, data collection, analysis, interpretation of data, and in the writing of this manuscript. RF is supported by grant funding from the National Institute of Health and Social Care Research and Yorkshire Cancer Research (N/A). DF is supported by the Manchester Biomedical Research Centre (IS-BRC-1215-20007).

Competing interest

The authors declare no competing interests.

Acknowledgements

We would like to thank our patient and public involvement members; Rebecca Whittemore, Michaela Kneafsey and Hazel Abey. We also thank the Trial Steering Committee for their advice in designing and delivering the trial.

For peer review only

References

1 Smittenaar CR, Petersen KA, Stewart K, *et al.* Cancer incidence and mortality projections in the UK until 2035. *Br J Cancer* 2016;**115**:1147–55. doi:10.1038/bjc.2016.304

2 Chirgwin JH, Giobbie-Hurder A, Coates AS, *et al.* Treatment Adherence and Its Impact on Disease-Free Survival in the Breast International Group 1-98 Trial of Tamoxifen and Letrozole, Alone and in Sequence. *J Clin Oncol* 2016;**34**:2452–9. doi:10.1200/JCO.2015.63.8619

3 Dezentjé VO, van Blijderveen NJC, Gelderblom H, *et al.* Effect of concomitant CYP2D6 inhibitor use and tamoxifen adherence on breast cancer recurrence in early-stage breast cancer. *J Clin Oncol* 2010;**28**:2423–9. doi:10.1200/JCO.2009.25.0894

4 Ma AMT, Barone J, Wallis AE, *et al.* Noncompliance with adjuvant radiation, chemotherapy, or hormonal therapy in breast cancer patients. *Am J Surg* 2008;**196**:500–4. doi:10.1016/j.amjsurg.2008.06.027

5 Makubate B, Donnan PT, Dewar JA, *et al.* Cohort study of adherence to adjuvant endocrine therapy, breast cancer recurrence and mortality. *Br J Cancer* 2013;**108**:1515–24. doi:10.1038/bjc.2013.116

6 McCowan C, Shearer J, Donnan PT, *et al.* Cohort study examining tamoxifen adherence and its relationship to mortality in women with breast cancer. *Br J Cancer* 2008;**99**:1763–8. doi:10.1038/sj.bjc.6604758

7 McCowan C, Wang S, Thompson AM, *et al.* The value of high adherence to tamoxifen in women with breast cancer: a community-based cohort study. *Br J Cancer* 2013;**109**:1172–80. doi:10.1038/bjc.2013.464

8 Winn AN, Dusetzina SB. The association between trajectories of endocrine therapy adherence and mortality among women with breast cancer. *Pharmacoepidemiol Drug Saf* 2016;**25**:953–9. doi:10.1002/pds.4012

9 Yood MU, Owusu C, Buist DSM, *et al.* Mortality impact of less-than-standard therapy in older breast cancer patients. *J Am Coll Surg* 2008;**206**:66–75. doi:10.1016/j.jamcollsurg.2007.07.015

10 Murphy CC, Bartholomew LK, Carpentier MY, *et al.* Adherence to adjuvant hormonal therapy among breast cancer survivors in clinical practice: a systematic review. *Breast Cancer Res Treat* 2012;**134**:459–78. doi:10.1007/s10549-012-2114-5

11 Owusu C, Buist DSM, Field TS, *et al.* Predictors of tamoxifen discontinuation among older women with estrogen receptor-positive breast cancer. *J Clin Oncol* 2008;**26**:549–55. doi:10.1200/JCO.2006.10.1022

12 Smith SG, Sestak I, Howell A, *et al.* Participant-reported symptoms and their effect on long-term adherence in the International Breast Cancer Intervention Study I (IBIS I). *J Clin Oncol* 2017; **35**:2666-2673. doi:10.1200/JCO.2016.71.7439

13 Sestak I, Smith SG, Howell A, *et al.* Early participant-reported symptoms as predictors of adherence to anastrozole in the International Breast Cancer Intervention Studies II. *Ann Oncol* 2017. **29**:504-509. doi: 10.1093/annonc/mdx713.

For peer review only - http://bmjopen.bmj.com/site/about/guidelines.xhtml

14 Kimmick G, Edmond SN, Bosworth HB, *et al.* Medication taking behaviors among breast cancer patients on adjuvant endocrine therapy. *The Breast* 2015;**24**:630–6. doi:10.1016/j.breast.2015.06.010

15 Wouters H, Stiggelbout AM, Bouvy ML, *et al.* Endocrine Therapy for Breast Cancer: Assessing an Array of Women's Treatment Experiences and Perceptions, Their Perceived Self-Efficacy and Nonadherence. *Clin Breast Cancer* 2014;**14**:460-467.e2. doi:10.1016/j.clbc.2014.04.005

16 Atkins L, Fallowfield L. Intentional and non-intentional non-adherence to medication amongst breast cancer patients. *Eur J Cancer* 2006;**42**:2271–6. doi:10.1016/j.ejca.2006.03.004

17 Green S, French D, Graham C, *et al.* Supporting adjuvant endocrine therapy adherence in women with breast cancer: the development of a complex behavioural intervention using Intervention Mapping guided by the Multiphase Optimisation Strategy. 2022. *BMC Health Serv Res* 2022;**22:**1081. https://doi.org/10.1186/s12913-022-08243-4

18 Hurtado-de-Mendoza A, Cabling ML, Lobo T, *et al.* Behavioral Interventions to Enhance Adherence to Hormone Therapy in Breast Cancer Survivors: A Systematic Literature Review. *Clin Breast Cancer* 2016;**16**:247-255.e3. doi:10.1016/j.clbc.2016.03.006

19 Heiney SP, Parker PD, Felder TM, *et al.* A systematic review of interventions to improve adherence to endocrine therapy. *Breast Cancer Res Treat* 2019. **173**:499-510 doi:10.1007/s10549-018-5012-7

20 Finitsis DJ, Vose BA, Mahalak JG, *et al.* Interventions to promote adherence to Endocrine Therapy among breast cancer survivors: A meta-analysis. *Psychooncology* 2018. **28**:255-263. doi: 10.1002/pon.4959.

21 Yap AF, Thirumoorthy T, Kwan YH. Systematic review of the barriers affecting medication adherence in older adults. *Geriatr Gerontol Int* 2016;**16**:1093–101. doi:10.1111/ggi.12616

22 Collins LM. *Optimization of Behavioral, Biobehavioral, and Biomedical Interventions: The Multiphase Optimization Strategy (MOST)*. Springer International Publishing 2018. //www.springer.com/gb/book/9783319722054 (accessed 11 Sep 2018).

23 Guastaferro K, Collins LM. Achieving the goals of translational science in public health intervention research: The multiphase optimization strategy (MOST). *Am J Public Health*. 2019;**109**:S128–S129.

24 Collins LM, Strayhorn JC, Vanness DJ. One view of the next decade of research on behavioral and biobehavioral approaches to cancer prevention and control: intervention optimization. *Transl Behav Med* 2021;**11**:1998–2008. doi:10.1093/tbm/ibab087

25 Guastaferro K, Collins LM. Optimization Methods and Implementation Science: An Opportunity for Behavioral and Biobehavioral Interventions. *Implement Res Pract* 2021;**2**:26334895211054364. doi:10.1177/26334895211054363

26 Eldredge LKB, Markham CM, Ruiter RA, *et al. Planning health promotion programs: an intervention mapping approach*. John Wiley & Sons 2016.

27 Moon Z, Moss-Morris R, Hunter MS, *et al.* Understanding tamoxifen adherence in women with breast cancer: A qualitative study. *Br J Health Psychol* 2017;**22**:978–97. doi:10.1111/bjhp.12266

For peer review only - http://bmjopen.bmj.com/site/about/guidelines.xhtml

28 Cahir C, Guinan E, Dombrowski SU, *et al.* Identifying the determinants of adjuvant hormonal therapy medication taking behaviour in women with stages I–III breast cancer: A systematic review and meta-analysis. *Patient Educ Couns* 2015;**98**:1524–39. doi:10.1016/j.pec.2015.05.013

29 Brett J, Fenlon D, Boulton M, *et al.* Factors associated with intentional and unintentional non-adherence to adjuvant endocrine therapy following breast cancer. *Eur J Cancer Care* 2018;**27**. doi:10.1111/ecc.12601

30 Toivonen KI, Williamson TM, Carlson LE, *et al.* Potentially Modifiable Factors Associated with Adherence to Adjuvant Endocrine Therapy among Breast Cancer Survivors: A Systematic Review. *Cancers* 2020;**13**:107. doi:10.3390/cancers13010107

31 Green SMC, Hall LH, Rousseau N, *et al.* Acceptability, fidelity and trial experience of four intervention components to support medication adherence in women with breast cancer: A process evaluation protocol for a pilot fractional factorial trial. Submitted.

32 Chan A-W, Tetzlaff JM, Altman DG, *et al.* SPIRIT 2013 statement: defining standard protocol items for clinical trials. *Ann Intern Med* 2013;**158**:200–7. doi:10.7326/0003-4819-158-3-201302050-00583

33 Eldridge SM, Chan CL, Campbell MJ, *et al.* CONSORT 2010 statement: extension to randomised pilot and feasibility trials. *BMJ* 2016;**355**:i5239. doi:10.1136/bmj.i5239

34 Hoffmann TC, Glasziou PP, Boutron I, *et al.* Better reporting of interventions: template for intervention description and replication (TIDieR) checklist and guide. *BMJ* 2014;**348**:g1687. doi:10.1136/bmj.g1687

35 Pullens MJJ, De Vries J, Roukema JA. Subjective cognitive dysfunction in breast cancer patients: a systematic review. *Psychooncology* 2010;**19**:1127–38. doi:10.1002/pon.1673

36 Underwood EA, Rochon PA, Moineddin R, *et al.* Cognitive sequelae of endocrine therapy in women treated for breast cancer: a meta-analysis. *Breast Cancer Res Treat* 2018;**168**:299–310. doi:10.1007/s10549-017-4627-4

37 Kirk MC, Hudis CA. Insight into barriers against optimal adherence to oral hormonal therapy in women with breast cancer. *Clin Breast Cancer* 2008;**8**:155–61. doi:10.3816/CBC.2008.n.016

38 Stilley CS, Bender CM, Dunbar-Jacob J, *et al.* The impact of cognitive function on medication management: Three studies. *Health Psychol* 2010;**29**:50–5. doi:10.1037/a0016940

39 Danner UN, Aarts H, de Vries NK. Habit formation and multiple means to goal attainment: repeated retrieval of target means causes inhibited access to competitors. *Pers Soc Psychol Bull* 2007;**33**:1367–79. doi:10.1177/0146167207303948

40 Gardner B, Lally P. Modelling Habit Formation and Its Determinants. In: Verplanken B, ed. *The Psychology of Habit: Theory, Mechanisms, Change, and Contexts*. Cham: Springer International Publishing 2018. 207–29. doi:10.1007/978-3-319-97529-0_12

41 Gardner B, Lally P, Wardle J. Making health habitual: the psychology of 'habit-formation' and general practice. *Br J Gen Pract* 2012;**62**:664–6. doi:10.3399/bjgp12X659466

42 Lally P, Gardner B. Promoting habit formation. *Health Psychol Rev* 2013;**7**:S137–58. doi:10.1080/17437199.2011.603640

For peer review only - http://bmjopen.bmj.com/site/about/guidelines.xhtml

43 Thakkar J, Kurup R, Laba T-L, *et al.* Mobile Telephone Text Messaging for Medication Adherence in Chronic Disease: A Meta-analysis. *JAMA Intern Med* 2016;**176**:340–9. doi:10.1001/jamainternmed.2015.7667

44 Finitsis DJ, Pellowski JA, Johnson BT. Text Message Intervention Designs to Promote Adherence to Antiretroviral Therapy (ART): A Meta-Analysis of Randomized Controlled Trials. *PLOS ONE* 2014;**9**:e88166. doi:10.1371/journal.pone.0088166

45 Free C, Phillips G, Galli L, *et al.* The effectiveness of mobile-health technology-based health behaviour change or disease management interventions for health care consumers: a systematic review. *PLoS Med* 2013;**10**:e1001362. doi:10.1371/journal.pmed.1001362

46 Ross XS, Gunn KM, Patterson P, *et al.* Mobile-Based Oral Chemotherapy Adherence–Enhancing Interventions: Scoping Review. *JMIR MHealth UHealth* 2018;**6**:e11724. doi:10.2196/11724

47 Green S, French D, Hall L, *et al. Co-development of a text messaging intervention to support adherence to adjuvant endocrine therapy in women with breast cancer: A mixed-methods approach (Preprint)*. 2022. doi:10.2196/preprints.38073

48 Michie S, Richardson M, Johnston M, *et al.* The behavior change technique taxonomy (v1) of 93 hierarchically clustered techniques: building an international consensus for the reporting of behavior change interventions. *Ann Behav Med* 2013;**46**:81–95.

49 Moon Z, Moss-Morris R, Hunter MS, *et al.* Barriers and facilitators of adjuvant hormone therapy adherence and persistence in women with breast cancer: a systematic review. *Patient Prefer Adherence* 2017;**11**:305–22. doi:10.2147/PPA.S126651

50 Pan Y, Heisig SR, von Blanckenburg P, *et al.* Facilitating adherence to endocrine therapy in breast cancer: stability and predictive power of treatment expectations in a 2-year prospective study. *Breast Cancer Res Treat* 2018;**168**:667–77. doi:10.1007/s10549-017-4637-2

51 Nestoriuc Y, von Blanckenburg P, Schuricht F, *et al.* Is it best to expect the worst? Influence of patients' side-effect expectations on endocrine treatment outcome in a 2-year prospective clinical cohort study. *Ann Oncol* 2016;**27**:1909–15. doi:10.1093/annonc/mdw266

52 Horne R, Chapman S, Parham R, *et al.* Understanding patients' adherence-related beliefs about medicines prescribed for long-term conditions: a meta-analytic review of the Necessity-Concerns Framework. *PLoS ONE* 2013;**8**:*e80633. doi: 10.1371/journal.pone.0080633* .

53 Leventhal H, Diefenbach M, Leventhal EA. Illness cognition: Using common sense to understand treatment adherence and affect cognition interactions. *Cogn Ther Res* 1992;**16**:143–63. doi:10.1007/BF01173486

54 Gold M, Dunn LB, Phoenix B, *et al.* Co-occurrence of anxiety and depressive symptoms following breast cancer surgery and its impact on quality of life. *Eur J Oncol Nurs* 2016;**20**:97–105. doi:10.1016/j.ejon.2015.06.003

55 Martino G, Catalano A, Agostino RM, *et al.* Quality of life and psychological functioning in postmenopausal women undergoing aromatase inhibitor treatment for early breast cancer. *PloS One* 2020;**15**:e0230681. doi:10.1371/journal.pone.0230681

For peer review only

56 Jacobs JM, Walsh EA, Park ER, *et al.* The Patient's Voice: Adherence, Symptoms, and Distress Related to Adjuvant Endocrine Therapy After Breast Cancer. *Int J Behav Med* 2020;**27**:687–97. doi:10.1007/s12529-020-09908-2

57 Peddie N, Agnew S, Crawford M, *et al.* The impact of medication side effects on adherence and persistence to hormone therapy in breast cancer survivors: A qualitative systematic review and thematic synthesis. *Breast* 2021;**58**:147–59. doi:10.1016/j.breast.2021.05.005

58 Clancy C, Lynch J, OConnor P, *et al.* Breast cancer patients' experiences of adherence and persistence to oral endocrine therapy: A qualitative evidence synthesis. *Eur J Oncol Nurs* 2020;**44**:101706. doi:10.1016/j.ejon.2019.101706

59 Graham CD, McCracken LM, Harrison A, *et al.* Outlining an Acceptance and Commitment Therapy approach to treatment non-adherence. *Br J Health Psychol* 2021;:1–12.

60 Graham CD, Gouick J, Krahé C, *et al.* A systematic review of the use of Acceptance and Commitment Therapy (ACT) in chronic disease and long-term conditions. *Clin Psychol Rev* 2016;**46**:46–58. doi:10.1016/j.cpr.2016.04.009

61 Rose M, Graham CD, O'Connell N, *et al.* A randomised controlled trial of acceptance and commitment therapy for improving quality of life in people with muscle diseases. *Psychol Med* 2022;:1–14. doi:10.1017/S0033291722000083

62 Cella D, Fallowfield LJ. Recognition and management of treatment-related side effects for breast cancer patients receiving adjuvant endocrine therapy. *Breast Cancer Res Treat* 2008;**107**:167–80. doi:10.1007/s10549-007-9548-1

63 Coombes RC, Hall E, Gibson LJ, *et al.* A Randomized Trial of Exemestane after Two to Three Years of Tamoxifen Therapy in Postmenopausal Women with Primary Breast Cancer. *N Engl J Med* 2004;**350**:1081–92. doi:10.1056/NEJMoa040331

64 Goss PE, Ingle JN, Martino S, *et al.* A Randomized Trial of Letrozole in Postmenopausal Women after Five Years of Tamoxifen Therapy for Early-Stage Breast Cancer. *N Engl J Med* 2003;**349**:1793–802. doi:10.1056/NEJMoa032312

65 Howell A, Cuzick J, Baum M, *et al.* Results of the ATAC (Arimidex, Tamoxifen, Alone or in Combination) trial after completion of 5 years' adjuvant treatment for breast cancer. *Lancet* 2005;**365**:60–2. doi:10.1016/S0140-6736(04)17666-6

66 Jakesz R, Jonat W, Gnant M, *et al.* Switching of postmenopausal women with endocrine-responsive early breast cancer to anastrozole after 2 years' adjuvant tamoxifen: combined results of ABCSG trial 8 and ARNO 95 trial. *Lancet* 2005;**366**:455–62. doi:10.1016/S0140-6736(05)67059-6

67 Breast International Group (BIG) 1-98 Collaborative Group, Thürlimann B, Keshaviah A, *et al.* A comparison of letrozole and tamoxifen in postmenopausal women with early breast cancer. *N Engl J Med* 2005;**353**:2747–57. doi:10.1056/NEJMoa052258

68 Chlebowski RT, Kim J, Haque R. Adherence to endocrine therapy in breast cancer adjuvant and prevention settings. *Cancer Prev Res Phila Pa* 2014;**7**:378–87. doi:10.1158/1940-6207.CAPR-13-0389

For peer review only - http://bmjopen.bmj.com/site/about/guidelines.xhtml

69 Cella D, Fallowfield L, Barker P, *et al.* Quality of Life of Postmenopausal Women in the ATAC ("Arimidex", Tamoxifen, Alone or in Combination) Trial after Completion of 5 years' Adjuvant Treatment for Early Breast Cancer. *Breast Cancer Res Treat* 2006;**100**:273–84. doi:10.1007/s10549-006-9260-6

70 Fallowfield L, Cella D, Cuzick J, *et al.* Quality of Life of Postmenopausal Women in the Arimidex, Tamoxifen, Alone or in Combination (ATAC) Adjuvant Breast Cancer Trial. *J Clin Oncol* 2004;**22**:4261–71. doi:10.1200/JCO.2004.08.029

71 Ganz PA, Petersen L, Bower JE, *et al.* Impact of Adjuvant Endocrine Therapy on Quality of Life and Symptoms: Observational Data Over 12 Months From the Mind-Body Study. *J Clin Oncol* 2016;**34**:816–24. doi:10.1200/JCO.2015.64.3866

72 Schover LR, Baum GP, Fuson LA, *et al.* Sexual problems during the first 2 years of adjuvant treatment with aromatase inhibitors. *J Sex Med* 2014;**11**:3102–11. doi:10.1111/jsm.12684

73 Demissie S, Silliman RA, Lash TL. Adjuvant tamoxifen: predictors of use, side effects, and discontinuation in older women. *J Clin Oncol* 2001;**19**:322–8. doi:10.1200/JCO.2001.19.2.322

74 Kahn KL, Schneider EC, Malin JL, *et al.* Patient centered experiences in breast cancer: predicting long-term adherence to tamoxifen use. *Med Care* 2007;**45**:431–9. doi:10.1097/01.mlr.0000257193.10760.7f

75 Meggetto O, Maunsell E, Chlebowski R, *et al.* Factors Associated With Early Discontinuation of Study Treatment in the Mammary Prevention.3 Breast Cancer Chemoprevention Trial. *J Clin Oncol* 2017;**35**:629–35. doi:10.1200/JCO.2016.68.8895

76 Fleming L, Agnew S, Peddie N, *et al.* The impact of medication side effects on adherence and persistence to hormone therapy in breast cancer survivors: A quantitative systematic review. *Breast Edinb Scotl* 2022;**64**:63–84. doi:10.1016/j.breast.2022.04.010

77 Wheeler SB, Roberts MC, Bloom D, *et al.* Oncology providers' perspectives on endocrine therapy prescribing and management. *Patient Prefer Adherence* 2016;**10**:2007–19. doi:10.2147/PPA.S95594

78 Brett J, Boulton M, Fenlon D, *et al.* Adjuvant endocrine therapy after breast cancer: a qualitative study of factors associated with adherence. *Patient Prefer Adherence* 2018;**12**:291–300. doi:10.2147/PPA.S145784

79 Peate M, Saunders C, Cohen P, *et al.* Who is managing menopausal symptoms, sexual problems, mood and sleep disturbance after breast cancer and is it working? Findings from a large community-based survey of breast cancer survivors. *Breast Cancer Res Treat* 2021;**187**:427-435:doi:10.1007/s10549-021-06117-7

80 Hall LH, King NV, Graham CD, *et al.* Strategies to self-manage side-effects of adjuvant endocrine therapy among breast cancer survivors: an umbrella review of empirical evidence and clinical guidelines. *J Cancer Surviv Res Pract* 2021;**16**:1296-1338. doi:10.1007/s11764-021-01114-7

81 Hall LH, Clark J, Smith S, *et al.* Patient and healthcare professional co-development of an Acceptance and Commitment Therapy intervention to support Hormone Therapy decision-making and quality of life in women with breast cancer. J Psy Soc Oncol 2022;**40**; 407-424. https://doi.org/10.1080/07347332.2021.1955318

For peer review only - http://bmjopen.bmj.com/site/about/guidelines.xhtml

82 Morisky DE, Green LW, Levine DM. Concurrent and predictive validity of a self-reported measure of medication adherence. *Med Care* 1986;**24**:67–74.

83 Voils CI, King HA, Thorpe CT, *et al.* Content Validity and Reliability of a Self-Report Measure of Medication Nonadherence in Hepatitis C Treatment. *Dig Dis Sci* 2019;**64**:2784–97. doi:10.1007/s10620-019-05621-7

84 Aaronson NK, Ahmedzai S, Bergman B, *et al.* The European Organization for Research and Treatment of Cancer QLQ-C30: a quality-of-life instrument for use in international clinical trials in oncology. *J Natl Cancer Inst* 1993;**85**:365–76. doi:10.1093/jnci/85.5.365

85 Bjelic-Radisic V, Cardoso F, Cameron D, *et al.* An international update of the EORTC questionnaire for assessing quality of life in breast cancer patients: EORTC QLQ-BR45. *Ann Oncol* 2020;**31**:283–8. doi:10.1016/j.annonc.2019.10.027

86 Rabin R, de Charro F. EQ-5D: a measure of health status from the EuroQol Group. *Ann Med* 2001;**33**:337–43.

87 Rolffs JL, Rogge RD, Wilson KG. Disentangling Components of Flexibility via the Hexaflex Model: Development and Validation of the Multidimensional Psychological Flexibility Inventory (MPFI). *Assessment* 2018;**25**:458–82. doi:10.1177/1073191116645905

88 Brett J, Hulbert-Williams NJ, Fenlon D, *et al.* Psychometric properties of the Beliefs about Medicine Questionnaire–adjuvant endocrine therapy (BMQ-AET) for women taking AETs following early-stage breast cancer. *Health Psychol Open* 2017;**4**:2055102917740469. doi:10.1177/2055102917740469

89 Gardner B, Abraham C, Lally P, *et al.* Towards parsimony in habit measurement: Testing the convergent and predictive validity of an automaticity subscale of the Self-Report Habit Index. *Int J Behav Nutr Phys Act* 2012;**9**:102. doi:10.1186/1479-5868-9-102

90 Antony MM, Bieling PJ, Cox BJ, *et al.* Psychometric properties of the 42-item and 21-item versions of the Depression Anxiety Stress Scales in clinical groups and a community sample. *Psychol Assess* 1998;**10**:176–81. doi:10.1037/1040-3590.10.2.176

91 Cohen SR, Sawatzky R, Russell LB, *et al.* Measuring the quality of life of people at the end of life: The McGill Quality of Life Questionnaire-Revised. *Palliat Med* 2017;**31**:120–9. doi:10.1177/0269216316659603

92 UK Cancer Costs Questionnaire. UK Cancer Costs Questionnaire (accessed 12 Mar 2021: https://blogs.ed.ac.uk/ukcc/).

93 Sekhon M, Cartwright M, Francis JJ. Development of a theory-informed questionnaire to assess the acceptability of healthcare interventions. *BMC Health Serv Res* 2022;**22**:279. doi:10.1186/s12913-022-07577-3

94 Greene A, Elmer M, Ludlam S, *et al.* Evaluation of the Content Validity and Cross-Cultural Validity of the Study Participant Feedback Questionnaire (SPFQ). *Ther Innov Regul Sci* 2020;**54**:1522–33. doi:10.1007/s43441-020-00179-3

95 O'Neill L, Latchford G, McCracken L, *et al.* The Development of the Acceptance and Commitment Therapy Fidelity Measure (ACT-FM): A Delphi Study and Field Test. *J Context Behav Sci* 2019:111-118. doi:10.1016/j.jcbs.2019.08.008

For peer review only

For peer review only - http://bmjopen.bmj.com/site/about/guidelines.xhtml

96 Teare MD, Dimairo M, Shephard N, *et al.* Sample size requirements to estimate key design parameters from external pilot randomised controlled trials: a simulation study. *Trials* 2014;**15**:264. doi:10.1186/1745-6215-15-264

97 Vindrola-Padros C, Johnson GA. Rapid techniques in qualitative research: A critical review of the literature. *Qual Health Res* 2020;**30**:1596–1604.

98 Vindrola-Padros C, Chisnall G, Polanco N, *et al.* Iterative cycles in qualitative research: Introducing the RREAL Sheet as an innovative process. 2022: doi:https://doi.org/10.31219/osf.io/9dp2w

For peer review only

For peer review only - http://bmjopen.bmj.com/site/about/guidelines.xhtml

For peer review only

For peer review only - http://bmjopen.bmj.com/site/about/guidelines.xhtml

For peer review only

For peer review only - http://bmjopen.bmj.com/site/about/guidelines.xhtml

For peer review only

For peer review only - http://bmjopen.bmj.com/site/about/guidelines.xhtml

CONSORT 2010 checklist of information to include when reporting a pilot or feasibility trial*

Section/Topic	Item No	Checklist item	Reported on page No
**Title and abstract**			
	1a	Identification as a pilot or feasibility randomised trial in the title	1
	1b	Structured summary of pilot trial design, methods, results, and conclusions (for specific guidance see CONSORT abstract extension for pilot trials)	2
**Introduction**			
Background and objectives	2a	Scientific background and explanation of rationale for future definitive trial, and reasons for randomised pilot trial	4-5
	2b	Specific objectives or research questions for pilot trial	5
**Methods**			
Trial design	3a	Description of pilot trial design (such as parallel, factorial) including allocation ratio	5
	3b	Important changes to methods after pilot trial commencement (such as eligibility criteria), with reasons	N/A
Participants	4a	Eligibility criteria for participants	6 (Table 2)
	4b	Settings and locations where the data were collected	6
	4c	How participants were identified and consented	6-7
Interventions	5	The interventions for each group with sufficient details to allow replication, including how and when they were actually administered	7-9
Outcomes	6a	Completely defined prespecified assessments or measurements to address each pilot trial objective specified in 2b, including how and when they were assessed	9-13
	6b	Any changes to pilot trial assessments or measurements after the pilot trial commenced, with reasons	N/A
	6c	If applicable, prespecified criteria used to judge whether, or how, to proceed with future definitive trial	Table 5
Sample size	7a	Rationale for numbers in the pilot trial	13
	7b	When applicable, explanation of any interim analyses and stopping guidelines	N/A
Randomisation:			
Sequence generation	8a	Method used to generate the random allocation sequence	7
	8b	Type of randomisation(s); details of any restriction (such as blocking and block size)	7
Allocation concealment mechanism	9	Mechanism used to implement the random allocation sequence (such as sequentially numbered containers), describing any steps taken to conceal the sequence until interventions were assigned	7

For peer review only

Implementation	10	Who generated the random allocation sequence, who enrolled participants, and who assigned participants to interventions	6-7
Blinding	11a	If done, who was blinded after assignment to interventions (for example, participants, care providers, those assessing outcomes) and how	7
	11b	If relevant, description of the similarity of interventions	7-9
Statistical methods	12	Methods used to address each pilot trial objective whether qualitative or quantitative	
**Results**			
Participant flow (a diagram is strongly recommended)	13a	For each group, the numbers of participants who were approached and/or assessed for eligibility, randomly assigned, received intended treatment, and were assessed for each objective	N/A
	13b	For each group, losses and exclusions after randomisation, together with reasons	N/A
Recruitment	14a	Dates defining the periods of recruitment and follow-up	N/A
	14b	Why the pilot trial ended or was stopped	N/A
Baseline data	15	A table showing baseline demographic and clinical characteristics for each group	N/A
Numbers analysed	16	For each objective, number of participants (denominator) included in each analysis. If relevant, these numbers should be by randomised group	N/A
Outcomes and estimation	17	For each objective, results including expressions of uncertainty (such as 95% confidence interval) for any estimates. If relevant, these results should be by randomised group	N/A
Ancillary analyses	18	Results of any other analyses performed that could be used to inform the future definitive trial	N/A
Harms	19	All important harms or unintended effects in each group (for specific guidance see CONSORT for harms)	N/A
	19a	If relevant, other important unintended consequences	N/A
**Discussion**			
Limitations	20	Pilot trial limitations, addressing sources of potential bias and remaining uncertainty about feasibility	N/A
Generalisability	21	Generalisability (applicability) of pilot trial methods and findings to future definitive trial and other studies	N/A
Interpretation	22	Interpretation consistent with pilot trial objectives and findings, balancing potential benefits and harms, and considering other relevant evidence	N/A
	22a	Implications for progression from pilot to future definitive trial, including any proposed amendments	
**Other information**			
Registration	23	Registration number for pilot trial and name of trial registry	1, 6
Protocol	24	Where the pilot trial protocol can be accessed, if available	15
Funding	25	Sources of funding and other support (such as supply of drugs), role of funders	16
	26	Ethical approval or approval by research review committee, confirmed with reference number	15

For peer review only

Citation: Eldridge SM, Chan CL, Campbell MJ, Bond CM, Hopewell S, Thabane L, et al. CONSORT 2010 statement: extension to randomised pilot and feasibility trials. BMJ. 2016;355.

*We strongly recommend reading this statement in conjunction with the CONSORT 2010, extension to randomised pilot and feasibility trials, Explanation and Elaboration for important clarifications on all the items. If relevant, we also recommend reading CONSORT extensions for cluster randomised trials, non-inferiority and equivalence trials, non-pharmacological treatments, herbal interventions, and pragmatic trials. Additional extensions are forthcoming: for those and for up to date references relevant to this checklist, see www.consort-statement.org.

For peer review only

The TIDieR (Template for Intervention Description and Replication) Checklist*:

Information to include when describing an intervention and the location of the information

Nº	What	Details
**1**	**Name**	Refining and Optimising a behavioural intervention to support endocrine therapy adherence: The ROSETA Pilot Trial.
**2**	**Why: Rationale, theory, goal**	Adjuvant hormone therapies are prescribed at the end of hospital-based breast cancer treatment in order to prevent recurrences and all-cause mortality. However adherence to these medications is often poor, due to multiple factors, including forgetting, beliefs about medications, intolerable side effects and psychological distress. This time during the cancer journey is also particularly challenging, as women are transitioning from 'patient' to 'survivor'. They also report a lack of support during this time, post hospital discharge.<br><br>Previous adherence interventions have tended to consist of solely educational based interventions, that are not grounded in theory, and did not target the factors commonly associated with medication adherence. Given the wide range of barriers to adherence in this population, it is perhaps unsurprising that previous interventions have shown limited effectiveness. An alternative strategy is to design a complex intervention, with multiple components that can target a range of factors that have been highlighted as barriers to adherence.<br><br>*Memory and forgetting*<br><br>Mobile phone-based interventions are well suited to tackle forgetfulness as a barrier to adherence, through reminders and promotion of habit formation. SMS messages have been shown to be effective in improving medication adherence in other chronic illnesses but have not been widely tested in cancer patients.<br><br>*Medication schemas*<br><br>Accurate information about the necessity and risks of AET has the potential to increase women's perceptions of their need for AET, and to reduce unfounded concerns about the medication. In addition, women with breast cancer have stated that they would

For peer review only

		like more accurate information about AET to overcome unfounded concerns.<br><br>*Psychological Flexibility*<br><br>Acceptance and Commitment Therapy (ACT) has been shown to improve outcomes in those living with chronic illness, chronic pain, and cancer. ACT aims to increase a participant's awareness of their personal values, and to undertake more of the behaviours that support these values – a process that often involves developing a willingness to have painful thoughts and feelings (such as medication side-effects). ACT targets psychological flexibility, which can improve functioning during objectively difficult circumstances, and can often reduce psychological distress as a by-product.<br><br>*Living with Side effects*<br><br>One of the most commonly cited barriers to AET adherence is the impact of side effects, and the lack of support for management of these is commonly cited. There are a number of strategies for these side effects that have the potential to be effective in alleviating symptoms. However, these are typically not presented in a patient-friendly manner.<br><br>Given the above, we have co-designed four intervention components for women with breast cancer who have been prescribed adjuvant endocrine therapies; SMS reminder messages to target forgetfulness, an information leaflet to promote formation of accurate beliefs, ACT therapy sessions to increase psychological flexibility, and a side-effect management website to support living with side effects. The aim of the intervention components are to support medication adherence to hormone therapy. Participants will be randomised to receive none, or a combination of one or more of these four components.
**3**	**What Materials**	*Participants randomised to receive SMS component*<br><br>Participants received 43 SMS messages over four months. This included three opening messages, one closing message, 36 messages related to behaviour change techniques aiming to

For peer review only

		promote habit formation, and 3 messages (sent after 1, 2 and 3 months) as a reminder that participants can stop any further SMS messages being sent by emailing the ROSETA team. The content of the SMS messages was co-developed with experts in behaviour change and/or medication adherence, and women who have experienced breast cancer.<br><br>*Participants randomised to receive information leaflet*<br><br>Participants received an information leaflet containing detailed information about AET. This included information about how the medication works (with diagrams to supplement), information about the benefits and side effects of AET, answers to common concerns that women have, and quotes from women with experience of taking AET.<br><br>*ACT*<br><br>*Participants randomised to ACT sessions*<br><br>Participants were emailed a participant manual consisting of information about the ACT skill and home practice tasks, in addition to corresponding audio files to assist with the home practice tasks. Each of the four modules focused on a different ACT-based skill:<br>• Module 1: Mindfulness and unhooking<br>• Module 2: Following your values<br>• Module 3: Taking an observer perspective<br>• Module 4: Recap, reflection, and staying committed<br><br>*Therapists delivering ACT sessions*<br><br>Therapists delivering the intervention received two half days of bespoke training delivered by clinical psychologists with ACT experience. Alongside this, they received a training manual, with information about ACT generally, and specific session plans for the intervention sessions.<br><br>*Participants randomised to receive access to side-effect website*

For peer review only

		Participants allocated to receive the website will receive access to a bespoke website containing information and strategies for self-management of side effects, and signposting to further sources of support.
**4**	**What Procedures**	***Intervention Delivery***<br><br>*Participants randomised to receive SMS component*<br><br>Participants received 43 SMS messages over four months. This included three opening messages, one closing message, 36 messages related to behaviour change techniques aiming to promote habit formation, and 3 messages (sent after 1, 2 and 3 months) as a reminder that participants can stop any further SMS messages being sent by emailing the ROSETA team. The content of the SMS messages was co-developed with experts in behaviour change and/or medication adherence, and women who have experienced breast cancer.<br><br>The 36 messages relating to behaviour change techniques were sent on the following schedule:<br>– Daily messages for 2 weeks<br>– Two messages per week for 8 weeks<br>– Weekly messages for 6 weeks<br><br>*Participants randomised to receive information leaflet*<br><br>Participants were sent the information leaflet by email immediately after randomisation.<br><br>*Participants randomised to receive ACT*<br><br>4x guided self-help modules consisting of information about ACT, home practice exercises and corresponding audio files<br><br>1x 15-minute individual introductory session with a psychologist<br><br>3 x 25 minute individual support sessions with a psychologist to discuss the module completed over the past week, their experiences of the home practice exercises, and to allow discussion and problem solving of any difficulties that arose.<br><br>1x 15-minute closing session with a psychologist

For peer review only

		Participants randomised to receive access to side-effect website<br><br>Participants were given login details of the website immediately after randomisation.<br><br>*Clinician Training*<br><br>(See section 5, below, for information on this)<br><br>*Evaluation of the Clinician Training*<br><br>Recordings of each therapists first session were reviewed by Dr Graham, using the ACT-FM therapist stance subscale.<br><br>Clinician fidelity was evaluated using the ACT-FM therapist stance subscale completed by an external rater. 10% of recorded sessions were evaluated.<br><br>Clinician fidelity to intervention procedures involved clinician self-rating using a procedural fidelity checklist.<br><br>*Evaluation of the Intervention Components*<br><br>Adherence, quality of life/ symptom burden, psychological distress, psychological flexibility, habitual behaviour of medication taking and medication beliefs were all measured pre and post intervention. Acceptability of each intervention component was assessed. Self reported engagement of intervention components was monitored. SMS delivery, and website use was tracked.<br><br>*Support activities*<br><br>Recruitment and consent of participants
**5**	**Who provided**	*SMS messages*<br><br>The CTRU sent all SMS messages to participants.<br><br>*Information Leaflet*<br><br>The information leaflet was sent to participants by the site.

For peer review only

		ACT<br><br>The therapists who delivered the intervention underwent training in delivering Acceptance and Commitment Therapy. The training was delivered by Dr Chris Graham (CG), who has expertise in ACT applied to chronic disease. Training included teaching about ACT and practice of intervention-specific therapy methods. This course consisted of two half days of training.<br><br>Each site's therapists had a varied background that may or may not have included previous ACT training prior to our delivered training programme. However, all session leads were Health and Care Professional Council (HCPC) or UKCP registered practitioner psychologists or Psychotherapists (Clinical, Health or Counselling Psychologist or Psychotherapists) who worked with breast cancer patients in a hospital setting.<br><br>*Website*<br><br>Access to the bespoke website was provided by the site.
**6**	**How: mechanisms of delivery**	*Participants randomised to receive SMS component*<br><br>SMS messages were sent in an automated fashion by the CTRU to the participants mobile phone based on the following schedule:<br>– Daily messages for 2 weeks<br>– Two messages per week for 8 weeks<br>– Weekly messages for 6 weeks<br><br>In addition one message was sent after months 1, 2 and 3 as a reminder that participants can stop any further SMS messages being sent by emailing the ROSETA team.<br><br>*Participants randomised to receive information leaflet*<br><br>Participants were sent the information leaflet electronically immediately after randomisation and were able to read this as they wished.

For peer review only

		Participants randomised to receive ACT<br>The individual sessions (5 in total) were delivered via phone or video conferencing.<br>The participant manual containing information about each module, home practice tasks, and audio files were emailed to each participant by the therapist following each session.<br>*Participants randomised to receive access to side-effect website*<br>Participants were given a login to the website immediately after randomisation and were able to use this as they wished.
**7**	**Where: location of delivery**	*SMS Messages*<br>Not applicable.<br>*Information Leaflet*<br>Not applicable.<br>*ACT*<br>All sessions were delivered remotely via phone or videoconferencing.<br>*Website*<br>Not applicable
**8**	**When and how much**	*Participants randomised to receive SMS component*<br>SMS messages were sent by the CTRU based on the following schedule:<br>– Daily messages for 2 weeks<br>– Two messages per week for 8 weeks<br>– Weekly messages for 6 weeks<br>In addition three opening messages, one closing message and, one message after months 1,2 and 3 were sent reminding participants

For peer review only

		that they could stop any further SMS messages being sent via emailing the ROSETA team.<br><br>*Participants randomised to receive information leaflet*<br><br>Participants were sent the information leaflet electronically immediately after randomisation and were able to read this as they wished.<br><br>*Participants randomised to receive ACT*<br><br>The introductory session lasted 15 minutes, three subsequent sessions lasted 25 minutes, and the final closing session lasted 15 minutes. Participants had their first session within four weeks of randomisation. The therapy sessions were held weekly.<br><br>*Participants randomised to receive access to side-effect website*<br><br>Participants were given a login to the website immediately after randomisation and were able to use this as they wished.
**9**	**Tailoring**	*SMS*<br><br>The same SMS messages were sent in the same order to each participant.<br><br>*Information Leaflet*<br><br>The same information leaflet was sent to each participant.<br><br>*ACT*<br><br>Although there is a set session plan to follow, detailing specific exercises and tasks for each session, the therapy itself is quite flexible. As such, the deliverer may adapt the content to ensure it's relevant to each participant (e.g. through discussing specific individuals' values, goals, and behaviours).

For peer review only

		Website<br><br>The website was the same for each participant.
**10***	**Modifications**	*<To be completed post study completion>*
**11**	**How well (planned)**	*SMS*<br><br>Successful delivery and receipt of the SMS messages will be recorded by the CTRU, alongside the number of SMS messages that were unable to be delivered. Participants will answer a single item asking whether they received the SMS messages, and another item asking how many of the SMS messages they read. Semi-structured interviews were conducted to understand fidelity of receipt and enactment of the messages.<br><br>*Information Leaflet*<br><br>The number of information leaflets sent out to participants was recorded. This will be recorded by the site when each information leaflet is sent out.<br><br>Participants were asked to self-report whether they received the information leaflet, and how much of the information leaflet they read. Semi-structured interviews were conducted with participants to understand the fidelity of receipt and enactment of the information leaflet.<br><br>*ACT*<br><br>Clinician fidelity to competently deliver the intervention in line with ACT was assessed by an external rater with a background in ACT. They completed the ACT-FM therapist stance subscale checklist whilst listening to the audio recording of 10% of sessions. A score of >4 on ACT consistent behaviours and <5 on ACT inconsistent behaviours is considered competent.<br><br>Additionally, an intervention specific metric of "Procedural Fidelity" was included, which measures other aspects of the intervention that are important for treatment fidelity but are not ACT-specific (e.g. reflecting on home practice tasks, sending module content etc). Therapists will complete the procedural fidelity checklist following each session. A percentage score is created for each session by dividing the score achieved by the

For peer review only

		maximum possible score achievable within that session and multiplying by 100.<br><br>Fidelity of ACT training was monitored through Dr Graham assessing the recording of each therapists first ACT session, and rating competency based on the ACT-FM therapist stance subscale. A score of >4 on ACT consistent behaviours and <5 on ACT inconsistent behaviours is considered competent. Semi-structured interviews with psychologists assessed the fidelity of training and delivery of the ACT component.<br><br>Participant fidelity to the ACT component was monitored by recording the number of sessions attended, missed and cancelled. The therapist additionally reported how much of the module materials the participant had read and engaged with (participant manual, audio files and home practice tasks). Participants self-reported receipt of the module content, self-reported engagement with the participant manual, audio files and home practice tasks. Semi-structured interviews additionally assessed fidelity of receipt and enactment.<br><br>Additionally 10% of recorded sessions were evaluated by an independent reviewer in order to review and assess the recording of a participants engagement in home work tasks by the therapist.<br><br>*Website*<br><br>Website data was tracked for each participant, including number of logins, time spent on pages, videos watched and clicked links. Participants were asked a single item about their engagement with the website. Fidelity of receipt and enactment were additionally assessed through semi-structured interviews.
**12***	**How well (actual)**	*<To be completed post study>*

For peer review only

BMJ Open

Refining and Optimising a behavioural intervention to Support Endocrine Therapy Adherence (ROSETA) in UK women with breast cancer: protocol for a pilot fractional factorial trial

Journal:	*BMJ Open*
Manuscript ID	bmjopen-2022-069971.R1
Article Type:	Protocol
Date Submitted by the Author:	13-Jan-2023
Complete List of Authors:	Smith, Samuel; University of Leeds Leeds Institute of Health Sciences<br>Green, Sophie M. C.; Leeds Institute of Health Science University of Leeds<br>Ellison, Rachel; University of Leeds, Clinical Trials Research Unit<br>Foy, Robbie; University of Leeds, Leeds Institute of Health Sciences<br>Graham, Christopher D.; Queens University Belfast<br>Mason, Ellen; University of Leeds Leeds Institute of Clinical Trials Research<br>French, David; University of Manchester, School of Psychological Sciences<br>Hall, Louise; University of Leeds, Institute of Health Sciences<br>Wilkes, Hollie; University of Leeds, Clinical Trials Research Unit<br>McNaught, Emma; University of Leeds, Clinical Trials Research Unit<br>Raine, Erin; Leeds Institute of Health Science University of Leeds<br>Walwyn, Rebecca; University of Leeds, Clinical Trials Research Unit<br>Howdon, Daniel; University of Leeds Leeds Institute of Health Sciences, Academic Unit of Health Economics<br>Clark, Jane; Leeds Teaching Hospitals NHS Trust, Department of Clinical and Health Psychology<br>Rousseau, Nikki; University of Leeds, Surgical, Diagnostic and Devices Division<br>Buxton, Jacqueline; University of Leeds<br>Moore, Sally J. L.; University of Leeds<br>Parbutt, Catherine; Leeds Teaching Hospitals NHS Trust<br>Velikova, Galina; University of Leeds, Leeds Institute of Medical Research<br>Farrin, Amanda; University of Leeds, Clinical Trials Research Unit<br>Collinson, Michelle; University of Leeds Clinical Trials Research Unit
**Primary Subject Heading**:	Oncology
Secondary Subject Heading:	Health services research
Keywords:	Breast tumours < ONCOLOGY, PREVENTIVE MEDICINE, PUBLIC HEALTH

Refining and Optimising a behavioural intervention to Support Endocrine Therapy Adherence (ROSETA) in UK women with breast cancer: protocol for a pilot fractional factorial trial

Samuel G. Smith[1], Sophie M. C. Green[1], Rachel Ellison[2], Robbie Foy[1], Christopher D. Graham[3], Ellen Mason[2], David P. French[4], Louise H. Hall[1], Hollie Wilkes[2], Emma McNaught[2], Erin Raine[1], Rebecca Walwyn[2], Daniel Howdon[1], Jane Clark[5], Nikki Rousseau[2], Jacqueline Buxton[6], Sally J. L. Moore[6], Catherine Parbutt[7], Galina Velikova[8], Amanda Farrin[2], Michelle Collinson[2]

1. Leeds Institute of Health Science, University of Leeds
2. Clinical Trials Research Unit, Leeds Institute of Clinical Trials Research, University of Leeds
3. School of Psychology, Queens University Belfast
4. Division of Psychology and Mental Health, University of Manchester
5. Department of Clinical and Health Psychology, Leeds Teaching Hospitals NHS Trust
6. Independent
7. Medicines Management and Pharmacy Services, Leeds Teaching Hospitals NHS Trust
8. Leeds Institute for Medical Research, University of Leeds

Corresponding author:

Dr Samuel Smith

E: s.smith1@leeds.ac.uk

T: 0113 343 0892

Words (abstract): 297/350

Words (article): 4045/4000

For peer review only

Abstract

Introduction: Women with breast cancer who do not adhere to adjuvant endocrine therapy (AET) have increased risks of mortality and recurrence. There are multiple barriers to AET adherence, including medication side-effects, beliefs about medication, memory, and psychological distress. We developed four intervention components, each targeting a different barrier. This pilot trial is part of the preparation phase of the Multiphase Optimisation Strategy (MOST), and aims to establish key trial parameters, establish intervention component adherence, establish availability and feasibility of outcome and process data, estimate variability in planned outcome measures, and estimate cost of developing and delivering each intervention component.

Methods and analysis: The four intervention components are: SMS text reminders (target: memory); a written information leaflet (target: medication beliefs); a guided self-help Acceptance and Commitment Therapy programme (target: psychological flexibility to reduce distress); a self-management website (target: side-effect management). To evaluate the feasibility of recruitment, acceptability of the intervention components and the availability of outcome data, we will conduct a multi-site, exploratory pilot trial using a 2^{4-1} fractional factorial design, with a nested process evaluation. We will randomise 80 women with early-stage breast cancer who have been prescribed AET to one of eight experimental conditions. This will determine the combination of intervention components they receive, ranging from zero to four, with all conditions receiving usual care. Key outcomes of interest include medication adherence and quality of life. Progression to the optimisation phase will be based on pre-defined criteria for consent rates, patient adherence to intervention components and availability of medication adherence data.

Ethics and dissemination: The study was reviewed by the Wales Research Authority Research Ethics Committee 3 (21/WA/0322). Written informed consent will be obtained from all patients before randomisation. The results of this trial will be disseminated in a peer-reviewed journal.

Trial registration: ISRCTN: 10487576 (16/12/2021)

Keywords: Medication adherence; breast cancer; Multiphase Optimisation Strategy (MOST); Acceptance and Commitment Therapy; SMS reminders; information leaflet, self-management website, pilot; feasibility; quality of life; factorial

For peer review only - http://bmjopen.bmj.com/site/about/guidelines.xhtml

Strengths and limitations of this study

- This design will estimate the individual and combined effects of multiple intervention components
- This multi-site pilot will recruit women through 3 routes
- Progression to an optimisation trial will be decided using a priori criteria
- A mixed-methods process evaluation will assess intervention acceptability and fidelity
- The pilot will not examine effectiveness of the intervention components

For peer review only

For peer review only - http://bmjopen.bmj.com/site/about/guidelines.xhtml

Introduction

Approximately 55,000 UK women are diagnosed with breast cancer each year, with 11,000 cancer-related deaths [1]. Most breast cancers are oestrogen receptor positive (ER+) tumours. Adjuvant endocrine therapies (AETs) such as selective oestrogen receptor modulators (SERMs e.g. tamoxifen) and aromatase inhibitors (AIs e.g. Letrozole) improve outcomes for women with ER+ tumours. Low adherence to AET is linked with higher rates of recurrence and all-cause mortality [2–9]. A meta-analysis reported 31% to 73% of breast cancer survivors are adherent to AET [10], and stopping AET is most common within 12 months of initiation [11–13]. Unintentional non-adherence, such as forgetting, may be more common than intentionally missing doses [14–16].

An overview of behavioural support programmes for women using AET reported at least 15 ongoing trials targeting AET adherence [17]. Among adequately powered trials of AET interventions there are few positive findings [18–20]. This may be explained by the reliance on interventions involving the provision of written information; which may address some adherence barriers, but is unlikely to address the complex issues underpinning most medication taking behaviours [17,21]. As with many long-term conditions where treatment non-adherence is a problem, intervention programmes may require multiple active components to target a range of key determinants.

Existing AET adherence trials have used single-arm or parallel group randomised designs. While parallel group trials are suitable for definitively evaluating a complex intervention against a suitable comparator, they provide little information on the relative contributions of individual components [22]. Undertaking separate or multi-arm randomised controlled trials (RCTs) to evaluate individual intervention components would be prohibitively expensive and time-consuming, and would not allow estimates of interactions between intervention components. Alternative trial designs are needed to improve our understanding of complex interventions, and increase the efficiency of definitive evaluations.

The Multiphase Optimisation Strategy (MOST) is a framework that includes an optimisation phase between developing and evaluating a complex intervention [22]. Within this optimisation phase, MOST advocates the use of highly efficient experimental designs to provide empirical data on the main effects and interactions of intervention components. This information is used to identify the optimal combination of intervention components to produce the desired outcome, without exceeding key constraints (e.g. cost) [23]. The optimised intervention package can be evaluated in a definitive RCT. The process of optimisation aims to yield more effective, affordable, scalable and efficient

For peer review only

intervention packages compared with alternative approaches [24,25]. Given the complexity of adherence behaviours, the lack of information available about individual intervention component efficacy, and the need to address an important clinical need efficiently, we used the MOST framework to guide our approach to intervention optimisation.

As part of the preparatory phase of MOST, we used Intervention Mapping to develop a theoretically informed intervention package [17,26]. We identified several modifiable factors associated with AET adherence [27–30], selecting four of the strongest determinants as intervention targets [17]. Four intervention components were developed or adapted to address these targets. The current trial represents the next step in the preparation phase of MOST; examining the feasibility of an optimisation trial of the proposed intervention package in women with early-stage breast cancer. Our objectives are to: i) establish eligibility, recruitment, retention and follow-up rates; ii) establish intervention component adherence; iii) establish availability and feasibility of outcome and process data; iv) estimate variability of planned outcome measure(s); and v) estimate cost of developing and delivering each intervention component.

We will also include a nested process evaluation to further inform the decision of whether to undertake a future optimisation trial, and how the intervention components could be adapted based on intervention fidelity and acceptability, and trial experience. A full protocol of this process evaluation is published elsewhere [31].

Methods and analysis

Design

This is a multi-site, exploratory pilot trial using a 2^{4-1} fractional factorial design with a nested mixed methods process evaluation. We will randomise 80 women with early-stage breast cancer to one of eight experimental conditions, which will determine the intervention components they receive (Table 1). There will be an equal chance of allocation to each group. Each component has two levels (present or absent). All women will receive usual care. Women will be followed-up at 2 and 4-months, and key outcomes include medication adherence (self-report and NHS data) and quality of life. The trial adheres to the SPIRIT recommendations [32] (Appendix 1), and the CONSORT extension for pilot and feasibility trials [33] (Appendix 2) and the intervention components are described using the TIDieR checklist [34] (Appendix 3).

For peer review only - http://bmjopen.bmj.com/site/about/guidelines.xhtml

Table 1. 2^{4-1} fractional factorial design for pilot trial

Condition	Usual Care	Text reminders	Information leaflet	Acceptance & Commitment Therapy	Side-effect website	Randomisation sample size
**1**	Yes	Yes	Yes	Yes	Yes	10
**2**	Yes	Yes	Yes	No	No	10
**3**	Yes	Yes	No	Yes	No	10
**4**	Yes	Yes	No	No	Yes	10
**5**	Yes	No	Yes	Yes	No	10
**6**	Yes	No	Yes	No	Yes	10
**7**	Yes	No	No	Yes	Yes	10
**8**	Yes	No	No	No	No	10

For peer review only

Setting

Participant identification and recruitment will occur at five UK NHS hospitals, detailed in the ISRCTN registry (ISRCTN10487576). Sites with existing or planned implementation of interventions designed to improve adherence to AET will be excluded. The site must have access to a Health and Care Professional Council (HCPC) registered practitioner psychologist (Clinical, Health or Counselling Psychologist) or a UK Council for Psychotherapy (UKCP) registered psychotherapist. The site must have access to video conferencing software or a telephone to deliver the ACT sessions.

Participants

Adult women using tamoxifen, raloxifene, anastrozole, letrozole, or exemestane as AET for early-stage (1 to 3a) breast cancer are eligible (Table 2).

Table 2. Eligibility criteria for participation in the ROSETA pilot trial

**Inclusion Criteria**	**Exclusion Criteria**
1. An informed consent form (signed and dated)<br>2. Capacity to provide informed consent<br>3. Women with early stage (1 to 3a) breast cancer according to the TNM / American Joint Committee on Cancer (AJCC) staging system.<br>*Note. Women being treated for a second primary breast cancer or a breast cancer local recurrence are eligible for the study, providing the most recent cancer is being treated with adjuvant endocrine therapy, and they meet all eligibility criteria. Women with bilateral breast cancer are permitted, providing at least one breast is affected by hormone receptor-positive disease'*<br>4. Aged ≥18 years at time of screening for ROSETA's pilot study	1. Stopped taking adjuvant hormone therapy if it is clinically contraindicated according to clinical recommendation<br>2. Women with Metastatic breast cancer<br>3. Currently or recently (last 6 months) involved in a similar research study where medication adherence is a primary outcome*<br>4. Currently attending psychotherapy/psycho-oncology/psychology/counselling services, for any clinical reason*<br>5. Need for treatment for a severe mental health disorder or crisis, which is likely to interfere with participation (e.g., active psychosis, bipolar disorder, significant issues with addiction or self-harm or expressing

For peer review only - http://bmjopen.bmj.com/site/about/guidelines.xhtml

5. Have sufficient proficiency in English to be able to adhere to all intervention components and data collection required<br>6. Treated with curative intent<br>7. Completed their hospital-based treatment (e.g., surgery, radiotherapy and/or chemotherapy) for the current breast cancer within the last 12 months.<br>*Note. Women are still eligible for the study if they are being treated with monoclonal antibody-based therapy such as trastuzumab, kadcyla, pertuzumab, and phesgo*<br>8. Currently prescribed oral adjuvant Hormone Therapy (tamoxifen, raloxifene, anastrozole, letrozole, exemestane)<br>9. The participant is willing to complete the study questionnaires*<br>10. The participant is willing to be audio recorded during the therapy sessions*<br>11. The participant is willing and able to attend all ACT sessions either via video conference or telephone*<br>12. The participant is willing and able to complete home practice tasks*<br>13. Access to a mobile phone to receive SMS messages*<br>14. Willing to receive frequent SMS messages*<br>15. Access to a computer or smart device that can access the internet*	active suicidal ideation with active plans and intent*)<br>*Note, if concerned about the possible presence of risk of suicidal ideation with active plans and intent, then this can be assessed with the following questions, with patients ineligible if they answer 'yes' to 5c.*<br><br>*Recently (in the last month):*<br>*a. Have you had any thoughts about ending your life?*<br>*b. (if yes) Have you thought about how you might go about it?*<br>*c. (if yes) Do you intend to carry out this plan?*<br><br>6. Patients with a scheduled date for breast reconstruction surgery that is within their intervention delivery and follow-up period. *Note: Women planning to have a breast reconstruction but who have not scheduled a date for surgery are permitted*<br><br>7. Auditory problems that would prevent the patient from participating in a telephone or video call, or hearing audio clips*

*Source data for these items will be either partially or completely patient self-report.

Trial Processes

Participants will be identified via three routes. Route 1 involves the research nurse (RN) prospectively screening patient records prior to clinic visits. Route 2 will identify patients who have self-referred to their care team to discuss problems with their medications. Route 3 involves the RN retrospectively searching patient records to identify patients who have completed hospital treatment. All patients should have completed their last hospital treatment within 12 months of randomisation.

Sites will complete a screening form for all identified patients. Anonymised data on age, ethnicity, staging, tumour type and whether a patient is randomised will be captured. Patients who are not randomised will have the reason they are ineligible or declined participation recorded.

For peer review only

For peer review only - http://bmjopen.bmj.com/site/about/guidelines.xhtml

For recruitment route 1 a member of the care team will introduce a RN to the patient, and offer the option of receiving trial documents by email. Eligible patients may consent immediately or after further consideration. In route 2, the patient's oncologist will conduct an initial review of eligibility and, where participants are willing, provide their contact details to a RN who will telephone them and email trial documents. Interested patients contact the RN by telephone and consent is taken. For route 3 the RN will post or email the patient an invitation letter and a copy of the patient information sheet. For all routes, a RN will confirm eligibility and record consent (Appendix 4). Eligibility and consent discussions, and completion of baseline measures may take place in person or remotely.

Authorised individuals at NHS sites will be given access to the Clinical Trials Research Unit (CTRU) online database and will enter site and participant details, and confirm eligibility, and informed consent. After recording consent, the RN will register the participant and a link to the baseline questionnaire will be sent automatically from the CTRU. Once completed, the RN will notify the CTRU that a participant can be randomised, and an authorised member of the CTRU will perform the randomisation. Participants will be randomised to one of eight experimental conditions (Table 1). Stratified permuted block randomisation will ensure experimental conditions are well-balanced for the recruitment route. Participants, therapists, RNs, participants' GPs and CTRU staff (including the Chief Investigator) will not be blinded to the randomised allocation. The RN will notify participants of their allocation.

We will ask participants to complete electronic follow-up questionnaires at 2 and 4-months post-randomisation. Non-responders will receive telephone, email and/or SMS message reminders.

Patients may withdraw from the SMS and ACT intervention components, completing the questionnaires, or from the collection of data from NHS Digital and data processing. The local principal investigator may decide a participant should be withdrawn if they have become unsuitable for the trial. The analysis will use data collected up to the date of withdrawal of consent.

Usual Care

All participants will receive treatment as usual, which will be the standard care offered to women at this stage of their breast cancer treatment. It is likely to differ by recruiting site. Most women will be invited to attend an end of treatment summary meeting with a breast care nurse. The content of treatment as usual programmes and information on hospital-based services accessed will be reported through patient self-report and site-level report. Providers of usual care will not be blinded.

For peer review only - http://bmjopen.bmj.com/site/about/guidelines.xhtml

For peer review only

Intervention components

SMS Intervention Component

Memory problems are common in women with breast cancer [35,36], and forgetting is a cause of non-adherence to AET [14,15,37,38]. Forming medication taking habits can reduce reliance on memory and help sustain behaviour change [39–42]. Mobile phone messaging interventions are a potential cost-effective approach to promote habit formation [43–45]. This has not been widely tested in cancer patients [46].

We co-developed, with behaviour change experts and women affected by breast cancer, an intervention component involving SMS reminders to support habit formation of daily medication taking and associated behaviours (e.g., ordering prescriptions) [47]. The SMS messages are based on six behaviour change techniques theorised to support the development of habitual behaviours [48]. The SMS messages will be sent by CTRU and delivered over four months, commencing up to one week following randomisation. Forty-three SMS messages will be delivered to participants in the same order; three opening messages, daily messages for 2 weeks, two messages per week for 8 weeks, weekly messages for 6 weeks, and one closing message. A message will be sent monthly informing participants they can stop the SMS messages by emailing the trial team.

Medication Beliefs Intervention Component

Women using AET report low perceived need for therapy, while also citing unfounded concerns about the medication, which could impact adherence [28,29,49–52]. Qualitative studies have highlighted a demand for accurate information about AET to overcome unfounded concerns [27–29].

We developed a 6-page patient information leaflet to target AET medication beliefs. The leaflet explains how AET works supplemented by diagrams, information about AET benefits and side effects, answers to common concerns, and quotes and photos of breast cancer survivors. Leaflet content is informed by existing qualitative research, views from our patient and public involvement group, the Necessity Concerns Framework [52] and the Common-Sense Model of Self-regulation [53]. Participants receiving this component will be emailed the leaflet by the NHS site, after randomisation.

Acceptance and Commitment Therapy (ACT) Intervention Component

Approximately half of women with breast cancer experience higher distress than the general population [30,54,55]. Distress is associated with adherence to AET [30,56], and interventions

For peer review only - http://bmjopen.bmj.com/site/about/guidelines.xhtml

targeting this barrier could support medication adherence behaviours. Any prospective intervention would need to target the factors contributing to distress, including fear of recurrence, difficulties 'returning to normal', and distress caused by side-effects [27,57,58].

The ACT component is a guided self-help programme targeting psychological distress, by promoting psychological flexibility [59,60]. We used an adapted version of a guided self-help ACT intervention shown to be effective for reducing distress in people with muscle diseases [61]. It consists of five telephone or video call sessions with a therapist. The modules suggest four ACT-based skills; mindfulness, unhooking, following values and living beyond labels, that aim to enhance well-being and reduce distress. Each module consists of a participant manual containing information about an ACT skill, alongside home practice exercises and audio files.

The participant will see the same therapist for each session. The first session will take place within 4 weeks of randomisation and consists of a 15-minute introduction. The three weekly sessions with the therapist will last 25 minutes each. Within these sessions, the therapist and the patient will discuss the previous week's module, and reflect on the participant's experience of using the skills. Following each session, the therapist will email the patient the next module's materials. One week after the fourth module there will be a closing 15-minute support session. Participants allocated to receive ACT can cease participation in that component by informing the therapist or contacting the trial team.

Self-management Website Intervention Component

AET side effects (e.g. hot flushes and arthralgia [62–67]) reduce quality of life [62,68–72]. Side-effects also likely impact medication adherence [68,73–75], although evidence is mixed [76]. Oncologists perceive side-effects to be a major deterrent to AET adherence [77], which is corroborated by patients [27,78]. Women feel unsupported in managing side-effects [27,57,78], and would like clearer information on self-management strategies [58,79]. We undertook an umbrella review of systematic reviews and clinical guidelines on self-management strategies for common AET side-effects [80]. This review informed the creation of a patient-facing website to support side-effect management in women using AET.

Website creation was also based on suggestions made by patients and healthcare professionals attending a co-development workshop [81]. The website includes videos of patient stories, sign-posting for further information, and information on common AET side-effects. Participants

For peer review only - http://bmjopen.bmj.com/site/about/guidelines.xhtml

randomised to the website component will be sent login details by the NHS site following randomisation.

Measures

The primary outcomes for the study are the progression criteria (consent rate, component adherence, and medication adherence measures). Secondary outcomes include quality of life, costs, psychological flexibility, beliefs about medications, habit formation, psychological distress, safety, acceptability, trial experience, and fidelity. Table 3 and Table 4 summarise timing of data collection for each assessment.

Participant Measures

At baseline, we will collect data on name, postcode, NHS number, email address, telephone number, date of birth, gender, marital status, employment, education, menopausal status, year of diagnosis, stage of cancer at diagnosis, tumour type, breast cancer treatment received, co-morbidities, AET regimen, supportive therapies used following AET prescription, and previous exposure to ACT, Cognitive Behavioural Therapy and mindfulness.

We will obtain participant consent to apply to NHS Digital for participant-level AET prescribing or dispensing data.

The following patient-reported measures will be used:

Morisky Medication Adherence Scale (MMAS-8) [82]

An 8-item, patient report measure for assessing medication adherence. It provides an overall adherence score, as well as a score for intentional and non-intentional non-adherence.

Voils DOSE-Nonadherence measure- Extent Scale [83]

A 3-item patient report scale to assess the extent of medication adherence.

European Organisation for Research and Treatment of Cancer (EORTC) QLQ-C30 [84]

A 30-item, patient report tool to assess health-related quality of life in cancer patients.

EORTC QLQ-BR45 [85]

A 45-item self-report measure for assessing quality of life in patients with breast cancer.

EORTC-IL133

For peer review only - http://bmjopen.bmj.com/site/about/guidelines.xhtml

Two items assessing vaginal discharge and abnormal vaginal bleeding, taken from the EORTC item library.

For peer review only

Table 3. Summary of assessments

Assessment	Source	Method of Completion	TIMELINE				
			Screening	Baseline	2 Months	4 Months	End of Trial
**PARTICIPANT**							
Screening	Screening CRF	Research Nurse/Delegate	X				
Contact Details	CRF	Research Nurse/Delegate		X	CTRU to be notified of changes		
Demographics including comorbidities (Charlson Comorbidity Index)	CRF	Self-completion/Research Nurse/Delegate		X			
Eligibility (including Inclusion and Exclusion Criteria)	CRF	PI/Research Nurse/PIs Delegate		X			
Consent	Consent Form	Self-completion / RN/Delegate over the telephone		X			
Randomisation	CRF/CTRU Online system	Research Nurse/Delegate		X			
Prescribing/Dispensing Data	NHS Digital	PIs Delegate		X	X	X	
MMAS-8	Questionnaire	Self-completion		X	X	X	
Voils DOSE-Nonadherence measure- Extent Scale	Questionnaire	Self-completion		X	X	X	
EORTC QLQ-C30	Questionnaire	Self-completion		X	X	X	
EORTC QLQ-BR45	Questionnaire	Self-completion		X	X	X	
EORTC-IL133	Questionnaire	Self-completion		X	X	X	
EQ-5D-5L	Questionnaire	Self-completion		X	X	X	
Intervention costs	NHS Reference Costs/ PSSRU	PIs Delegate				X	
MPFI (short form)	Questionnaire	Self-completion		X	X	X	
BMQ-AET	Questionnaire	Self-completion		X	X	X	
SRBAI	Questionnaire	Self-completion		X	X	X	
DASS-21	Questionnaire	Self-completion		X	X	X	
McGill QoL Revised	Questionnaire	Self-completion		X	X	X	
Safety Reporting	CRF	Research Nurse/PI/Delegate			X		
End of Trial Data	CRF	Research Nurse					X
End of Trial Site Data	CRF	Research Nurse					X
SMS Delivery and receipt Data	Online System	Delegate			X	X	
Opt-out of SMS messages	Online system	Delegate			X (collected throughout)		
Information leaflet delivery	Site recorded	Research Nurse		X			
ACT session attendance, number of cancelled/ missed sessions	CRF	Clinician			X (collected throughout)		

For peer review only

Engagement with ACT module materials (participant manual, associated audio files and home practice tasks)	CRF	Clinician		X (collected after each therapy session)			
Engagement with ACT module audio files	Questionnaire	Self-completion				X	
Completion of ACT home practice tasks	Questionnaire	Self-completion				X	
Dates of ACT sessions	CRF	Clinician		X (collected throughout)			
Printing of ACT module booklets	Questionnaire	Self-completion				X	
Delivery of website login details	Site recorded	Research Nurse		X			
Website usage	Website Online System	Delegate			X	X	
Self-reported receipt of SMS, information leaflet, ACT modules and website	Questionnaire	Self-completion				X	
Self-reported reading of SMS, information leaflet, ACT module participant manuals and website	Questionnaire	Self-completion				X	
Acceptability Questionnaire	Questionnaire	Self-completion				X	
Questionnaire return	Online system	Delegate		X	X	X	
Trial withdrawals	CRF	Delegate	X (collected throughout)				
Study Participant Feedback Questionnaire (SPFQ)	Questionnaire	Self-completion		X	X	X	
Fidelity of receipt and enactment of intervention components	Semi-structured interview	Interview				X	X
Barriers and facilitators to trial participation and response rates	Semi-structured interview	Interview					X
Barriers and facilitators to recruitment	Questionnaire	Research Nurse Self-completion					X
Research Nurse Demographics	CRF	Research Nurse		X			
UK Cancer Costs Questionnaire	Questionnaire	Self-completion			X	X	
Questionnaire reminder preference	Questionnaire	Self-completion				X	

For peer review only

For peer review only

For peer review only - http://bmjopen.bmj.com/site/about/guidelines.xhtml

Table 4. Summary of ACT therapist assessments

**Assessment**	**Source**	**Method of Completion**	**Post-therapist training**	**Pre-trial delivery**	**1st session**	**2nd session**	**3rd Session**	**4th Session**	**5th Session**	**End of trial**
Consent	Consent form	Therapist	X							
Demographics	CRF	Therapist	X							
ACT-FM Competency Assessment (Therapist stance subscale)	Questionnaire	ACT trainer			X					
Number of booster training sessions required	Questionnaire	ACT trainer		X (collected throughout)						
ACT-FM therapist stance subscale	Questionnaire	Independent Expert Reviewer								X
Procedural Fidelity Checklist	CRF	Therapist			X	X	X	X	X	
Fidelity of training and delivery	Semi-structured interview	Interview								X

For peer review only

EQ-5D-5L [86]

A 5-item, self-assessed, health-related quality of life questionnaire.

Multi-Dimensional Psychological Flexibility Inventory-Short form [87]

A 24-item self-report questionnaire assessing psychological flexibility and inflexibility.

Beliefs about Medication Questionnaire (BMQ-AET) [88]

The BMQ-AET is a modified version of the BMQ to increase its relevance to women taking AET. It is a 10-item patient report measure which assesses specific medication beliefs.

Self-Report Behavioural Automaticity Index (SRBAI) [89]

A 4-item self-report tool that captures habitual behaviour patterns, specifically with regard to automaticity of the behaviour.

Depression Anxiety Stress Scales (DASS)-21 [90]

A 21-item self-report measure of negative emotional states.

McGill Quality of Life-Revised [91]

A 14-item, patient-report tool designed to measure physical well-being, physical and psychological symptoms, existential well-being and support, as well as overall quality of life of people with life-threatening illness.

UK Cancer Cost Questionnaire [92]

An adapted version of the UK cancer cost questionnaire. The questionnaire assesses services and medications participants have used while participating in the trial.

Site measures

Usual Care Assessment

Each site will record what services are offered as part of usual care.

Health economic assessments

Intervention costs

For peer review only - http://bmjopen.bmj.com/site/about/guidelines.xhtml

For peer review only

We will estimate the cost of developing each intervention component. We will use NHS Reference Costs and Personal Social Sciences Research Unit costs to estimate delivery costs for the ACT component.

Process evaluation measures

Adherence to intervention components

To assess fidelity of receipt of the intervention components, participants will be asked whether they received each of the intervention components they were randomised to, and how much of it they read. Participants who received the ACT component will additionally be asked about how much of the home practice tasks they completed, and how many of the audio files they listened to.

Acceptability Questionnaire (AQ) [93]

A 5-item self-report measure assessing acceptability. Participants randomised to the SMS component will be asked an additional item about acceptability of the frequency of the SMS messages. Participants randomised to the ACT component will be asked an additional 15 items about the acceptability of elements of ACT sessions. Participants will be provided with an open text question about acceptability. A single item will additionally ask about overall trial acceptability.

Study Participant Feedback Questionnaire (SPFQ) [94]

A modified version of the SPFQ assessing participants' experience during the trial will include 2, 3, and 2 items at baseline, 2-month and 4-month follow-ups, respectively.

ACT Fidelity Measure (ACT-FM) (therapist subscale) [95]

A 7-item subscale from a measure of fidelity to the principles of ACT will be scored by an expert in ACT while reviewing therapy recordings. This subscale includes items measuring pre- scribed (4 items) and pro-scribed (3 items) therapist behaviours.

Procedural Fidelity Checklist

A checklist to assess fidelity of delivery of the ACT component recorded by the therapist at the end of each session to self-rate whether they undertook crucial intervention procedures. It includes 4 to 8 items, depending on the session. Additional items will be used for therapists to record participant engagement with the module materials.

Research Nurse Questionnaire

For peer review only - http://bmjopen.bmj.com/site/about/guidelines.xhtml

A 9-item bespoke questionnaire to report RN experiences of recruiting to a fractional factorial trial. Demographic and role information will also be collected for the RNs.

Process evaluation

Fidelity of design

Two independent coders will identify and record which BCTs from the BCT Taxonomy v1 are present in each intervention component [48].

Fidelity of training

Each therapist's first ACT session will be assessed for competency using the ACT-FM by a clinically trained member of the research team (CG).

Fidelity of delivery

We will collect SMS receipt data, record the number of information leaflets and website details sent to participants, use independently rated competency scores on the ACT-FM therapist stance subscale, and report scores on the procedural fidelity checklist.

Fidelity of receipt

We will use self-reported data on receipt and reading of the materials within an intervention component, SMS opt outs, website tracking data, therapist-reported attendance and engagement with ACT session, and independently assessed engagement with ACT session.

Participant and therapist interviews

Semi-structured interviews with participants will investigate the acceptability, and fidelity of receipt and enactment of the intervention components. ACT therapists will be interviewed, to gain insight into their experiences of intervention delivery, and the acceptability of the ACT component. Interviews lasting approximately one hour will be conducted via video conferencing software or telephone.

Sample Size

The primary objective is to estimate rates of eligibility, recruitment, retention, and component adherence to inform the future trial. Allowing for 80% retention (the average across 8 studies [20]), 80 participants (n=10 per condition) will be randomised. This is sufficient to inform the sample size of the optimisation trial assuming adherence data are pooled across conditions [96].

For peer review only - http://bmjopen.bmj.com/site/about/guidelines.xhtml

Patient and Public Involvement

A user group of 5 breast cancer survivors have supported the development of the intervention components and trial protocol. Two representatives are members of the trial management group.

Independent Monitoring

The Trial Steering Committee (TSC), with an independent Chair, will be responsible for trial oversight. The TSC will be provided with reports prepared by the CTRU based on an agreed TSC Charter and containing the information agreed in the trial monitoring plan. It includes an Independent Chair, at least two other independent members, and two patient representatives. The Chief Investigator and other members of the TMG may attend the TSC meetings and report progress. A separate data monitoring committee was considered by the TSC to be unnecessary. The TSC will adopt a safety function.

Data Management

Data collection forms transferred to or from the CTRU will be coded with a study number (made up of the recruitment site code and the participant's unique sequential trial number), the participant's initials, and date of birth. Study data will be held securely on paper and electronically at the University of Leeds CTRU, and appropriate processes put in place for the transfer, storage, restricted access, and disposal of personal information. Relevant standard operating procedures, guidelines and work instructions in relation to data management, processing, and analysis of data will be followed.

Analysis

A detailed statistical analysis plan will be written and signed off before analysis is undertaken. The analysis will focus on descriptive statistics and confidence interval estimation rather than formal hypothesis testing. All analyses will be undertaken on the intention-to-treat population, with all participants included in the analysis according to their randomised allocation, and regardless of non-adherence to the intervention components or withdrawal from the trial. Final analysis will be conducted once all available outcome data is received. Outcome measures will be scored according to relevant scoring manuals. Pre-defined progression criteria will be used to judge the feasibility of progressing to the optimisation trial (Table 5). Proof of principle will be explored via investigation of between-group change in outcomes. Point estimates and 95% confidence intervals will be presented for the main effects and interaction effects for adherence and quality of life outcomes. Point estimates and 95% confidence intervals for process variables will be presented for the intervention components in which a change is hypothesised. Analysis will adjust for the stratification factor.

For peer review only - http://bmjopen.bmj.com/site/about/guidelines.xhtml

Table 5 Progression criteria for deciding whether or not to proceed to the optimisation trial

	Green	Amber	Red
Eligible patients consent rate	≥30%	≥10%	<10%
Component adherence			
75% of SMS messages received with no opt out	≥50%	≥20%	<20%
Read 'at least some' of the information leaflet	≥50%	≥20%	<20%
Completed 2/4 ACT modules	≥50%	≥20%	<20%
Registered and logged onto website at least once	≥50%	≥20%	<20%
Availability of adherence measures with ≥75% complete data	≥2	≥1	0

Green (go): optimisation phase is feasible with no changes to design or procedures; Amber (modify): optimisation phase is feasible following minor enhancement of procedures; Red (stop): optimisation phase is not feasible.

A rapid evaluation approach will be used for the qualitative interviews [97]. Rapid Research Evaluation and Appraisal Lab (RREAL) sheets, which are summary tables collected after an interview, will be created for each individual participant, and collated into higher level summary sheets [98]. Regular team meetings will discuss emerging findings, changes to the interview schedule, and data saturation. The interviews will be recorded. The RREAL sheets will be used to guide analysis, and illustrative quotes used to support themes.

Recruitment status

The trial opened for recruitment on the 20th May 2022, and closed to recruitment on the 16th December 2022.

<u>Ethics and dissemination</u>

Ethical approval and data

The trial has been approved by Wales Research Authority Research Ethics Committee 3 (21/WA/0322) and is a registered clinical trial (ISRCTN10487576). It is sponsored by the University of Leeds (governance-ethics@leeds.ac.uk). Amendments to the protocol will be submitted to the ethics committee, and if approved, communicated to research sites. Trial findings will be disseminated through peer-reviewed publications. At the end of the trial, all data held by the CTRU and all trial data will then be securely archived at the University of Leeds for a minimum of 5 years. This paper is a

For peer review only - http://bmjopen.bmj.com/site/about/guidelines.xhtml

summary of protocol version 3.0 (18/08/2022), available upon request from the corresponding author.

Dissemination

Results will be presented at scientific meetings and published in international peer-reviewed journals. Authorship decisions will be guided by the International Committee of Medical Journal Editors criteria. Summaries will be provided to participants and the trial funder.

Safety

We expect episodes of acute illness, infection, new medical problems and deterioration of existing medical problems will occur and could result in prolonged hospitalisation, hospital re-admission, significant or permanent disability or incapacity, or death. In recognition of this, events fulfilling the definition of a serious adverse event (SAE) will not be reported unless the event resulted from administration of any research procedure, and fulfils the definition of a related and unexpected serious adverse event (RUSAE). Reports of physical self-harm will also be considered Serious Adverse Events and assessed for relatedness and unexpectedness. We might anticipate the following adverse events related to the intervention; low mood (including suicidal thoughts and plans), fatigue, anxiety and/or psychological distress. Adverse events relating to these factors will not be reported as a RUSAE.

Safety will be monitored through observed increases in 'extremely severe' DASS-21 scores for anxiety, stress and depression, a planned RN phone call at 4 months, ad-hoc RN phone calls if a participant wishes to disclose a mental health crisis or referral, disclosures within the semi-structured interviews and ACT sessions. Routinely collected hospital data will be used to record deaths, hospitalisations and mental health crisis referrals.

Access to data

Data will only be shared for participants who have given consent to use of their data for secondary research, and will only be made available in such a way that recipients cannot identify individuals by any reasonable likely means. We will only share data for projects that have ethical approval granted, are clearly in the public interest and compatible with the original purpose of the data processing. Requests to access trial data should be made to CTRU-DataAccess@leeds.ac.uk in the first instance.

Author contributions

For peer review only - http://bmjopen.bmj.com/site/about/guidelines.xhtml

We followed the ICTMC guidelines for authorship. Planning for the research, including the trial concept and trial design, was undertaken by: SGS, SMCG, RE, RF, CDG, EM, DPF, LHH, HW, EM, ER, RW, DH, JC, NR, JB, SJLM, CP, GV, AF, MC. The first draft of the paper was prepared by SGS, with important intellectual content revisions made by SMCG, RE, RF, CDG, EM, DPF, LHH, HW, EM, ER, RW, DH, JC, NR, JB, SJLM, CP, GV, AF, MC. Final approval for the manuscript was provided by SGS, SMCG, RE, RF, CDG, EM, DPF, LHH, HW, EM, ER, RW, DH, JC, NR, JB, SJLM, CP, GV, AF, MC. The conduct of the trial is ongoing, and therefore we are not reporting data, analysis or interpretation. SGS is the guarantor.

Funding

This trial is funded by the National Institute for Health and Social Care Research (NIHR300588) (PI: Samuel Smith). Smith is also supported by a Yorkshire Cancer Research Fellowship (L417). The funder(s) had no role in the design of the trial, data collection, analysis, interpretation of data, and in the writing of this manuscript. RF is supported by grant funding from the National Institute of Health and Social Care Research (N/A) and Yorkshire Cancer Research (N/A). DF is supported by the Manchester Biomedical Research Centre (IS-BRC-1215-20007).

Competing interest

The authors declare no competing interests.

Acknowledgements

We would like to thank our patient and public involvement members; Rebecca Whittemore, Michaela Kneafsey and Hazel Abey. We also thank the Trial Steering Committee for their advice in designing and delivering the trial.

For peer review only

References

1 Smittenaar CR, Petersen KA, Stewart K, *et al.* Cancer incidence and mortality projections in the UK until 2035. *Br J Cancer* 2016;**115**:1147–55. doi:10.1038/bjc.2016.304

2 Chirgwin JH, Giobbie-Hurder A, Coates AS, *et al.* Treatment Adherence and Its Impact on Disease-Free Survival in the Breast International Group 1-98 Trial of Tamoxifen and Letrozole, Alone and in Sequence. *J Clin Oncol* 2016;**34**:2452–9. doi:10.1200/JCO.2015.63.8619

3 Dezentjé VO, van Blijderveen NJC, Gelderblom H, *et al.* Effect of concomitant CYP2D6 inhibitor use and tamoxifen adherence on breast cancer recurrence in early-stage breast cancer. *J Clin Oncol* 2010;**28**:2423–9. doi:10.1200/JCO.2009.25.0894

4 Ma AMT, Barone J, Wallis AE, *et al.* Noncompliance with adjuvant radiation, chemotherapy, or hormonal therapy in breast cancer patients. *Am J Surg* 2008;**196**:500–4. doi:10.1016/j.amjsurg.2008.06.027

5 Makubate B, Donnan PT, Dewar JA, *et al.* Cohort study of adherence to adjuvant endocrine therapy, breast cancer recurrence and mortality. *Br J Cancer* 2013;**108**:1515–24. doi:10.1038/bjc.2013.116

6 McCowan C, Shearer J, Donnan PT, *et al.* Cohort study examining tamoxifen adherence and its relationship to mortality in women with breast cancer. *Br J Cancer* 2008;**99**:1763–8. doi:10.1038/sj.bjc.6604758

7 McCowan C, Wang S, Thompson AM, *et al.* The value of high adherence to tamoxifen in women with breast cancer: a community-based cohort study. *Br J Cancer* 2013;**109**:1172–80. doi:10.1038/bjc.2013.464

8 Winn AN, Dusetzina SB. The association between trajectories of endocrine therapy adherence and mortality among women with breast cancer. *Pharmacoepidemiol Drug Saf* 2016;**25**:953–9. doi:10.1002/pds.4012

9 Yood MU, Owusu C, Buist DSM, *et al.* Mortality impact of less-than-standard therapy in older breast cancer patients. *J Am Coll Surg* 2008;**206**:66–75. doi:10.1016/j.jamcollsurg.2007.07.015

10 Murphy CC, Bartholomew LK, Carpentier MY, *et al.* Adherence to adjuvant hormonal therapy among breast cancer survivors in clinical practice: a systematic review. *Breast Cancer Res Treat* 2012;**134**:459–78. doi:10.1007/s10549-012-2114-5

11 Owusu C, Buist DSM, Field TS, *et al.* Predictors of tamoxifen discontinuation among older women with estrogen receptor-positive breast cancer. *J Clin Oncol* 2008;**26**:549–55. doi:10.1200/JCO.2006.10.1022

12 Smith SG, Sestak I, Howell A, *et al.* Participant-reported symptoms and their effect on long-term adherence in the International Breast Cancer Intervention Study I (IBIS I). *J Clin Oncol* 2017; **35**:2666-2673. doi:10.1200/JCO.2016.71.7439

13 Sestak I, Smith SG, Howell A, *et al.* Early participant-reported symptoms as predictors of adherence to anastrozole in the International Breast Cancer Intervention Studies II. *Ann Oncol* 2017. **29**:504-509. doi: 10.1093/annonc/mdx713.

For peer review only - http://bmjopen.bmj.com/site/about/guidelines.xhtml

14 Kimmick G, Edmond SN, Bosworth HB, *et al.* Medication taking behaviors among breast cancer patients on adjuvant endocrine therapy. *The Breast* 2015;**24**:630–6. doi:10.1016/j.breast.2015.06.010

15 Wouters H, Stiggelbout AM, Bouvy ML, *et al.* Endocrine Therapy for Breast Cancer: Assessing an Array of Women's Treatment Experiences and Perceptions, Their Perceived Self-Efficacy and Nonadherence. *Clin Breast Cancer* 2014;**14**:460-467.e2. doi:10.1016/j.clbc.2014.04.005

16 Atkins L, Fallowfield L. Intentional and non-intentional non-adherence to medication amongst breast cancer patients. *Eur J Cancer* 2006;**42**:2271–6. doi:10.1016/j.ejca.2006.03.004

17 Green S, French D, Graham C, *et al.* Supporting adjuvant endocrine therapy adherence in women with breast cancer: the development of a complex behavioural intervention using Intervention Mapping guided by the Multiphase Optimisation Strategy. 2022. *BMC Health Serv Res* 2022;**22:**1081. https://doi.org/10.1186/s12913-022-08243-4

18 Hurtado-de-Mendoza A, Cabling ML, Lobo T, *et al.* Behavioral Interventions to Enhance Adherence to Hormone Therapy in Breast Cancer Survivors: A Systematic Literature Review. *Clin Breast Cancer* 2016;**16**:247-255.e3. doi:10.1016/j.clbc.2016.03.006

19 Heiney SP, Parker PD, Felder TM, *et al.* A systematic review of interventions to improve adherence to endocrine therapy. *Breast Cancer Res Treat* 2019. **173**:499-510 doi:10.1007/s10549-018-5012-7

20 Finitsis DJ, Vose BA, Mahalak JG, *et al.* Interventions to promote adherence to Endocrine Therapy among breast cancer survivors: A meta-analysis. *Psychooncology* 2018. **28**:255-263. doi: 10.1002/pon.4959.

21 Yap AF, Thirumoorthy T, Kwan YH. Systematic review of the barriers affecting medication adherence in older adults. *Geriatr Gerontol Int* 2016;**16**:1093–101. doi:10.1111/ggi.12616

22 Collins LM. *Optimization of Behavioral, Biobehavioral, and Biomedical Interventions: The Multiphase Optimization Strategy (MOST)*. Springer International Publishing 2018. //www.springer.com/gb/book/9783319722054 (accessed 11 Sep 2018).

23 Guastaferro K, Collins LM. Achieving the goals of translational science in public health intervention research: The multiphase optimization strategy (MOST). *Am J Public Health*. 2019;**109**:S128–S129.

24 Collins LM, Strayhorn JC, Vanness DJ. One view of the next decade of research on behavioral and biobehavioral approaches to cancer prevention and control: intervention optimization. *Transl Behav Med* 2021;**11**:1998–2008. doi:10.1093/tbm/ibab087

25 Guastaferro K, Collins LM. Optimization Methods and Implementation Science: An Opportunity for Behavioral and Biobehavioral Interventions. *Implement Res Pract* 2021;**2**:26334895211054364. doi:10.1177/26334895211054363

26 Eldredge LKB, Markham CM, Ruiter RA, *et al. Planning health promotion programs: an intervention mapping approach*. John Wiley & Sons 2016.

27 Moon Z, Moss-Morris R, Hunter MS, *et al.* Understanding tamoxifen adherence in women with breast cancer: A qualitative study. *Br J Health Psychol* 2017;**22**:978–97. doi:10.1111/bjhp.12266

For peer review only - http://bmjopen.bmj.com/site/about/guidelines.xhtml

28 Cahir C, Guinan E, Dombrowski SU, *et al.* Identifying the determinants of adjuvant hormonal therapy medication taking behaviour in women with stages I–III breast cancer: A systematic review and meta-analysis. *Patient Educ Couns* 2015;**98**:1524–39. doi:10.1016/j.pec.2015.05.013

29 Brett J, Fenlon D, Boulton M, *et al.* Factors associated with intentional and unintentional non-adherence to adjuvant endocrine therapy following breast cancer. *Eur J Cancer Care* 2018;**27**. doi:10.1111/ecc.12601

30 Toivonen KI, Williamson TM, Carlson LE, *et al.* Potentially Modifiable Factors Associated with Adherence to Adjuvant Endocrine Therapy among Breast Cancer Survivors: A Systematic Review. *Cancers* 2020;**13**:107. doi:10.3390/cancers13010107

31 Green SMC, Hall LH, Rousseau N, *et al.* Acceptability, fidelity and trial experience of four intervention components to support medication adherence in women with breast cancer: A process evaluation protocol for a pilot fractional factorial trial. Submitted.

32 Chan A-W, Tetzlaff JM, Altman DG, *et al.* SPIRIT 2013 statement: defining standard protocol items for clinical trials. *Ann Intern Med* 2013;**158**:200–7. doi:10.7326/0003-4819-158-3-201302050-00583

33 Eldridge SM, Chan CL, Campbell MJ, *et al.* CONSORT 2010 statement: extension to randomised pilot and feasibility trials. *BMJ* 2016;**355**:i5239. doi:10.1136/bmj.i5239

34 Hoffmann TC, Glasziou PP, Boutron I, *et al.* Better reporting of interventions: template for intervention description and replication (TIDieR) checklist and guide. *BMJ* 2014;**348**:g1687. doi:10.1136/bmj.g1687

35 Pullens MJJ, De Vries J, Roukema JA. Subjective cognitive dysfunction in breast cancer patients: a systematic review. *Psychooncology* 2010;**19**:1127–38. doi:10.1002/pon.1673

36 Underwood EA, Rochon PA, Moineddin R, *et al.* Cognitive sequelae of endocrine therapy in women treated for breast cancer: a meta-analysis. *Breast Cancer Res Treat* 2018;**168**:299–310. doi:10.1007/s10549-017-4627-4

37 Kirk MC, Hudis CA. Insight into barriers against optimal adherence to oral hormonal therapy in women with breast cancer. *Clin Breast Cancer* 2008;**8**:155–61. doi:10.3816/CBC.2008.n.016

38 Stilley CS, Bender CM, Dunbar-Jacob J, *et al.* The impact of cognitive function on medication management: Three studies. *Health Psychol* 2010;**29**:50–5. doi:10.1037/a0016940

39 Danner UN, Aarts H, de Vries NK. Habit formation and multiple means to goal attainment: repeated retrieval of target means causes inhibited access to competitors. *Pers Soc Psychol Bull* 2007;**33**:1367–79. doi:10.1177/0146167207303948

40 Gardner B, Lally P. Modelling Habit Formation and Its Determinants. In: Verplanken B, ed. *The Psychology of Habit: Theory, Mechanisms, Change, and Contexts*. Cham: Springer International Publishing 2018. 207–29. doi:10.1007/978-3-319-97529-0_12

41 Gardner B, Lally P, Wardle J. Making health habitual: the psychology of 'habit-formation' and general practice. *Br J Gen Pract* 2012;**62**:664–6. doi:10.3399/bjgp12X659466

42 Lally P, Gardner B. Promoting habit formation. *Health Psychol Rev* 2013;**7**:S137–58. doi:10.1080/17437199.2011.603640

For peer review only - http://bmjopen.bmj.com/site/about/guidelines.xhtml

43 Thakkar J, Kurup R, Laba T-L, *et al.* Mobile Telephone Text Messaging for Medication Adherence in Chronic Disease: A Meta-analysis. *JAMA Intern Med* 2016;**176**:340–9. doi:10.1001/jamainternmed.2015.7667

44 Finitsis DJ, Pellowski JA, Johnson BT. Text Message Intervention Designs to Promote Adherence to Antiretroviral Therapy (ART): A Meta-Analysis of Randomized Controlled Trials. *PLOS ONE* 2014;**9**:e88166. doi:10.1371/journal.pone.0088166

45 Free C, Phillips G, Galli L, *et al.* The effectiveness of mobile-health technology-based health behaviour change or disease management interventions for health care consumers: a systematic review. *PLoS Med* 2013;**10**:e1001362. doi:10.1371/journal.pmed.1001362

46 Ross XS, Gunn KM, Patterson P, *et al.* Mobile-Based Oral Chemotherapy Adherence–Enhancing Interventions: Scoping Review. *JMIR MHealth UHealth* 2018;**6**:e11724. doi:10.2196/11724

47 Green S, French D, Hall L, *et al. Co-development of a text messaging intervention to support adherence to adjuvant endocrine therapy in women with breast cancer: A mixed-methods approach (Preprint)*. 2022. doi:10.2196/preprints.38073

48 Michie S, Richardson M, Johnston M, *et al.* The behavior change technique taxonomy (v1) of 93 hierarchically clustered techniques: building an international consensus for the reporting of behavior change interventions. *Ann Behav Med* 2013;**46**:81–95.

49 Moon Z, Moss-Morris R, Hunter MS, *et al.* Barriers and facilitators of adjuvant hormone therapy adherence and persistence in women with breast cancer: a systematic review. *Patient Prefer Adherence* 2017;**11**:305–22. doi:10.2147/PPA.S126651

50 Pan Y, Heisig SR, von Blanckenburg P, *et al.* Facilitating adherence to endocrine therapy in breast cancer: stability and predictive power of treatment expectations in a 2-year prospective study. *Breast Cancer Res Treat* 2018;**168**:667–77. doi:10.1007/s10549-017-4637-2

51 Nestoriuc Y, von Blanckenburg P, Schuricht F, *et al.* Is it best to expect the worst? Influence of patients' side-effect expectations on endocrine treatment outcome in a 2-year prospective clinical cohort study. *Ann Oncol* 2016;**27**:1909–15. doi:10.1093/annonc/mdw266

52 Horne R, Chapman S, Parham R, *et al.* Understanding patients' adherence-related beliefs about medicines prescribed for long-term conditions: a meta-analytic review of the Necessity-Concerns Framework. *PLoS ONE* 2013;**8***:e80633. doi: 10.1371/journal.pone.0080633* .

53 Leventhal H, Diefenbach M, Leventhal EA. Illness cognition: Using common sense to understand treatment adherence and affect cognition interactions. *Cogn Ther Res* 1992;**16**:143–63. doi:10.1007/BF01173486

54 Gold M, Dunn LB, Phoenix B, *et al.* Co-occurrence of anxiety and depressive symptoms following breast cancer surgery and its impact on quality of life. *Eur J Oncol Nurs* 2016;**20**:97–105. doi:10.1016/j.ejon.2015.06.003

55 Martino G, Catalano A, Agostino RM, *et al.* Quality of life and psychological functioning in postmenopausal women undergoing aromatase inhibitor treatment for early breast cancer. *PloS One* 2020;**15**:e0230681. doi:10.1371/journal.pone.0230681

For peer review only - http://bmjopen.bmj.com/site/about/guidelines.xhtml

56 Jacobs JM, Walsh EA, Park ER, *et al.* The Patient's Voice: Adherence, Symptoms, and Distress Related to Adjuvant Endocrine Therapy After Breast Cancer. *Int J Behav Med* 2020;**27**:687–97. doi:10.1007/s12529-020-09908-2

57 Peddie N, Agnew S, Crawford M, *et al.* The impact of medication side effects on adherence and persistence to hormone therapy in breast cancer survivors: A qualitative systematic review and thematic synthesis. *Breast* 2021;**58**:147–59. doi:10.1016/j.breast.2021.05.005

58 Clancy C, Lynch J, OConnor P, *et al.* Breast cancer patients' experiences of adherence and persistence to oral endocrine therapy: A qualitative evidence synthesis. *Eur J Oncol Nurs* 2020;**44**:101706. doi:10.1016/j.ejon.2019.101706

59 Graham CD, McCracken LM, Harrison A, *et al.* Outlining an Acceptance and Commitment Therapy approach to treatment non-adherence. *Br J Health Psychol* 2021;:1–12.

60 Graham CD, Gouick J, Krahé C, *et al.* A systematic review of the use of Acceptance and Commitment Therapy (ACT) in chronic disease and long-term conditions. *Clin Psychol Rev* 2016;**46**:46–58. doi:10.1016/j.cpr.2016.04.009

61 Rose M, Graham CD, O'Connell N, *et al.* A randomised controlled trial of acceptance and commitment therapy for improving quality of life in people with muscle diseases. *Psychol Med* 2022;:1–14. doi:10.1017/S0033291722000083

62 Cella D, Fallowfield LJ. Recognition and management of treatment-related side effects for breast cancer patients receiving adjuvant endocrine therapy. *Breast Cancer Res Treat* 2008;**107**:167–80. doi:10.1007/s10549-007-9548-1

63 Coombes RC, Hall E, Gibson LJ, *et al.* A Randomized Trial of Exemestane after Two to Three Years of Tamoxifen Therapy in Postmenopausal Women with Primary Breast Cancer. *N Engl J Med* 2004;**350**:1081–92. doi:10.1056/NEJMoa040331

64 Goss PE, Ingle JN, Martino S, *et al.* A Randomized Trial of Letrozole in Postmenopausal Women after Five Years of Tamoxifen Therapy for Early-Stage Breast Cancer. *N Engl J Med* 2003;**349**:1793–802. doi:10.1056/NEJMoa032312

65 Howell A, Cuzick J, Baum M, *et al.* Results of the ATAC (Arimidex, Tamoxifen, Alone or in Combination) trial after completion of 5 years' adjuvant treatment for breast cancer. *Lancet* 2005;**365**:60–2. doi:10.1016/S0140-6736(04)17666-6

66 Jakesz R, Jonat W, Gnant M, *et al.* Switching of postmenopausal women with endocrine-responsive early breast cancer to anastrozole after 2 years' adjuvant tamoxifen: combined results of ABCSG trial 8 and ARNO 95 trial. *Lancet* 2005;**366**:455–62. doi:10.1016/S0140-6736(05)67059-6

67 Breast International Group (BIG) 1-98 Collaborative Group, Thürlimann B, Keshaviah A, *et al.* A comparison of letrozole and tamoxifen in postmenopausal women with early breast cancer. *N Engl J Med* 2005;**353**:2747–57. doi:10.1056/NEJMoa052258

68 Chlebowski RT, Kim J, Haque R. Adherence to endocrine therapy in breast cancer adjuvant and prevention settings. *Cancer Prev Res Phila Pa* 2014;**7**:378–87. doi:10.1158/1940-6207.CAPR-13-0389

For peer review only - http://bmjopen.bmj.com/site/about/guidelines.xhtml

69 Cella D, Fallowfield L, Barker P, *et al.* Quality of Life of Postmenopausal Women in the ATAC ("Arimidex", Tamoxifen, Alone or in Combination) Trial after Completion of 5 years' Adjuvant Treatment for Early Breast Cancer. *Breast Cancer Res Treat* 2006;**100**:273–84. doi:10.1007/s10549-006-9260-6

70 Fallowfield L, Cella D, Cuzick J, *et al.* Quality of Life of Postmenopausal Women in the Arimidex, Tamoxifen, Alone or in Combination (ATAC) Adjuvant Breast Cancer Trial. *J Clin Oncol* 2004;**22**:4261–71. doi:10.1200/JCO.2004.08.029

71 Ganz PA, Petersen L, Bower JE, *et al.* Impact of Adjuvant Endocrine Therapy on Quality of Life and Symptoms: Observational Data Over 12 Months From the Mind-Body Study. *J Clin Oncol* 2016;**34**:816–24. doi:10.1200/JCO.2015.64.3866

72 Schover LR, Baum GP, Fuson LA, *et al.* Sexual problems during the first 2 years of adjuvant treatment with aromatase inhibitors. *J Sex Med* 2014;**11**:3102–11. doi:10.1111/jsm.12684

73 Demissie S, Silliman RA, Lash TL. Adjuvant tamoxifen: predictors of use, side effects, and discontinuation in older women. *J Clin Oncol* 2001;**19**:322–8. doi:10.1200/JCO.2001.19.2.322

74 Kahn KL, Schneider EC, Malin JL, *et al.* Patient centered experiences in breast cancer: predicting long-term adherence to tamoxifen use. *Med Care* 2007;**45**:431–9. doi:10.1097/01.mlr.0000257193.10760.7f

75 Meggetto O, Maunsell E, Chlebowski R, *et al.* Factors Associated With Early Discontinuation of Study Treatment in the Mammary Prevention.3 Breast Cancer Chemoprevention Trial. *J Clin Oncol* 2017;**35**:629–35. doi:10.1200/JCO.2016.68.8895

76 Fleming L, Agnew S, Peddie N, *et al.* The impact of medication side effects on adherence and persistence to hormone therapy in breast cancer survivors: A quantitative systematic review. *Breast Edinb Scotl* 2022;**64**:63–84. doi:10.1016/j.breast.2022.04.010

77 Wheeler SB, Roberts MC, Bloom D, *et al.* Oncology providers' perspectives on endocrine therapy prescribing and management. *Patient Prefer Adherence* 2016;**10**:2007–19. doi:10.2147/PPA.S95594

78 Brett J, Boulton M, Fenlon D, *et al.* Adjuvant endocrine therapy after breast cancer: a qualitative study of factors associated with adherence. *Patient Prefer Adherence* 2018;**12**:291–300. doi:10.2147/PPA.S145784

79 Peate M, Saunders C, Cohen P, *et al.* Who is managing menopausal symptoms, sexual problems, mood and sleep disturbance after breast cancer and is it working? Findings from a large community-based survey of breast cancer survivors. *Breast Cancer Res Treat* 2021;**187**:427-435:doi:10.1007/s10549-021-06117-7

80 Hall LH, King NV, Graham CD, *et al.* Strategies to self-manage side-effects of adjuvant endocrine therapy among breast cancer survivors: an umbrella review of empirical evidence and clinical guidelines. *J Cancer Surviv Res Pract* 2021;**16**:1296-1338. doi:10.1007/s11764-021-01114-7

81 Hall LH, Clark J, Smith S, *et al.* Patient and healthcare professional co-development of an Acceptance and Commitment Therapy intervention to support Hormone Therapy decision-making and quality of life in women with breast cancer. J Psy Soc Oncol 2022;**40**; 407-424. https://doi.org/10.1080/07347332.2021.1955318

For peer review only - http://bmjopen.bmj.com/site/about/guidelines.xhtml

82 Morisky DE, Green LW, Levine DM. Concurrent and predictive validity of a self-reported measure of medication adherence. *Med Care* 1986;**24**:67–74.

83 Voils CI, King HA, Thorpe CT, *et al.* Content Validity and Reliability of a Self-Report Measure of Medication Nonadherence in Hepatitis C Treatment. *Dig Dis Sci* 2019;**64**:2784–97. doi:10.1007/s10620-019-05621-7

84 Aaronson NK, Ahmedzai S, Bergman B, *et al.* The European Organization for Research and Treatment of Cancer QLQ-C30: a quality-of-life instrument for use in international clinical trials in oncology. *J Natl Cancer Inst* 1993;**85**:365–76. doi:10.1093/jnci/85.5.365

85 Bjelic-Radisic V, Cardoso F, Cameron D, *et al.* An international update of the EORTC questionnaire for assessing quality of life in breast cancer patients: EORTC QLQ-BR45. *Ann Oncol* 2020;**31**:283–8. doi:10.1016/j.annonc.2019.10.027

86 Rabin R, de Charro F. EQ-5D: a measure of health status from the EuroQol Group. *Ann Med* 2001;**33**:337–43.

87 Rolffs JL, Rogge RD, Wilson KG. Disentangling Components of Flexibility via the Hexaflex Model: Development and Validation of the Multidimensional Psychological Flexibility Inventory (MPFI). *Assessment* 2018;**25**:458–82. doi:10.1177/1073191116645905

88 Brett J, Hulbert-Williams NJ, Fenlon D, *et al.* Psychometric properties of the Beliefs about Medicine Questionnaire–adjuvant endocrine therapy (BMQ-AET) for women taking AETs following early-stage breast cancer. *Health Psychol Open* 2017;**4**:2055102917740469. doi:10.1177/2055102917740469

89 Gardner B, Abraham C, Lally P, *et al.* Towards parsimony in habit measurement: Testing the convergent and predictive validity of an automaticity subscale of the Self-Report Habit Index. *Int J Behav Nutr Phys Act* 2012;**9**:102. doi:10.1186/1479-5868-9-102

90 Antony MM, Bieling PJ, Cox BJ, *et al.* Psychometric properties of the 42-item and 21-item versions of the Depression Anxiety Stress Scales in clinical groups and a community sample. *Psychol Assess* 1998;**10**:176–81. doi:10.1037/1040-3590.10.2.176

91 Cohen SR, Sawatzky R, Russell LB, *et al.* Measuring the quality of life of people at the end of life: The McGill Quality of Life Questionnaire-Revised. *Palliat Med* 2017;**31**:120–9. doi:10.1177/0269216316659603

92 UK Cancer Costs Questionnaire. UK Cancer Costs Questionnaire (accessed 12 Mar 2021: https://blogs.ed.ac.uk/ukcc/).

93 Sekhon M, Cartwright M, Francis JJ. Development of a theory-informed questionnaire to assess the acceptability of healthcare interventions. *BMC Health Serv Res* 2022;**22**:279. doi:10.1186/s12913-022-07577-3

94 Greene A, Elmer M, Ludlam S, *et al.* Evaluation of the Content Validity and Cross-Cultural Validity of the Study Participant Feedback Questionnaire (SPFQ). *Ther Innov Regul Sci* 2020;**54**:1522–33. doi:10.1007/s43441-020-00179-3

95 O'Neill L, Latchford G, McCracken L, *et al.* The Development of the Acceptance and Commitment Therapy Fidelity Measure (ACT-FM): A Delphi Study and Field Test. *J Context Behav Sci* 2019:111-118. doi:10.1016/j.jcbs.2019.08.008

For peer review only - http://bmjopen.bmj.com/site/about/guidelines.xhtml

96 Teare MD, Dimairo M, Shephard N, *et al.* Sample size requirements to estimate key design parameters from external pilot randomised controlled trials: a simulation study. *Trials* 2014;**15**:264. doi:10.1186/1745-6215-15-264

97 Vindrola-Padros C, Johnson GA. Rapid techniques in qualitative research: A critical review of the literature. *Qual Health Res* 2020;**30**:1596–1604.

98 Vindrola-Padros C, Chisnall G, Polanco N, *et al.* Iterative cycles in qualitative research: Introducing the RREAL Sheet as an innovative process. 2022: doi:https://doi.org/10.31219/osf.io/9dp2w

For peer review only

For peer review only - http://bmjopen.bmj.com/site/about/guidelines.xhtml

For peer review only

For peer review only - http://bmjopen.bmj.com/site/about/guidelines.xhtml

SPIRIT

STANDARD PROTOCOL ITEMS: RECOMMENDATIONS FOR INTERVENTIONAL TRIALS

SPIRIT 2013 Checklist: Recommended items to address in a clinical trial protocol and related documents*

Section/item	Item No	Description	Page
**Administrative information**			
Title	1	Descriptive title identifying the study design, population, interventions, and, if applicable, trial acronym	1
Trial registration	2a	Trial identifier and registry name. If not yet registered, name of intended registry	2, 21
	2b	All items from the World Health Organization Trial Registration Data Set	1, 2, 5, 6, 9, 10, 11, 19, 21, 23, T2
Protocol version	3	Date and version identifier	21
Funding	4	Sources and types of financial, material, and other support	23
Roles and responsibilities	5a	Names, affiliations, and roles of protocol contributors	1
	5b	Name and contact information for the trial sponsor	21
	5c	Role of study sponsor and funders, if any, in study design; collection, management, analysis, and interpretation of data; writing of the report; and the decision to submit the report for publication, including whether they will have ultimate authority over any of these activities	23
	5d	Composition, roles, and responsibilities of the coordinating centre, steering committee, endpoint adjudication committee, data management team, and other individuals or groups overseeing the trial, if applicable (see Item 21a for data monitoring committee)	20
**Introduction**			
Background and rationale	6a	Description of research question and justification for undertaking the trial, including summary of relevant studies (published and unpublished) examining benefits and harms for each intervention	4,5

For peer review only

For peer review only - http://bmjopen.bmj.com/site/about/guidelines.xhtml

	6b	Explanation for choice of comparators	8-11
Objectives	7	Specific objectives or hypotheses	5
Trial design	8	Description of trial design including type of trial (eg, parallel group, crossover, factorial, single group), allocation ratio, and framework (eg, superiority, equivalence, noninferiority, exploratory)	6
**Methods: Participants, interventions, and outcomes**			
Study setting	9	Description of study settings (eg, community clinic, academic hospital) and list of countries where data will be collected. Reference to where list of study sites can be obtained	6
Eligibility criteria	10	Inclusion and exclusion criteria for participants. If applicable, eligibility criteria for study centres and individuals who will perform the interventions (eg, surgeons, psychotherapists)	T2
Interventions	11a	Interventions for each group with sufficient detail to allow replication, including how and when they will be administered	8-11
	11b	Criteria for discontinuing or modifying allocated interventions for a given trial participant (eg, drug dose change in response to harms, participant request, or improving/worsening disease)	N/A
	11c	Strategies to improve adherence to intervention protocols, and any procedures for monitoring adherence (eg, drug tablet return, laboratory tests)	N/A
	11d	Relevant concomitant care and interventions that are permitted or prohibited during the trial	8
Outcomes	12	Primary, secondary, and other outcomes, including the specific measurement variable (eg, systolic blood pressure), analysis metric (eg, change from baseline, final value, time to event), method of aggregation (eg, median, proportion), and time point for each outcome. Explanation of the clinical relevance of chosen efficacy and harm outcomes is strongly recommended	11,12,17-19, T3, T4
Participant timeline	13	Time schedule of enrolment, interventions (including any run-ins and washouts), assessments, and visits for participants. A schematic diagram is highly recommended (see Figure)	7, 8, T3, T4

For peer review only - http://bmjopen.bmj.com/site/about/guidelines.xhtml

Sample size	14	Estimated number of participants needed to achieve study objectives and how it was determined, including clinical and statistical assumptions supporting any sample size calculations	19
Recruitment	15	Strategies for achieving adequate participant enrolment to reach target sample size	7, 8
**Methods: Assignment of interventions (for controlled trials)**			
Allocation:			
Sequence generation	16a	Method of generating the allocation sequence (eg, computer-generated random numbers), and list of any factors for stratification. To reduce predictability of a random sequence, details of any planned restriction (eg, blocking) should be provided in a separate document that is unavailable to those who enrol participants or assign interventions	8
Allocation concealment mechanism	16b	Mechanism of implementing the allocation sequence (eg, central telephone; sequentially numbered, opaque, sealed envelopes), describing any steps to conceal the sequence until interventions are assigned	8
Implementation	16c	Who will generate the allocation sequence, who will enrol participants, and who will assign participants to interventions	8
Blinding (masking)	17a	Who will be blinded after assignment to interventions (eg, trial participants, care providers, outcome assessors, data analysts), and how	8
	17b	If blinded, circumstances under which unblinding is permissible, and procedure for revealing a participant's allocated intervention during the trial	N/A
**Methods: Data collection, management, and analysis**			
Data collection methods	18a	Plans for assessment and collection of outcome, baseline, and other trial data, including any related processes to promote data quality (eg, duplicate measurements, training of assessors) and a description of study instruments (eg, questionnaires, laboratory tests) along with their reliability and validity, if known. Reference to where data collection forms can be found, if not in the protocol	11, 12, 17, 18, 19, 20

	18b	Plans to promote participant retention and complete follow-up, including list of any outcome data to be collected for participants who discontinue or deviate from intervention protocols	7, 8, 11
Data management	19	Plans for data entry, coding, security, and storage, including any related processes to promote data quality (eg, double data entry; range checks for data values). Reference to where details of data management procedures can be found, if not in the protocol	20
Statistical methods	20a	Statistical methods for analysing primary and secondary outcomes. Reference to where other details of the statistical analysis plan can be found, if not in the protocol	20, 21
	20b	Methods for any additional analyses (eg, subgroup and adjusted analyses)	20, 21
	20c	Definition of analysis population relating to protocol non-adherence (eg, as randomised analysis), and any statistical methods to handle missing data (eg, multiple imputation)	20, 21
**Methods: Monitoring**			
Data monitoring	21a	Composition of data monitoring committee (DMC); summary of its role and reporting structure; statement of whether it is independent from the sponsor and competing interests; and reference to where further details about its charter can be found, if not in the protocol. Alternatively, an explanation of why a DMC is not needed	20
	21b	Description of any interim analyses and stopping guidelines, including who will have access to these interim results and make the final decision to terminate the trial	N/A
Harms	22	Plans for collecting, assessing, reporting, and managing solicited and spontaneously reported adverse events and other unintended effects of trial interventions or trial conduct	22
Auditing	23	Frequency and procedures for auditing trial conduct, if any, and whether the process will be independent from investigators and the sponsor	N/A
**Ethics and dissemination**			
Research ethics approval	24	Plans for seeking research ethics committee/institutional review board (REC/IRB) approval	21, 22

Protocol amendments	25	Plans for communicating important protocol modifications (eg, changes to eligibility criteria, outcomes, analyses) to relevant parties (eg, investigators, REC/IRBs, trial participants, trial registries, journals, regulators)	21
Consent or assent	26a	Who will obtain informed consent or assent from potential trial participants or authorised surrogates, and how (see Item 32)	8
	26b	Additional consent provisions for collection and use of participant data and biological specimens in ancillary studies, if applicable	N/A
Confidentialit y	27	How personal information about potential and enrolled participants will be collected, shared, and maintained in order to protect confidentiality before, during, and after the trial	8
Declaration of interests	28	Financial and other competing interests for principal investigators for the overall trial and each study site	23
Access to data	29	Statement of who will have access to the final trial dataset, and disclosure of contractual agreements that limit such access for investigators	22
Ancillary and post-trial care	30	Provisions, if any, for ancillary and post-trial care, and for compensation to those who suffer harm from trial participation	N/A
Disseminatio n policy	31a	Plans for investigators and sponsor to communicate trial results to participants, healthcare professionals, the public, and other relevant groups (eg, via publication, reporting in results databases, or other data sharing arrangements), including any publication restrictions	22
	31b	Authorship eligibility guidelines and any intended use of professional writers	23
	31c	Plans, if any, for granting public access to the full protocol, participant-level dataset, and statistical code	22
**Appendices**			
Informed consent materials	32	Model consent form and other related documentation given to participants and authorised surrogates	Included
Biological specimens	33	Plans for collection, laboratory evaluation, and storage of biological specimens for genetic or molecular analysis in the current trial and for future use in ancillary studies, if applicable	N/A

For peer review only

*It is strongly recommended that this checklist be read in conjunction with the SPIRIT 2013 Explanation & Elaboration for important clarification on the items. Amendments to the protocol should be tracked and dated. The SPIRIT checklist is copyrighted by the SPIRIT Group under the Creative Commons "Attribution-NonCommercial-NoDerivs 3.0 Unported" license.

For peer review only

For peer review only - http://bmjopen.bmj.com/site/about/guidelines.xhtml

CONSORT 2010 checklist of information to include when reporting a pilot or feasibility trial*

Section/Topic	Item No	Checklist item	Reported on page No
**Title and abstract**			
	1a	Identification as a pilot or feasibility randomised trial in the title	1
	1b	Structured summary of pilot trial design, methods, results, and conclusions (for specific guidance see CONSORT abstract extension for pilot trials)	2
**Introduction**			
Background and objectives	2a	Scientific background and explanation of rationale for future definitive trial, and reasons for randomised pilot trial	4-5
	2b	Specific objectives or research questions for pilot trial	5
**Methods**			
Trial design	3a	Description of pilot trial design (such as parallel, factorial) including allocation ratio	5
	3b	Important changes to methods after pilot trial commencement (such as eligibility criteria), with reasons	N/A
Participants	4a	Eligibility criteria for participants	6 (Table 2)
	4b	Settings and locations where the data were collected	6
	4c	How participants were identified and consented	6-7
Interventions	5	The interventions for each group with sufficient details to allow replication, including how and when they were actually administered	7-9
Outcomes	6a	Completely defined prespecified assessments or measurements to address each pilot trial objective specified in 2b, including how and when they were assessed	9-13
	6b	Any changes to pilot trial assessments or measurements after the pilot trial commenced, with reasons	N/A
	6c	If applicable, prespecified criteria used to judge whether, or how, to proceed with future definitive trial	Table 5
Sample size	7a	Rationale for numbers in the pilot trial	13
	7b	When applicable, explanation of any interim analyses and stopping guidelines	N/A
Randomisation:			
Sequence generation	8a	Method used to generate the random allocation sequence	7
	8b	Type of randomisation(s); details of any restriction (such as blocking and block size)	7
Allocation concealment mechanism	9	Mechanism used to implement the random allocation sequence (such as sequentially numbered containers), describing any steps taken to conceal the sequence until interventions were assigned	7

For peer review only

Implementation	10	Who generated the random allocation sequence, who enrolled participants, and who assigned participants to interventions	6-7
Blinding	11a	If done, who was blinded after assignment to interventions (for example, participants, care providers, those assessing outcomes) and how	7
	11b	If relevant, description of the similarity of interventions	7-9
Statistical methods	12	Methods used to address each pilot trial objective whether qualitative or quantitative	
**Results**			
Participant flow (a diagram is strongly recommended)	13a	For each group, the numbers of participants who were approached and/or assessed for eligibility, randomly assigned, received intended treatment, and were assessed for each objective	N/A
	13b	For each group, losses and exclusions after randomisation, together with reasons	N/A
Recruitment	14a	Dates defining the periods of recruitment and follow-up	N/A
	14b	Why the pilot trial ended or was stopped	N/A
Baseline data	15	A table showing baseline demographic and clinical characteristics for each group	N/A
Numbers analysed	16	For each objective, number of participants (denominator) included in each analysis. If relevant, these numbers should be by randomised group	N/A
Outcomes and estimation	17	For each objective, results including expressions of uncertainty (such as 95% confidence interval) for any estimates. If relevant, these results should be by randomised group	N/A
Ancillary analyses	18	Results of any other analyses performed that could be used to inform the future definitive trial	N/A
Harms	19	All important harms or unintended effects in each group (for specific guidance see CONSORT for harms)	N/A
	19a	If relevant, other important unintended consequences	N/A
**Discussion**			
Limitations	20	Pilot trial limitations, addressing sources of potential bias and remaining uncertainty about feasibility	N/A
Generalisability	21	Generalisability (applicability) of pilot trial methods and findings to future definitive trial and other studies	N/A
Interpretation	22	Interpretation consistent with pilot trial objectives and findings, balancing potential benefits and harms, and considering other relevant evidence	N/A
	22a	Implications for progression from pilot to future definitive trial, including any proposed amendments	
**Other information**			
Registration	23	Registration number for pilot trial and name of trial registry	1, 6
Protocol	24	Where the pilot trial protocol can be accessed, if available	15
Funding	25	Sources of funding and other support (such as supply of drugs), role of funders	16
	26	Ethical approval or approval by research review committee, confirmed with reference number	15

For peer review only

Citation: Eldridge SM, Chan CL, Campbell MJ, Bond CM, Hopewell S, Thabane L, et al. CONSORT 2010 statement: extension to randomised pilot and feasibility trials. BMJ. 2016;355.

*We strongly recommend reading this statement in conjunction with the CONSORT 2010, extension to randomised pilot and feasibility trials, Explanation and Elaboration for important clarifications on all the items. If relevant, we also recommend reading CONSORT extensions for cluster randomised trials, non-inferiority and equivalence trials, non-pharmacological treatments, herbal interventions, and pragmatic trials. Additional extensions are forthcoming: for those and for up to date references relevant to this checklist, see www.consort-statement.org.

For peer review only

The TIDieR (Template for Intervention Description and Replication) Checklist*:

Information to include when describing an intervention and the location of the information

Nº	What	Details
**1**	**Name**	Refining and Optimising a behavioural intervention to support endocrine therapy adherence: The ROSETA Pilot Trial.
**2**	**Why: Rationale, theory, goal**	Adjuvant hormone therapies are prescribed at the end of hospital-based breast cancer treatment in order to prevent recurrences and all-cause mortality. However adherence to these medications is often poor, due to multiple factors, including forgetting, beliefs about medications, intolerable side effects and psychological distress. This time during the cancer journey is also particularly challenging, as women are transitioning from 'patient' to 'survivor'. They also report a lack of support during this time, post hospital discharge.<br><br>Previous adherence interventions have tended to consist of solely educational based interventions, that are not grounded in theory, and did not target the factors commonly associated with medication adherence. Given the wide range of barriers to adherence in this population, it is perhaps unsurprising that previous interventions have shown limited effectiveness. An alternative strategy is to design a complex intervention, with multiple components that can target a range of factors that have been highlighted as barriers to adherence.<br><br>*Memory and forgetting*<br><br>Mobile phone-based interventions are well suited to tackle forgetfulness as a barrier to adherence, through reminders and promotion of habit formation. SMS messages have been shown to be effective in improving medication adherence in other chronic illnesses but have not been widely tested in cancer patients.<br><br>*Medication schemas*<br><br>Accurate information about the necessity and risks of AET has the potential to increase women's perceptions of their need for AET, and to reduce unfounded concerns about the medication. In addition, women with breast cancer have stated that they would

For peer review only

		like more accurate information about AET to overcome unfounded concerns.<br><br>*Psychological Flexibility*<br><br>Acceptance and Commitment Therapy (ACT) has been shown to improve outcomes in those living with chronic illness, chronic pain, and cancer. ACT aims to increase a participant's awareness of their personal values, and to undertake more of the behaviours that support these values – a process that often involves developing a willingness to have painful thoughts and feelings (such as medication side-effects). ACT targets psychological flexibility, which can improve functioning during objectively difficult circumstances, and can often reduce psychological distress as a by-product.<br><br>*Living with Side effects*<br><br>One of the most commonly cited barriers to AET adherence is the impact of side effects, and the lack of support for management of these is commonly cited. There are a number of strategies for these side effects that have the potential to be effective in alleviating symptoms. However, these are typically not presented in a patient-friendly manner.<br><br>Given the above, we have co-designed four intervention components for women with breast cancer who have been prescribed adjuvant endocrine therapies; SMS reminder messages to target forgetfulness, an information leaflet to promote formation of accurate beliefs, ACT therapy sessions to increase psychological flexibility, and a side-effect management website to support living with side effects. The aim of the intervention components are to support medication adherence to hormone therapy. Participants will be randomised to receive none, or a combination of one or more of these four components.
**3**	**What Materials**	*Participants randomised to receive SMS component*<br><br>Participants received 43 SMS messages over four months. This included three opening messages, one closing message, 36 messages related to behaviour change techniques aiming to

For peer review only

		promote habit formation, and 3 messages (sent after 1, 2 and 3 months) as a reminder that participants can stop any further SMS messages being sent by emailing the ROSETA team. The content of the SMS messages was co-developed with experts in behaviour change and/or medication adherence, and women who have experienced breast cancer.<br><br>*Participants randomised to receive information leaflet*<br><br>Participants received an information leaflet containing detailed information about AET. This included information about how the medication works (with diagrams to supplement), information about the benefits and side effects of AET, answers to common concerns that women have, and quotes from women with experience of taking AET.<br><br>*ACT*<br><br>*Participants randomised to ACT sessions*<br><br>Participants were emailed a participant manual consisting of information about the ACT skill and home practice tasks, in addition to corresponding audio files to assist with the home practice tasks. Each of the four modules focused on a different ACT-based skill:<br>• Module 1: Mindfulness and unhooking<br>• Module 2: Following your values<br>• Module 3: Taking an observer perspective<br>• Module 4: Recap, reflection, and staying committed<br><br>*Therapists delivering ACT sessions*<br><br>Therapists delivering the intervention received two half days of bespoke training delivered by clinical psychologists with ACT experience. Alongside this, they received a training manual, with information about ACT generally, and specific session plans for the intervention sessions.<br><br>*Participants randomised to receive access to side-effect website*

For peer review only

		Participants allocated to receive the website will receive access to a bespoke website containing information and strategies for self-management of side effects, and signposting to further sources of support.
**4**	**What Procedures**	***Intervention Delivery***<br><br>*Participants randomised to receive SMS component*<br><br>Participants received 43 SMS messages over four months. This included three opening messages, one closing message, 36 messages related to behaviour change techniques aiming to promote habit formation, and 3 messages (sent after 1, 2 and 3 months) as a reminder that participants can stop any further SMS messages being sent by emailing the ROSETA team. The content of the SMS messages was co-developed with experts in behaviour change and/or medication adherence, and women who have experienced breast cancer.<br><br>The 36 messages relating to behaviour change techniques were sent on the following schedule:<br><br>– Daily messages for 2 weeks<br>– Two messages per week for 8 weeks<br>– Weekly messages for 6 weeks<br><br>*Participants randomised to receive information leaflet*<br><br>Participants were sent the information leaflet by email immediately after randomisation.<br><br>*Participants randomised to receive ACT*<br><br>4x guided self-help modules consisting of information about ACT, home practice exercises and corresponding audio files<br><br>1x 15-minute individual introductory session with a psychologist<br><br>3 x 25 minute individual support sessions with a psychologist to discuss the module completed over the past week, their experiences of the home practice exercises, and to allow discussion and problem solving of any difficulties that arose.<br><br>1x 15-minute closing session with a psychologist

For peer review only

		Participants randomised to receive access to side-effect website<br><br>Participants were given login details of the website immediately after randomisation.<br><br>*Clinician Training*<br><br>(See section 5, below, for information on this)<br><br>*Evaluation of the Clinician Training*<br><br>Recordings of each therapists first session were reviewed by Dr Graham, using the ACT-FM therapist stance subscale.<br><br>Clinician fidelity was evaluated using the ACT-FM therapist stance subscale completed by an external rater. 10% of recorded sessions were evaluated.<br><br>Clinician fidelity to intervention procedures involved clinician self-rating using a procedural fidelity checklist.<br><br>*Evaluation of the Intervention Components*<br><br>Adherence, quality of life/ symptom burden, psychological distress, psychological flexibility, habitual behaviour of medication taking and medication beliefs were all measured pre and post intervention. Acceptability of each intervention component was assessed. Self reported engagement of intervention components was monitored. SMS delivery, and website use was tracked.<br><br>*Support activities*<br><br>Recruitment and consent of participants
**5**	**Who provided**	*SMS messages*<br><br>The CTRU sent all SMS messages to participants.<br><br>*Information Leaflet*<br><br>The information leaflet was sent to participants by the site.

For peer review only

		ACT<br><br>The therapists who delivered the intervention underwent training in delivering Acceptance and Commitment Therapy. The training was delivered by Dr Chris Graham (CG), who has expertise in ACT applied to chronic disease. Training included teaching about ACT and practice of intervention-specific therapy methods. This course consisted of two half days of training.<br><br>Each site's therapists had a varied background that may or may not have included previous ACT training prior to our delivered training programme. However, all session leads were Health and Care Professional Council (HCPC) or UKCP registered practitioner psychologists or Psychotherapists (Clinical, Health or Counselling Psychologist or Psychotherapists) who worked with breast cancer patients in a hospital setting.<br><br>*Website*<br><br>Access to the bespoke website was provided by the site.
**6**	**How: mechanisms of delivery**	*Participants randomised to receive SMS component*<br><br>SMS messages were sent in an automated fashion by the CTRU to the participants mobile phone based on the following schedule:<br><br>– Daily messages for 2 weeks<br>– Two messages per week for 8 weeks<br>– Weekly messages for 6 weeks<br><br>In addition one message was sent after months 1, 2 and 3 as a reminder that participants can stop any further SMS messages being sent by emailing the ROSETA team.<br><br>*Participants randomised to receive information leaflet*<br><br>Participants were sent the information leaflet electronically immediately after randomisation and were able to read this as they wished.

		Participants randomised to receive ACT<br>The individual sessions (5 in total) were delivered via phone or video conferencing.<br>The participant manual containing information about each module, home practice tasks, and audio files were emailed to each participant by the therapist following each session.<br><br>*Participants randomised to receive access to side-effect website*<br>Participants were given a login to the website immediately after randomisation and were able to use this as they wished.
**7**	**Where: location of delivery**	*SMS Messages*<br>Not applicable.<br><br>*Information Leaflet*<br>Not applicable.<br><br>*ACT*<br>All sessions were delivered remotely via phone or videoconferencing.<br><br>*Website*<br>Not applicable
**8**	**When and how much**	*Participants randomised to receive SMS component*<br>SMS messages were sent by the CTRU based on the following schedule:<br>– Daily messages for 2 weeks<br>– Two messages per week for 8 weeks<br>– Weekly messages for 6 weeks<br>In addition three opening messages, one closing message and, one message after months 1,2 and 3 were sent reminding participants

For peer review only

		that they could stop any further SMS messages being sent via emailing the ROSETA team.<br><br>*Participants randomised to receive information leaflet*<br><br>Participants were sent the information leaflet electronically immediately after randomisation and were able to read this as they wished.<br><br>*Participants randomised to receive ACT*<br><br>The introductory session lasted 15 minutes, three subsequent sessions lasted 25 minutes, and the final closing session lasted 15 minutes. Participants had their first session within four weeks of randomisation. The therapy sessions were held weekly.<br><br>*Participants randomised to receive access to side-effect website*<br><br>Participants were given a login to the website immediately after randomisation and were able to use this as they wished.
**9**	**Tailoring**	*SMS*<br><br>The same SMS messages were sent in the same order to each participant.<br><br>*Information Leaflet*<br><br>The same information leaflet was sent to each participant.<br><br>*ACT*<br><br>Although there is a set session plan to follow, detailing specific exercises and tasks for each session, the therapy itself is quite flexible. As such, the deliverer may adapt the content to ensure it's relevant to each participant (e.g. through discussing specific individuals' values, goals, and behaviours).

For peer review only

		Website<br><br>The website was the same for each participant.
**10***	**Modifications**	*<To be completed post study completion>*
**11**	**How well (planned)**	*SMS*<br><br>Successful delivery and receipt of the SMS messages will be recorded by the CTRU, alongside the number of SMS messages that were unable to be delivered. Participants will answer a single item asking whether they received the SMS messages, and another item asking how many of the SMS messages they read. Semi-structured interviews were conducted to understand fidelity of receipt and enactment of the messages.<br><br>*Information Leaflet*<br><br>The number of information leaflets sent out to participants was recorded. This will be recorded by the site when each information leaflet is sent out.<br><br>Participants were asked to self-report whether they received the information leaflet, and how much of the information leaflet they read. Semi-structured interviews were conducted with participants to understand the fidelity of receipt and enactment of the information leaflet.<br><br>*ACT*<br><br>Clinician fidelity to competently deliver the intervention in line with ACT was assessed by an external rater with a background in ACT. They completed the ACT-FM therapist stance subscale checklist whilst listening to the audio recording of 10% of sessions. A score of >4 on ACT consistent behaviours and <5 on ACT inconsistent behaviours is considered competent.<br><br>Additionally, an intervention specific metric of "Procedural Fidelity" was included, which measures other aspects of the intervention that are important for treatment fidelity but are not ACT-specific (e.g. reflecting on home practice tasks, sending module content etc). Therapists will complete the procedural fidelity checklist following each session. A percentage score is created for each session by dividing the score achieved by the

		maximum possible score achievable within that session and multiplying by 100.<br><br>Fidelity of ACT training was monitored through Dr Graham assessing the recording of each therapists first ACT session, and rating competency based on the ACT-FM therapist stance subscale. A score of >4 on ACT consistent behaviours and <5 on ACT inconsistent behaviours is considered competent. Semi-structured interviews with psychologists assessed the fidelity of training and delivery of the ACT component.<br><br>Participant fidelity to the ACT component was monitored by recording the number of sessions attended, missed and cancelled. The therapist additionally reported how much of the module materials the participant had read and engaged with (participant manual, audio files and home practice tasks). Participants self-reported receipt of the module content, self-reported engagement with the participant manual, audio files and home practice tasks. Semi-structured interviews additionally assessed fidelity of receipt and enactment.<br><br>Additionally 10% of recorded sessions were evaluated by an independent reviewer in order to review and assess the recording of a participants engagement in home work tasks by the therapist.<br><br>*Website*<br><br>Website data was tracked for each participant, including number of logins, time spent on pages, videos watched and clicked links. Participants were asked a single item about their engagement with the website. Fidelity of receipt and enactment were additionally assessed through semi-structured interviews.
**12***	**How well (actual)**	*<To be completed post study>*

For peer review only

Delete this line, then print first page on Trust- headed paper

NIHR | National Institute for Health Research

UNIVERSITY OF LEEDS

ROSETA
Refining and Optimising a behavioural intervention to Support Endocrine Therapy Adherence

Participant ID: [CTRU Insert]	Initials:
Date of Birth:	NHS Number:
ISRCTN: <<INSERT>>	Principal Investigator:

PARTICIPANT CONSENT FORM

	*Please **initial** each box below*
1. I have read and understand the information sheet dated <<INSERT DATE>> (version X.0) for the above study and have had the opportunity to ask questions.	
2. I understand taking part in this study is voluntary and I am free to withdraw at any time without it affecting my medical care or legal rights.	
3. I understand that if I withdraw from the study, the data collected from me up until that point will still be retained and analysed.	
4. I understand that relevant sections of my medical records and/or study data may be looked at by responsible individuals from the research team, the sponsor (University of Leeds), Leeds Clinical Trials Research Unit, relevant third parties or from regulatory authorities where it is relevant to my participation. I give permission for these individuals to access my records.	
5. I understand that if during this study my clinical care team determine that I have lost my ability to make my own decisions, I will be withdrawn from the study and no further study information will be collected. I agree that data collected up until this point will remain on file and will be included in the analysis.	
6. I consent to the secure transfer, storage and use of paper and electronic personal information, for the purposes of this study to the Leeds Clinical Trials Research Unit, or relevant third parties. I understand that any information that could identify me will be kept strictly confidential and that no personal information will be included in the study report or other publication.	
7. I give consent for my personal details (which will include my full name, date of birth, gender, and NHS number) to be shared with NHS Digital for the purpose of the research team obtaining	

For peer review only - http://bmjopen.bmj.com/site/about/guidelines.xhtml

Delete this line, then print first page on Trust- headed paper

prescribing and dispensing data for my adjuvant endocrine therapy medication.		
8. If randomised to receive Acceptance and Commitment Therapy, I give consent for the sessions to be audio recorded.		
9. If randomised to have access to the website, I consent for my use of the website to be tracked, which will include capturing data on pages visited, links downloaded and number of visits to the website.		
10. I agree to a copy of this consent form being sent to Leeds CTRU.		
11. I agree to my General Practitioner being notified of my participation in this study.		
12. I understand that my confidentiality will be kept unless, during the course of the study, the researcher/clinical team, has reason to believe that I am at risk of harming myself or others and this may involve a member of the research/clinical team contacting me.		
13. I agree that record-level information (data related to a single individual) collected about me may be used to support other research in the future but that I will not be directly identified. Data may be shared anonymously with other researchers.		
14. I will provide the research team with an email address so I can receive log in details for the online completion of questionnaires. I agree that my email address can also be used to send me information relevant to the interventions I may be randomised to in the study.		
15. I will provide the research team with a mobile telephone number, and understand that I may receive SMS messages to prompt me when follow up questionnaires are being sent and need to be completed. I agree that if I am randomised to receive the SMS intervention, I will also receive messages reminding to me to take my AET medication. I understand that to do so my phone number will be shared with a SMS service provider.		
16. I agree to take part in the study.		

Optional results consent

	*Please **initial** relevant box below*	
17. I would like to be contacted about the results of this study using the contact details I have provided.	Yes	
	No	

**Optional interview consent**	*Please **initial** relevant box below*	
18. I am happy to be contacted by a member of the research team about taking part in the follow up semi-structured interview and I	Yes	

For peer review only

For peer review only - http://bmjopen.bmj.com/site/about/guidelines.xhtml

Delete this line, then print first page on Trust- headed paper

understand taking part in this is separate to the intervention and is not mandatory.	No	

Face-To-Face

Participant- Please sign and date:

Signature……………………………………………………………………………

Name (block capitals)…………………………………………….……………………

Date…………………………………………….……………………………………

For peer review only

Please now return this form to the Research Nurse

To be completed on receipt by the Research Nurse;

Research Nurse:

I have explained the study to the above participant and she has indicated her willingness to participate. I have placed a copy of this consent form in her medical notes.

Signature……………………………………...……………………………………

Name (block capitals)…………………………………………….………………

Date…………………………………………….……………………………………

For peer review only - http://bmjopen.bmj.com/site/about/guidelines.xhtml

Delete this line, then print first page on Trust- headed paper

Telephone Consent

Research Nurse:

I have explained the study and read the statements to the above named participant. She has indictated her willingness to participate and agreed to each compulsory statement, so I have initialled and signed on her behalf.

Signature……………………………………..……………………………………

Name (block capitals)………………………………………………….…………………

Date…………………………………………………….……………………………………

Note for Research Nurse (Original copy to be sent to the CTRU; 1 copy returned to the participant; 1 copy to be stored in Investigator Site File, 1 copy for treating clinician (where appropriate))

For peer review only - http://bmjopen.bmj.com/site/about/guidelines.xhtml
For peer review only
