# Supplementary material for: Refining and optimising a behavioural intervention to support endocrine therapy adherence (ROSETA) in UK women with breast cancer: protocol for a pilot fractional factorial trial
Source: BMJ Open. 2023 Feb 3;13(2):e069971. doi: 10.1136/bmjopen-2022-069971 (PMC9900066; doi:10.1136/bmjopen-2022-069971)
Supplement: Reviewer comments [file bmjopen-2022-069971.reviewer_comments.pdf]

PEER REVIEW HISTORY

BMJ Open publishes all reviews undertaken for accepted manuscripts. Reviewers are asked to complete a checklist review form (**http://bmjopen.bmj.com/site/about/resources/checklist.pdf**) and are provided with free text boxes to elaborate on their assessment. These free text comments are reproduced below.

ARTICLE DETAILS

**TITLE (PROVISIONAL)**	Refining and Optimising a behavioural intervention to Support Endocrine Therapy Adherence (ROSETA) in UK women with breast cancer: protocol for a pilot fractional factorial trial
**AUTHORS**	Smith, Samuel; Green, Sophie M. C.; Ellison, Rachel; Foy, Robbie; Graham, Christopher D.; Mason, Ellen; French, David; Hall, Louise; Wilkes, Hollie; McNaught, Emma; Raine, Erin; Walwyn, Rebecca; Howdon, Daniel; Clark, Jane; Rousseau, Nikki; Buxton, Jacqueline; Moore, Sally J. L.; Parbutt, Catherine; Velikova, Galina; Farrin, Amanda; Collinson, Michelle

This article was not externally reviewed at BMJ Open. Protocols that have been independently assessed prior to submission to BMJ Open are usually fast-tracked to publication on the grounds that further substantial changes will not be possible. This independent assessment will usually be external, independent review for both a substantial grant award from a non-commercial or government funder and ethics approval.
